# Global, regional and national burden of neurodegenerative diseases attributable to smoking: A descriptive study based on the Global Burden of Disease Study 2021

**DOI:** 10.18332/tid/217009

**Published:** 2026-03-09

**Authors:** Zeyu Yang, Zhaoyi Jing, Qingyu Song, Xiao Ding, Xiaohui Lu

**Affiliations:** 1 Shandong University of Traditional Chinese Medicine, Jinan, China; 2 Shanghai University of Traditional Chinese Medicine, Shanghai, China; 3 Affiliated Hospital of Shandong University of Traditional Chinese Medicine, Jinan, China

**Keywords:** neurodegenerative diseases, smoking, inequality analysis, decomposition analysis, frontier analysis

## Abstract

**INTRODUCTION:**

Neurodegenerative diseases impose a significant healthcare burden worldwide. Within the context of smoking, the risk of dementia and multiple sclerosis significantly increases. However, the global epidemiological characteristics of smoking-induced neurodegenerative diseases remain unclear.

**METHODS:**

This study, based on data from the GBD 2021 database, quantified the burden of smoking-attributable neurodegenerative diseases globally, across 5 SDI regions, 21 regions, and 204 countries and territories. The analysis was stratified by age and sex and covered the period from 1990 to 2021, utilizing a descriptive study design. The analysis incorporates transnational inequality assessment, decomposition techniques, and frontier analysis. Projections of the neurodegenerative diseases attributable to smoking burden for 2035 are also presented.

**RESULTS:**

From a global perspective, smoking-attributable deaths and disability-adjusted life years from both dementia and multiple sclerosis have risen. Males consistently outnumber females across all age groups. Significant health inequalities persist, with Lebanon and Denmark exhibiting the highest disease burden for dementia and multiple sclerosis, respectively. Demographic factors emerge as key drivers of this burden.

**CONCLUSIONS:**

This study underscores the persistent global health challenges posed by smoking-attributable dementia and multiple sclerosis. These findings underscore the compelling need for targeted health policies and interventions. Furthermore, future epidemiological investigations focused on high-burden regions are warranted.

## INTRODUCTION

Neurodegenerative diseases such as Alzheimer’s disease (AD), other types of dementia, and multiple sclerosis (MS) represent a growing global health challenge, affecting millions of individuals and their families worldwide^1,2^. These conditions not only impose significant physical and mental burdens on patients but also result in substantial societal and economic costs due to increased healthcare expenditures and caregiving demands^3^. Current treatment approaches primarily focus on pharmacological interventions and rehabilitation strategies^4^. However, the complex etiology and individual variability associated with these diseases limit the effectiveness of existing therapeutic methods, resulting in a substantial disease burden. A robust framework is currently lacking for the systematic integration of real-time risk surveillance^5-7^. Such deficiencies significantly hinder the development of risk-stratified preventive paradigms. These limitations further compound challenges in public health interventions.

Epidemiological studies have identified smoking as a modifiable risk factor associated with several neurodegenerative diseases^8,9^. Systematic macro-epidemiological evidence from a global perspective remains lacking. Research suggests that smoking may influence neurodegenerative processes through various biological mechanisms^10^. Elucidating global disease dynamics within this distinct population subgroup could inform targeted public health strategies to mitigate the burden of neurodegenerative disorders.

The aim of this study is to leverage data from the Global Burden of Disease 2021 database to conduct an assessment of temporal trends and epidemiological patterns of smoking-attributable neurodegenerative diseases, Alzheimer’s disease, other dementias, and multiple sclerosis, across global, regional, and national scales, with a particular focus on age- and sex-stratified disparities.

Notably, this study represents the first international inequality and decomposition analysis conducted in populations with neurodegenerative diseases attributable to smoking exposure. It quantifies disparities in this disease burden and projects their trends through 2035. By elucidating the temporal dynamics of neurodegenerative diseases among smokers, identifying systemic health inequalities, and defining modifiable risk factors, our findings are intended to inform the development of evidence-based and targeted public health strategies.

## METHODS

### Data acquisition and preprocessing

The GBD 2021 is a comprehensive and extensive analysis that brings together a diverse array of data from various sources, such as surveys, censuses, vital statistics, and health-related information^11^. These data are utilized to evaluate a wide range of health indicators. The Global Health Data Exchange provides interactive access to this repository, enabling researchers and policymakers to explore and analyze the findings. Leveraging this database, the present study quantified the burden of smoking-attributable neurodegenerative diseases at multiple levels: globally, across 5 sociodemographic index (SDI) regions, 21 regions, and 204 countries and territories. The study covered data from 1990 to 2021. This secondary analysis utilized the Global Burden of Disease (GBD) database and applied the comparative risk assessment (CRA) framework. The framework estimates the disease burden attributable to a risk factor (e.g. active smoking) by calculating the proportional reduction in a specific outcome (e.g. ischemic stroke) that would occur if exposure were reduced to the theoretical minimum risk exposure level (TMREL). Specifically, data on mortality and disability-adjusted life years (DALYs) for dementia (ICD-10 codes: F00-F03, G30-G31) and multiple sclerosis (ICD-10 code: G35), along with their corresponding 95% uncertainty intervals (UIs), were extracted from the GBD 2021. The UIs reported in GBD quantify overall uncertainty from multiple sources, as opposed to confidence intervals which primarily reflect sampling variability alone. This study evaluated mortality and DALYs rates using the age-standardized rate (ASR) and its corresponding 95% uncertainty interval (UI), with the 95% UI being used to reflect the reliability of the data and the robustness of the model. The ASR denotes an estimate per 100000 people, and the use of the ASR can facilitate scientific comparisons between populations, despite differences in age distribution and population size across populations, thereby improving the precision of population comparisons. These rates are derived from the following formula:


$$\text{ASR}=\frac{{\text{Σ}}_{i=1}^{A}a_{i}w_{i}}{{\text{Σ}}_{i=1}^{A}w_{i}}\times 100000$$

where A denotes the number of age groups, i denotes the ith age group, a_i_ denotes the rate to be standardized, and w_i_ denotes the number of standardized populations of the same age. Estimated annual percentage change (EAPC) to analyze the trend over time was calculated from the regression model: Y is the natural logarithm of the ASR, α is the intercept, β is a variable that determines the positive or negative trend of the ASR, X denotes the calendar year, and ε is the error term. the EAPC and its 95% confidence interval (CI) are also derived from the model and calculated as EAPC=100×[exp (β)-1]. To gain deeper insights into the age and sex distribution trends of the target population, this study analyzed both mortality and DALYs, along with their ASRs. This analysis aimed to identify sex-specific patterns of the disease burden across different age groups and to determine the peak age of burden for each sex.

### Joinpoint regression

To assess the AAPC (%), the Joinpoint Regression Program (version 5.1.0.0) was used to calculate the AAPC of the age-standardized mortality rate (ASMR) and age-standardized DALY rate (ASDR), with corresponding 95% confidence intervals (CIs). Joinpoint is statistical software for the analysis of trends using joinpoint models; it utilizes trend data and fits the simplest joinpoint model allowed by the data^12^. The minimum and maximum number of joinpoints are user-defined.

### Sociodemographic index

This study analyzes the association between neurodegenerative diseases attributable to smoking and the level of socio-economic development of countries or regions using the SDI^13^. The SDI integrates factors such as the average years of schooling of the population aged ≥15 years, the total fertility rate of women aged <25 years, and the lagged effect of income distribution, and classifies countries and regions into five levels of development (low, medium-low, medium, medium-high, and high), with a range of values from 0 to 1. The specific thresholds for the classification are: low SDI (<0.45), low-middle SDI (≥0.45 and <0.61), middle SDI (≥0.61 and <0.75), high-middle SDI (≥0.75 and <0.90), and high SDI (≥0.90). In addition, the study analyzed the burden of disease at the global, regional, and national levels, explored differences in disease among different age groups, and described trends over time. To assess the association between ASR and SDI, we used Spearman correlation analysis including R-index and p-value.

### Bayesian age-period-cohort (BAPC) analysis

The BAPC model is a method used in epidemiology and biostatistics to analyze the relationship between incidence rates and temporal trends^14^. It utilizes sample data and prior information to derive unique parameter estimates, producing robust and reliable results. Using the BAPC package within R software (version 4.4.2), we projected incidence trends from 2022 to 2035. These projections can inform public health policy development and prevention strategies.

### Health inequality analysis

This study employed the slope index of inequality (SII) and the concentration index (CoI), as defined by the World Health Organization (WHO), to measure absolute and relative inequalities in disease burden^15^. These indices quantify distributional disparities of diseases across specific contexts, providing a comprehensive systematic assessment across 204 countries and territories. The SII was calculated by regressing DALYs rates against the fractional rank of the cumulative population distribution ordered by SDI. We compared data from 204 countries and territories between 1990 and 2021, to analyze changes in health inequalities. We applied a robust regression model (RLM) instead of an ordinary linear model (LM) in our health inequality analysis, to better control for bias and heterogeneity.The robust regression model minimizes bias induced by data heterogeneity or extreme values, reduces sensitivity to outliers, and yields a more accurate representation of health inequalities. Additionally, the concentration index was computed by integrating the area under the Lorenz curve, relating the cumulative proportion of DALYs to the cumulative proportion of the population ranked by SDI.

### Frontier analysis

To assess the relationship between smoking-attributable dementia and multiple sclerosis burden and sociodemographic development levels, we employed frontier analysis to construct ASDR-based frontier models using the SDI^16^. This advanced statistical approach accounts for the nonlinear relationship between SDI and disease burden while capturing multidimensional drivers of smoking-attributable dementia and multiple sclerosis, unlike conventional regression models that describe variable associations or predict outcomes.The frontier analysis establishes a benchmark for optimal performance, by identifying the theoretical minimum achievable ASDR for each country or territory at its current development level. This methodology allows to identify actionable areas for improvement by quantifying the gap between a location’s observed burden and its potential minimum burden. We applied locally weighted polynomial regression (Loess) with varying smoothing spans (0.3, 0.4, 0.5) to generate smoothed frontier lines that capture the nonlinear SDI–ASDR relationship. To ensure analytical robustness, we performed 1000 bootstrap iterations and computed the mean ASDR for each SDI value. By measuring the vertical distance (i.e. the effectiveness gap) between each location’s 2021 ASDR and the frontier line, we quantified the potential for improvement.

### Decomposition analysis

The Das Gupta decomposition method was utilized to decompose changes in smoking-attributable dementia and multiple sclerosis burden from 1990 to 2021, isolating the contributions of population aging, population growth, and epidemiological change^17^. This approach enabled the partitioning of the overall change in burden into these key components, providing clearer insights into how demographic and epidemiological transitions shaped trends over time. By dissecting these trends, we gained clearer insights into the underlying drivers of global burden changes in congenital birth defects.

All p-values were two-sided, and p<0.05 was considered statistically significant. All statistical analyses and data visualizations were performed using R (version 4.5.1) and JD_GBDR (V2.37, JingdingMedical Technology Co. Ltd.).

## RESULTS

### Globe, region, and national level

**Figure 1:**
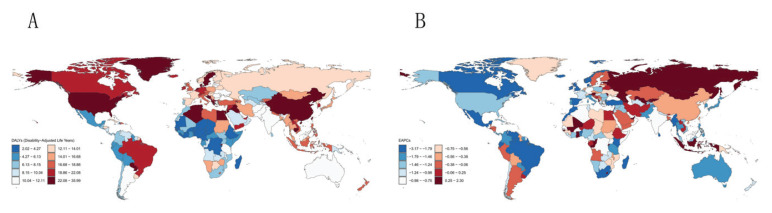
Global burden in 2021: A) The age-standardized disability adjusted life years of Alzheimer's disease and other dementias; B) Estimated annual percentage change in age-standardised disability-adjusted life years for Alzheimer's disease and other dementias

**Figure 2:**
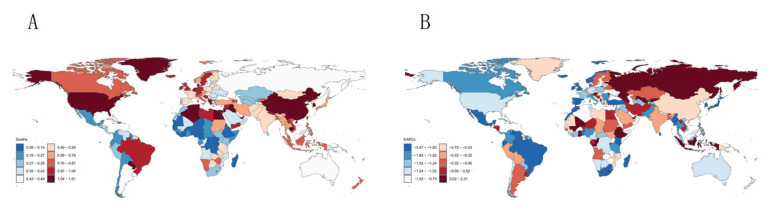
Global burden in 2021: A) The age-standardized mortality of Alzheimer's disease and other dementias; B) Estimated annual percentage change in age-standardized mortality for Alzheimer's disease and other dementias

Globally, the number of dementia deaths attributable to smoking rose to 67175.82, exceeding twice the 1990 value. DALYs increased from 794915.20 (95% UI: 344377.51–1839709.42) to 1533213.54 (95% UI: 662722.71–3496419.97) (Supplementary file Table S1). These findings underscore the substantial and growing burden of dementia associated with smoking. All five SDI regions exhibited increases in both smoking-attributable dementia mortality and DALYs, albeit to varying degrees. Among the 21 regions analyzed, Southern Sub-Saharan Africa was the only one to demonstrate a decline in both deaths and DALYs. Following age-standardization, an upward trend in smoking-attributable dementia burden was observed in only two of the 21 regions: Central Asia and Eastern Europe. The remaining regions all exhibited declining trends. This pattern highlights the likely contribution of demographic factors, notably population aging, to these findings. At the national level in 2021, China demonstrated the highest crude number of smoking-attributable dementia mortality (24897.31; 95% UI: 5866.82–70648.41) and DALYs (602501.08; 95% UI: 257945.38–1379582.96) (Figures 1 and 2). Following age standardization, Albania exhibited the highest ASMR, while Lebanon demonstrated the highest ASDR. Both regions exhibited an upward trend.

**Figure 3:**
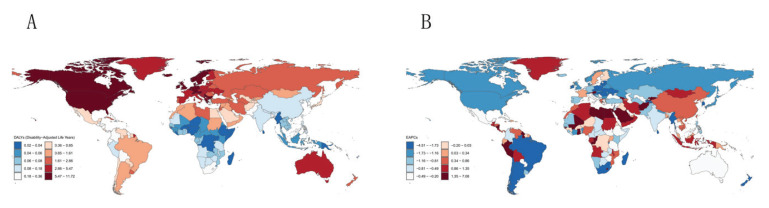
Global burden in 2021: A) The age-standardized disability adjusted life years of multiple sclerosis; B) Estimated annual percentage change in age-standardised disability-adjusted life years for multiple sclerosis

**Figure 4:**
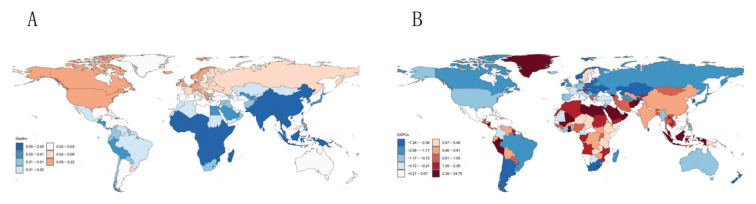
Global burden in 2021: A) The age-standardized mortality of multiple sclerosis; B) Estimated annual percentage change in age-standardized mortality for multiple sclerosis

Globally, the DALYs attributable to smoking-induced MS reached 111100 in 2021, highlighting the urgency of addressing this public health issue (Supplementary file Table S2). Epidemiological analysis reveals pronounced geographical disparities, with Western Europe and high-income North America bearing the highest disease burden. Particularly striking is the most substantial growth observed in high-income North America. In contrast, Oceania and Western Sub-Saharan Africa accounted for a comparatively smaller proportion of DALYs. At the national level, the United States exhibited the highest mortality burden from smoking-associated MS in 2021. Germany and the United Kingdom followed, though both countries demonstrated marked declines since 1990. The DALYs mirrored those observed in mortality rates, with pronounced heterogeneity across nations. Following age standardization, global ASMR and ASDR for smoking-related MS exhibited declines compared to 1990 levels. However, analysis of global trends stratified by the SDI revealed divergent patterns. Low- and low-middle-SDI regions demonstrated an upward trajectory in both ASMR and ASDR, contrasting with the global decline. Conversely, middle, high-middle, and high SDI regions aligned with the global downward trend. Among the 21 regions, European territories – Western Europe, Eastern Europe, Central Europe – alongside high-income North America emerged as critical burden hotspots, collectively accounting for substantial mortality counts and disproportionate DALYs. At the national level, the United States, Canada, and selected European countries remain the predominant contributors to the smoking-attributable MS burden (Figures 3 and 4).

### SDI analysis of MS burden attributable to smoking

For dementia, the analysis revealed declining trends across all SDI regions (Supplementary file Figure S1). However, while high-SDI regions exhibited the highest mortality and DALY rates in 1990, high-middle SDI regions emerged as the highest by 2021. High-SDI regions demonstrated the most substantial decline. Similarly, the disease burden of MS attributable to smoking had shown an overall decline in all SDI regions (Supplementary file Figure S1). However, significant disparities persisted, with high-SDI regions consistently remaining the primary contributors to the disease burden. High-middle SDI and middle SDI regions exhibited relatively comparable levels of disease burden.

### Age and gender pattern

Across all age groups, males consistently exhibited higher smoking-attributable dementia ASMR and ASDR than females (Supplementary file Figure S2). ASMR peaked in the age group 80–84 years in males, while females demonstrated a slightly later peak occurring at 85–89 years. Analysis of crude rates demonstrated that both the counts of mortality and DALYs are predominantly concentrated in individuals aged ≥90 years. However, smoking-related MS predominantly affects middle-aged and older populations, demonstrating a striking divergence from dementia (Supplementary file Figure S2). Analysis of crude rates demonstrated that mortality peaks for both genders occurred within the 60–64 years age group. Similarly, the peak DALYs for males emerged in the 60–64 years cohort, whereas females exhibited a marginally distinct pattern with peak DALYs observed between 55–60 years, though with minimal overall differences. Following age standardization, the peak burden for both sexes shifted to the 55–59 years age bracket. Notably, ASMR and ASDR consistently demonstrated elevated values in males compared to females across nearly all age groups.

### Temporal join-point analysis

Based on joinpoint regression analysis, the age-standardized dementia death rate (ASDR; AAPC= -0.162; 95% CI: -0.166 – -0.159) and age-standardized mortality rate (ASMR; AAPC= -0.008; 95% CI: -0.008 – -0.008) exhibited overall downward trends from 1990 to 2021 (Supplementary file Figure S3 and Table S3). The temporal inflection points for ASDR and ASMR were largely similar. Both rates demonstrated an accelerated decline beginning in 1995, followed by a brief upward trend in 2019; however, the p-values indicated that these inflection points were not statistically significant. Two additional inflection points occurred in close proximity, specifically in 2007 and 2006 for ASDR and ASMR, respectively. The period of the most rapid decline in ASDR occurred between 1995 and 2006 (APC= -1.202; 95% CI: -1.240 – -1.164; p<0.001). Similarly, the most rapid decline in ASMR was observed from 1995 to 2007 (APC= -1.241; 95% CI: -1.269 – -1.214; p<0.001).

Similarly, both the ASDR (AAPC= -0.035; 95% CI: -0.035 – -0.034) and ASMR (AAPC= -0.001; 95% CI: -0.001 – -0.001) of MS demonstrated an overall downward trend from 1990 to 2021 (Supplementary file Figure S3 and Table S3). The ASMR exhibited varying declining trends across different periods, with the most pronounced decrease observed from 2003 to 2013 (APC= -3.067; 95% CI: -3.541 – -2.590; p<0.001). The decline in ASDR showed less variability, though its steepest reduction occurred between 2003 and 2012 (APC= -2.612; 95% CI: -2.736 – -2.488; p<0.001). Notably, both ASDR and ASMR experienced a significant acceleration in their downward trends starting in 2003.

### Future trend predictions

Our study identified smoking as a persistent global risk factor for both dementia and multiple sclerosis (Supplementary file Figure S4). Based on these findings, we extended the prediction model to estimate future trends in smoking-attributable incidence and mortality of dementia and multiple sclerosis through 2035. Although crude mortality rates and DALYs showed increases compared to 1990 levels – consistent with projected global population growth – age-standardized incidence and mortality rates for both conditions demonstrated significant declining trends.

### Additional analysis

To further investigate this relationship, we conducted a health inequality analysis (Supplementary file Figure S5 and Table S4). The SII indicated that health inequalities in smoking-attributable dementia and MS persisted in 2021 and exhibited a positive correlation with the SDI. However, compared to 1990 levels, these inequalities had decreased to varying extents. Notably, the SII for the ASDR of smoking-attributable dementia was relatively high at 8.60 (95% CI: 6.10–11.11). The CI revealed that inequalities in smoking-attributable MS were less pronounced than those for smoking-attributable dementia in both ASDR (reliable change index, RCI=0.56; 95% CI: 0.50–0.61) and ASMR (RCI=0.58; 95% CI: 0.51–0.64).

Frontier analysis indicates that several countries remain at the forefront of disease burden (Supplementary file Figure S6). Regarding smoking-attributable dementia, Lebanon exhibits the highest disease burden as measured by DALYs, with a continuing upward trend. China and Albania follow closely behind, ranking second and third, respectively. However, China demonstrates a declining trend in disease burden. From the mortality perspective, Lebanon remains at the forefront of disease burden for smoking-attributable dementia. Rwanda and Egypt follow closely behind, ranking second and third, respectively, with all three countries exhibiting upward trends in burden. For smoking-attributable MS, Denmark exhibits the highest disease burden in both mortality and DALYs, with Albania ranking second.

Decomposition analysis revealed that the global ASDR and ASMR for smoking-attributable dementia were primarily driven by demographic factors (Supplementary file Figure S7). The contributions of population aging and epidemiological changes were also substantial. Conversely, for smoking-attributable MS, the influence of population aging was minimal. Changes were predominantly driven by epidemiological shifts and demographic factors, with the latter being the principal driver. These findings collectively indicate that demographic factors are a significant driver of smoking-attributable neurodegenerative diseases.

## DISCUSSION

In 2021, data revealed that the crude death count and DALYs count due to smoking-induced dementia were higher than those due to multiple sclerosis, indicating a comparatively more severe disease burden. Decomposition analysis indicates that population growth was a major driver of these results. Since 1990, the ASMR and ASDR for dementia have both declined. In contrast, multiple sclerosis exhibited a substantially greater decline in both ASMR and ASDR over the same period. Substantial variations in smoking-attributable neurological disease burden were observed across age groups, WHO regions, and SDI regions. According to the GBD database, dementia and multiple sclerosis constituted the predominant neurodegenerative disorders attributable to smoking exposure. Health inequality persists, with some countries still at the forefront of disease burden.

Over recent decades, the global ASDR and ASMR for smoking-attributable neurodegenerative diseases have exhibited declining trends. BAPC projection models further indicate continued reductions in the global burden of dementia and MS attributable to smoking in 2023. At the SDI level, dementia demonstrates comparatively favorable outcomes: all five SDI regions show consistent declines in both ASDR and ASMR. Conversely, low- and low-middle SDI regions exhibit upward trajectories in ASDR and ASMR for MS, with particularly substantial rises observed in low-SDI regions. These findings underscore the need for enhanced global tobacco control initiatives. From the perspective of 21 regions, they are all concentrated in Asia and Europe. This may be closely related to the promotion of the tobacco industry by developed economies and prevailing lifestyles. At the national level, China recorded the highest number of deaths and DALYs attributable to smoking-induced dementia, while the United States had the highest values for deaths and DALYs linked to smoking-related multiple sclerosis. However, following age standardization, the results differed, indirectly highlighting the significant influence of age structure on disease mortality and DALYs in this context. Frontier analysis ultimately revealed that Lebanon bears the highest burden of dementia attributable to smoking, whereas Denmark leads in the burden of smoking-related multiple sclerosis. Age and gender patterns further indicated that males consistently outnumbered females across all age groups in smoking-attributable cases. Additionally, multiple sclerosis affects younger age groups compared to dementia in terms of both mortality and DALYs. These findings suggest that countries should develop tailored tobacco control policies based on their specific national contexts. Furthermore, disease prevention for specific smoking populations requires targeted approaches, considering each nation’s unique demographic structure in terms of age and gender.

Tobacco control has gained significant traction globally. Countries including the United Kingdom^18^, China^19^, Spain^20^, Bolivia^21^, and the Netherlands^22^ have strengthened tobacco control policies, implementing evidence-based strategies such as tobacco taxation, plain packaging with health warnings, and comprehensive bans on advertising, promotion, and sponsorship. Concurrently, significant advancements were observed in regional tobacco control frameworks. Nevertheless, substantial implementation gaps persist across multiple geographical regions, particularly in Europe^23^, Latin America^24^, and Africa^25^. Continuous monitoring and reporting of progress for each intervention facilitates the assessment of policy effectiveness, identification of persisting gaps, and strategic planning for future actions^26^.

Smoking is associated with significant effects on the nervous system through multiple pathways^27^, impacting the prognosis of both dementia and multiple sclerosis. Smoking is associated with the induction of cerebral oxidative stress, which may trigger the deposition of Aβ peptides and hyperphosphorylated tau proteins^28^. This cascade may contribute to neurodegeneration, potentially elevating the risk of AD. Furthermore, smoking is an established risk factor for cerebral infarction^29^. Damage to cerebrovascular endothelial cells and recurrent cerebral small vessel injury have been observed following nicotine exposure. Such vascular pathology is widely recognized as a key mechanism contributing to vascular dementia (VaD), a major dementia subtype. Evidence suggests that the impact of smoking on MS may involve several mechanisms, including disruption of the blood–brain barrier, inflammation and immune modulation, and neurotoxicity^30,31^. Studies have found that compounds present in tobacco impair the viability of blood–brain barrier cells^32^. Furthermore, tobacco smoke serves as a source of hydrogen cyanide and its metabolites (such as thiocyanate), which can induce demyelination of white matter^33,34^. Significantly, smoking alters the levels of interleukin-1 and interleukin-6 in humans^35^. These mediators can enter the central nervous system and interact with other inflammatory markers, affecting autoimmune responses, thereby influencing the progression of MS. Therefore, it is imperative to implement further tobacco control initiatives.

### Strengths and limitations

This study yields findings of scientific importance. Firstly, the detailed analysis of historical global data facilitates earlier screening and enables age-targeted early intervention, thereby advancing the implementation of early-stage prevention for neurodegenerative diseases among smokers. Furthermore, it provides evidence-based justification for sex-specific prevention strategies within this context from a global perspective. Our results identify specific regions and nations as high-risk zones, offering guidance for targeted resource allocation. This work additionally serves as an early warning system for healthcare system deficiencies in managing neurological diseases, particularly highlighting that smoking-attributable dementia exhibits significant health inequities.

This study systematically analyzed the epidemiological characteristics of dementia and multiple sclerosis in the context of smoking from 1990 to 2021 and projected future disease trends through 2035. However, several limitations warrant acknowledgment. Firstly, despite the Global Burden of Disease study’s integration of multinational data, heterogeneity in data quality exists. Variations in reporting standards, diagnostic criteria, and data collection methodologies may introduce potential biases in the findings. Secondly, GBD estimates primarily derive from population-level statistics, which may obscure individual-level variations. This limitation constrains more granular analysis of specific disease burden determinants. Thirdly, the observational and ecological nature of much of the underlying data limits causal inference. The associations reported between smoking exposure and disease burden, as well as the correlations with sociodemographic index and health inequality metrics, do not constitute proof of causality and may be subject to residual confounding from unmeasured or inadequately adjusted factors. Fourthly, the analyses involving multiple comparisons across different regions, age groups, and time points increase the risk of Type I error, where some statistically significant findings might arise by chance rather than representing true associations. Fifthly, while the predictive models employed for disease forecasting generally provide reasonable estimates, they may fail to capture region-specific contextual factors. Disparities in healthcare access, socioeconomic conditions, and cultural backgrounds across nations could lead to differential expressions of disease burden, even under comparable exposure levels. Finally, due to data constraints, this study could not conduct detailed analyses of dementia subtypes. This limitation impedes the ability to inform subtype-specific clinical interventions. Future validation using updated GBD iterations is warranted. Consequently, more refined analyses targeting specific regions or populations will require localized data and field studies. Nevertheless, this research provides valuable insights for healthcare decision-making and public health policy formulation.

## CONCLUSIONS

Although ASMR and ASDR for smoking-attributable dementia and multiple sclerosis declined between 1990 and 2019, the absolute number of deaths and DALYs continued to rise. The persistent global disease risk warrants sustained vigilance. Significant heterogeneity exists across age groups, genders, regions, and nations, with pronounced health disparities enduring. These results emphasize the necessity for comprehensive smoking cessation campaigns and targeted interventions in regions with high dementia/MS burden and high smoking prevalence.

## Supplementary Material



## Data Availability

The data supporting this research are available from the authors on reasonable request.
