# Supplementary material for: Global, regional and national burden of neurodegenerative diseases attributable to smoking: A descriptive study based on the Global Burden of Disease Study 2021
Source: Tob Induc Dis. 2026 Mar 9;24:10.18332/tid/217009. doi: 10.18332/tid/217009 (PMC12974404; doi:10.18332/tid/217009)
Supplement: Supplementary file 1 [file TID-24-33-s1.pdf]

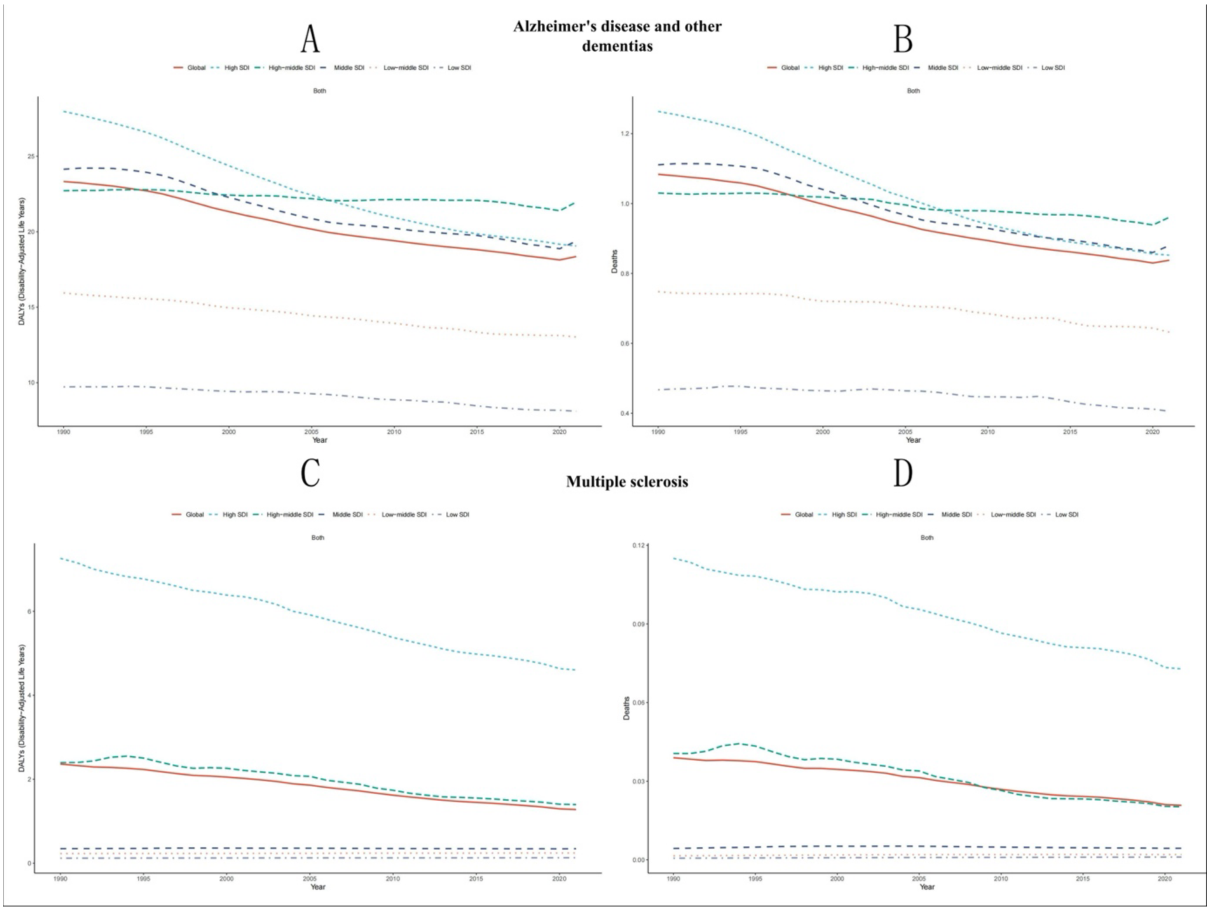

Figure S1. Correlation between ASR of neurodegenerative diseases attributable to smoking and SDI in 2021. (A) The age-standardized DALYs of Alzheimer's disease and other dementias;(B) The age-standardized mortality of Alzheimer's disease and other dementias;(C) The age-standardized DALYs of Multiple sclerosis; (D) The age-standardized mortality of Multiple sclerosis. The Y-axis represents the age-standardised rate, while the X-axis denotes the year. Different curves represent distinct Sociodemographic Index regions.

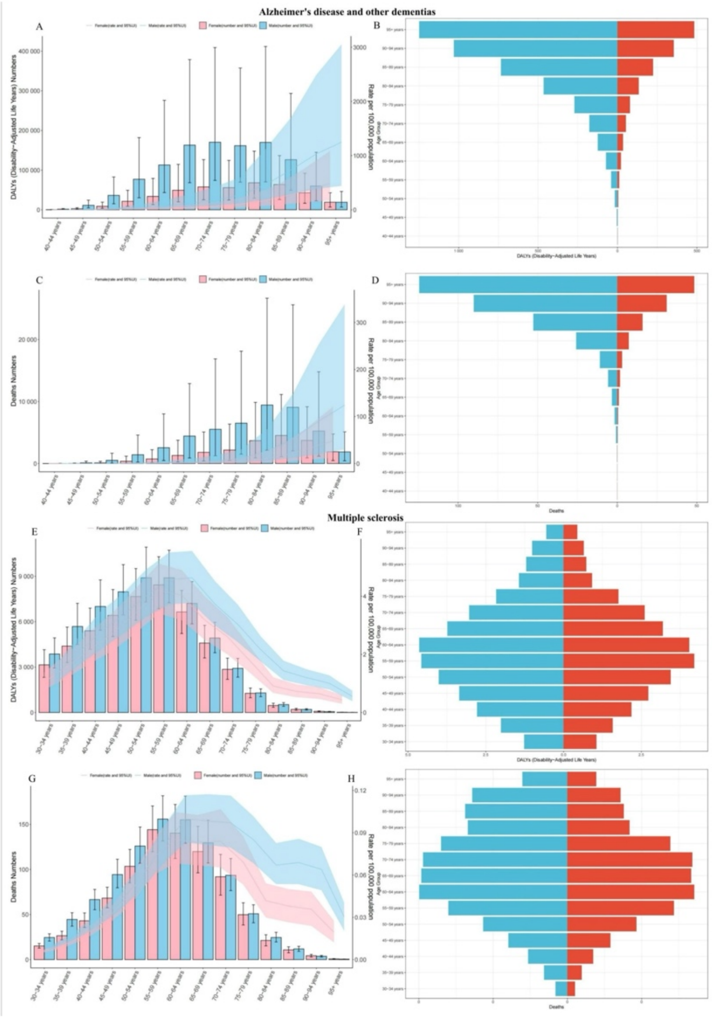

Figure S2. Distribution and trends (by Age and Sex) of the burden in 2021. (A, B) The DALYs of Alzheimer's disease and other dementias; (C, D) The mortality of Alzheimer's disease and other dementias; (E, F) The DALYs of Multiple sclerosis; (G, H) The mortality of Multiple sclerosis. Figure A, C, E, G: Bar represents number and the trend line represents rate. The shaded areas are 95% CI. Figure B, D, F, H: The red bar chart represents the number of females, while the blue bar chart represents the number of males.

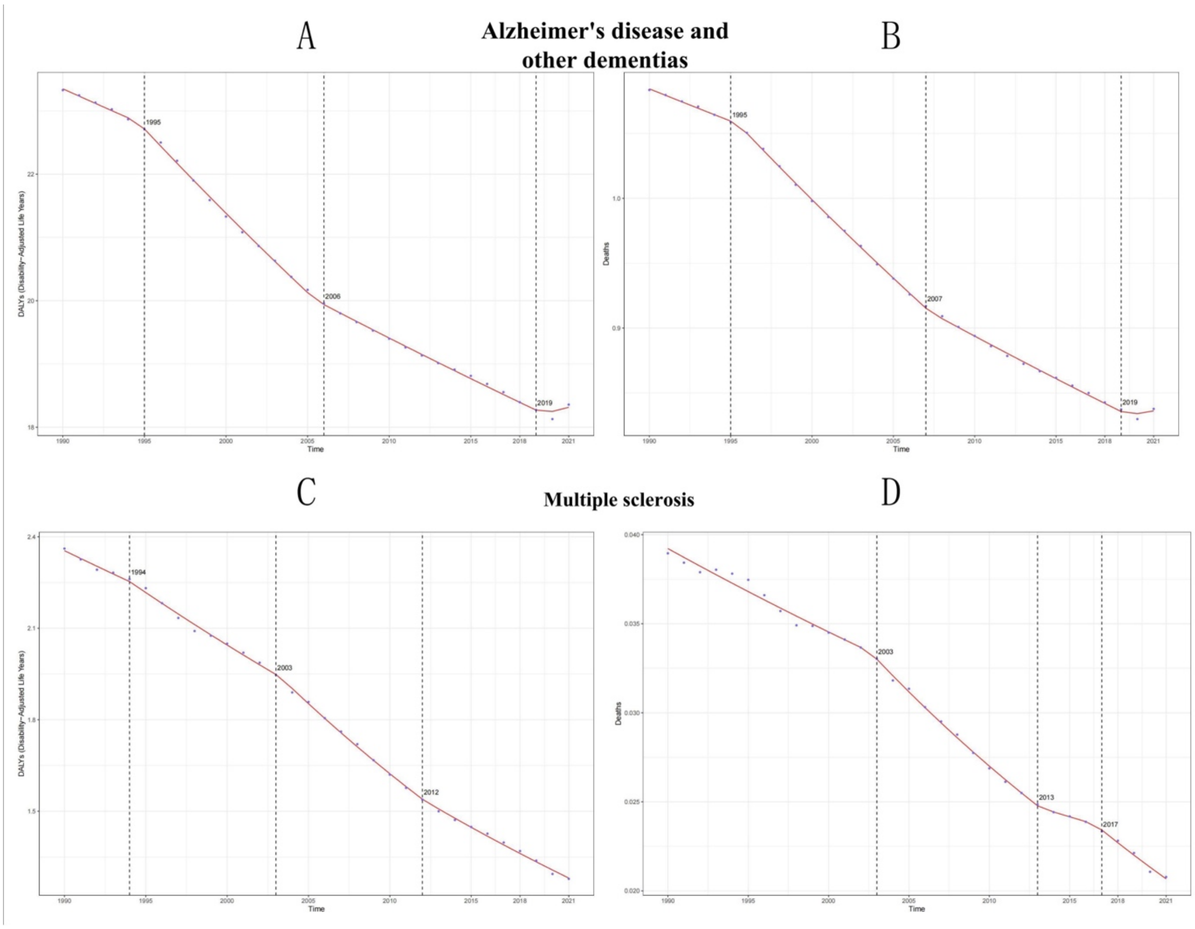

Figure S3. Join-point Regression Analysis of temporal trends in the burden of neurodegenerative diseases attributable to smoking from 1990 to 2021. (A) The age-standardized DALYs of Alzheimer's disease and other dementias;(B) The age-standardized mortality of Alzheimer's disease and other dementias;(C) The age-standardized DALYs of Multiple sclerosis; (D) The age-standardized mortality of Multiple sclerosis. The Y-axis represents the age-standardised rate, while the X-axis denotes the year.

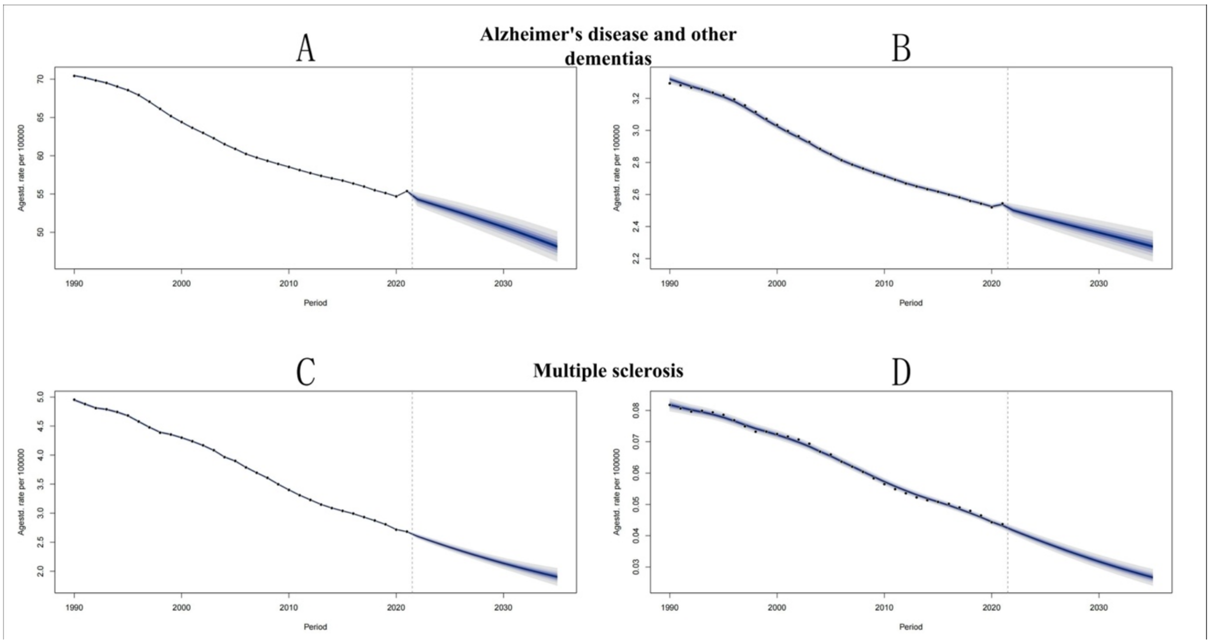

Figure S4. Trends in the Burden of neurodegenerative diseases attributable to smoking: Observed Rates (1990-2021) and Predicted Rates (2022-2035). (A) The age-standardized DALYs of Alzheimer's disease and other dementias;(B) The age-standardized mortality of Alzheimer's disease and other dementias;(C) The age-standardized DALYs of Multiple sclerosis; (D) The age-standardized mortality of Multiple sclerosis. The Y-axis represents the age-standardised rate, while the X-axis denotes the year.

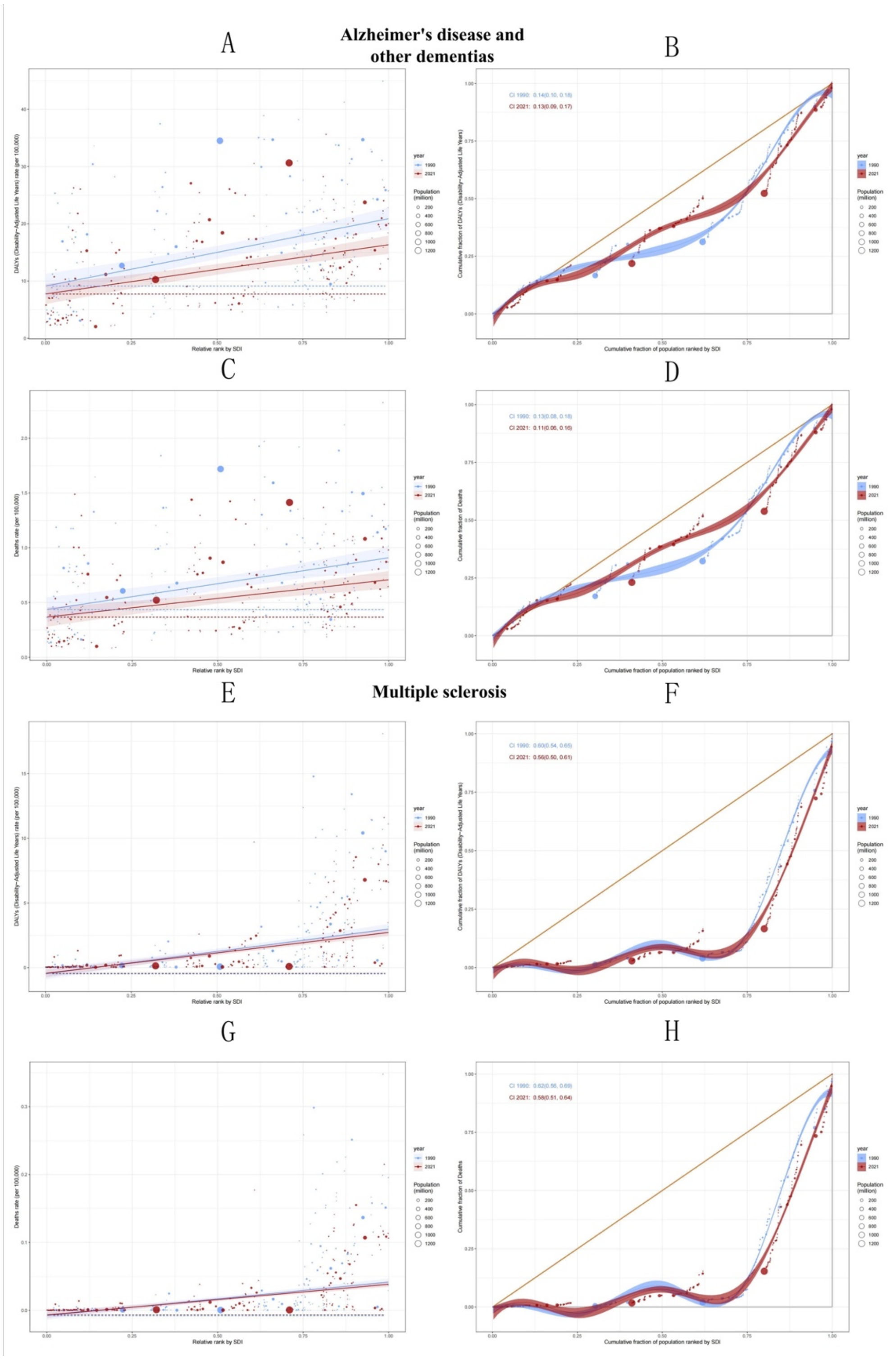

Figure S5. SDI-related health inequality regression curves and concentration curves for the global burden of Decubitus Ulcers, 1990 and 2021. The health inequality regression curve on the left and the concentration curve on the right. (A, B) The age-standardized DALYs of Alzheimer's disease and other dementias;(C, D) The age-standardized mortality of Alzheimer's disease and other dementias;(E, F) The age-standardized DALYs of Multiple sclerosis; (G, H) The age-standardized mortality of Multiple sclerosis. In the absolute inequality analysis, the Y-axis represents age-standardised rates, while the X-axis denotes sociodemographic index values. In the relative inequality analysis, the Y-axis represents the cumulative fraction for age-standardised rates, while the X-axis represents the sociodemographic index values.

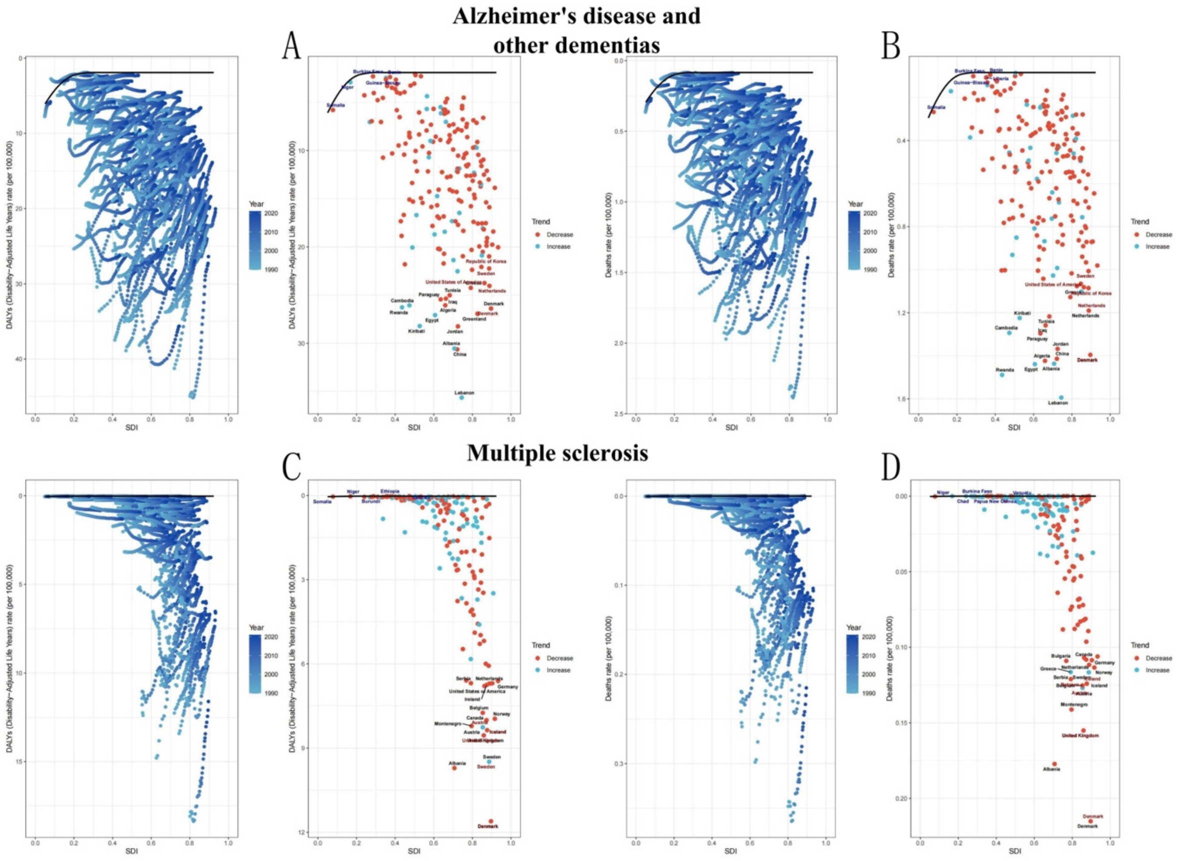

Figure S6. Frontier analysis involving SDI and neurodegenerative diseases attributable to smoking burden in 2021. (A) The age-standardized DALYs of Alzheimer's disease and other dementias;(B) The age-standardized mortality of Alzheimer's disease and other dementias;(C) The age-standardized DALYs of Multiple sclerosis; (D) The age-standardized mortality of Multiple sclerosis. The Y-axis represents age-standardised rates, while the X-axis denotes sociodemographic index values.

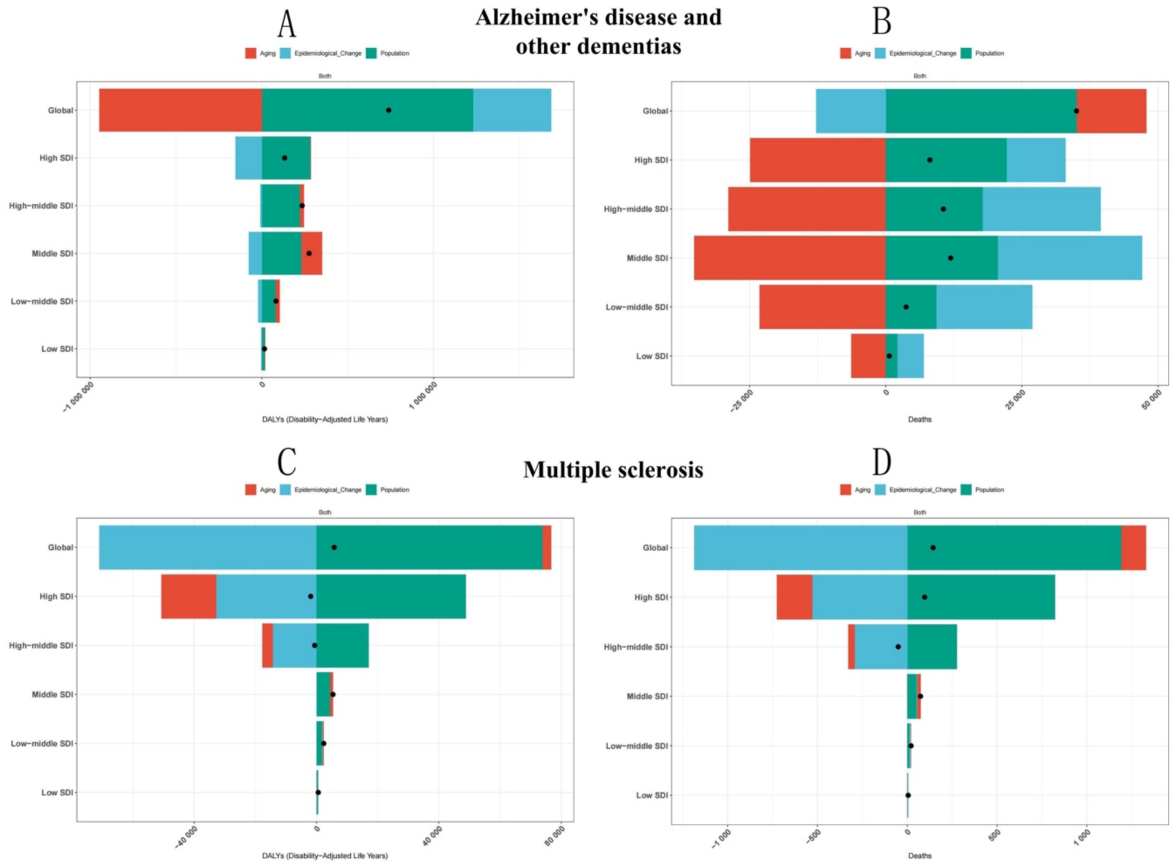

Figure S7. Key drivers of Decubitus Ulcers burden at global, SDI levels, 21 regions from 1990 to 2021: population growth, ageing, and epidemiological changes. The black dots represent the sum of contributions to changes in all three factors. (A) The age-standardized DALYs of Alzheimer's disease and other dementias;(B) The age-standardized mortality of Alzheimer's disease and other dementias;(C) The age-standardized DALYs of Multiple sclerosis; (D) The age-standardized mortality of Multiple sclerosis. The Y-axis represents different sociodemographic index regions, while the X-axis denotes the percentage share of each driving factor.

Table S1 The number of Mortality and DALYs of Alzheimer's disease and other dementias, along with corresponding ASR and temporal trends.

Characteristics	Mortality(95% uncertainty interval)					DALYs(95% uncertainty interval)				
	Cases,1990	Cases,2021	ASMR,1990	ASMR,2021	EAPC 1990-2021	Cases,1990	Cases,2021	ASDR,1990	ASDR,2021	EAPC 1990-2021
Global	32164.82(7445.51,89320.27)	67175.82(15694.65,184664.76)	1.08(0.25,3.03)	0.84(0.19,2.29)	-0.95(-0.99,-0.91)	794915.20(344377.51,1839709.42)	1533213.54(662722.71,3496419.97)	23.33(9.99,54.46)	18.36(7.90,42.07)	-0.88(-0.92,-0.83)
SDI level										
High SDI	13666.22(3193.36,38022.68)	21770.67(5160.07,60011.50)	1.26(0.29,3.55)	0.85(0.20,2.36)	-1.41(-1.47,-1.35)	310666.04(136518.80,716664.77)	442915.27(190964.64,1020743.64)	27.97(12.27,64.53)	19.05(8.33,43.29)	-1.34(-1.40,-1.28)
High-middle SDI	7880.68(1767.51,22010.20)	18462.72(4278.75,50254.29)	1.03(0.23,2.89)	0.96(0.22,2.60)	-0.31(-0.33,-0.28)	200798.18(84592.04,461915.08)	435095.67(191159.58,971668.74)	22.72(9.46,52.39)	21.96(9.60,49.15)	-0.17(-0.19,-0.14)
Low SDI	559.62(130.74,1625.67)	1216.04(286.56,3510.84)	0.47(0.11,1.36)	0.41(0.10,1.18)	-0.46(-0.54,-0.39)	15214.67(6575.47,36753.83)	30071.64(11977.78,72619.96)	9.72(4.09,23.07)	8.10(3.29,19.44)	-0.64(-0.70,-0.59)
Low-middle SDI	2733.65(625.62,7836.22)	6466.32(1472.81,18526.77)	0.75(0.17,2.14)	0.63(0.14,1.81)	-0.55(-0.59,-0.51)	71967.31(31552.49,171280.44)	153656.95(63420.52,366741.77)	15.95(6.82,37.64)	13.02(5.45,31.35)	-0.70(-0.72,-0.68)
Middle SDI	7295.54(1666.64,20480.30)	19214.62(4535.50,54275.94)	1.11(0.26,3.14)	0.88(0.21,2.45)	-0.95(-1.01,-0.89)	195492.62(83542.77,446430.43)	470384.96(201779.14,1086306.44)	24.14(10.01,56.67)	19.38(8.25,44.76)	-0.88(-0.94,-0.81)
GBD Region										
Andean Latin America	52.95(11.75,157.54)	141.21(30.55,416.57)	0.32(0.07,0.97)	0.26(0.06,0.75)	-0.85(-0.96,-0.74)	1261.62(530.20,2998.35)	3187.59(1352.82,7589.45)	7.05(2.96,16.84)	5.65(2.40,13.40)	-0.84(-0.93,-0.74)
Australasia	172.78(39.75,485.44)	337.16(80.47,936.95)	0.77(0.17,2.16)	0.54(0.13,1.50)	-1.10(-1.19,-1.01)	4412.13(1974.59,10040.35)	7015.67(3016.71,16579.78)	18.86(8.37,42.69)	12.11(5.19,28.39)	-1.40(-1.47,-1.34)
Caribbean	131.43(29.06,369.67)	257.80(56.66,749.27)	0.61(0.13,1.74)	0.46(0.10,1.35)	-1.05(-1.19,-0.91)	3466.03(1490.12,7883.97)	6059.50(2666.38,14005.37)	14.49(6.29,33.31)	11.08(4.89,25.64)	-1.06(-1.20,-0.93)
Central Asia	161.21(37.52,463.05)	299.97(68.86,876.93)	0.42(0.10,1.20)	0.49(0.11,1.41)	0.96(0.82,1.10)	4359.82(1950.11,10094.91)	8069.78(3529.27,18936.20)	10.16(4.53,23.61)	11.45(5.08,26.38)	0.74(0.59,0.88)
Central Europe	946.99(226.85,2674.38)	1349.60(309.19,3691.06)	0.76(0.18,2.21)	0.56(0.13,1.55)	-0.99(-1.08,-0.90)	26911.11(11851.07,61193.25)	33947.20(14976.68,77856.86)	19.07(8.36,43.19)	14.77(6.44,33.75)	-0.84(-0.90,-0.78)
Central Latin America	336.98(76.13,983.18)	655.18(147.47,1825.87)	0.55(0.12,1.61)	0.28(0.06,0.78)	-2.36(-2.48,-2.23)	8966.49(3991.71,20343.85)	16186.82(7134.06,35871.74)	12.89(5.72,29.41)	6.74(2.97,14.90)	-2.25(-2.37,-2.13)
Central Sub-Saharan Afr	31.09(6.81,93.53)	72.34(16.11,218.78)	0.27(0.06,0.79)	0.22(0.05,0.64)	-0.51(-0.81,-0.21)	964.29(390.86,2326.23)	2170.14(823.42,5189.26)	5.88(2.41,14.34)	5.05(1.92,12.35)	-0.39(-0.67,-0.10)
East Asia	8275.34(1857.68,22997.59)	25333.42(5966.93,71868.54)	1.67(0.37,4.69)	1.38(0.32,3.87)	-0.73(-0.81,-0.65)	218922.24(90301.58,508008.64)	611760.52(262060.15,1401979.10)	33.68(13.33,78.42)	29.95(12.67,68.10)	-0.48(-0.58,-0.39)
Eastern Europe	992.68(226.15,2831.49)	1647.44(377.64,4664.06)	0.42(0.09,1.21)	0.45(0.10,1.28)	0.39(0.12,0.67)	28706.08(12568.17,65937.25)	43177.68(18713.43,97784.86)	10.81(4.76,24.69)	11.92(5.23,26.87)	0.43(0.15,0.71)
Eastern Sub-Saharan Afr	181.32(42.84,534.72)	340.27(80.89,973.39)	0.48(0.11,1.37)	0.35(0.08,0.99)	-1.25(-1.35,-1.15)	4798.05(1952.78,11610.54)	8554.00(3336.71,21035.32)	9.54(3.88,22.72)	6.91(2.76,16.31)	-1.17(-1.27,-1.07)
High-income Asia Pacifi	1982.06(464.95,5542.01)	4737.75(1133.34,12872.60)	1.19(0.27,3.29)	0.72(0.17,1.97)	-1.77(-1.85,-1.70)	45858.15(19474.86,106212.34)	87433.84(36859.42,198890.33)	24.84(10.44,57.20)	15.81(6.77,36.48)	-1.57(-1.62,-1.51)
High-income North Ame	5456.76(1298.37,15099.10)	7774.30(1888.37,21272.08)	1.48(0.35,4.09)	1.05(0.25,2.89)	-1.25(-1.32,-1.18)	125526.66(55483.87,291114.14)	164283.74(72809.79,373190.34)	34.48(15.23,79.44)	23.32(10.39,52.46)	-1.40(-1.47,-1.33)
North Africa and Middle	1183.89(273.25,3295.12)	2663.53(622.54,7704.54)	1.12(0.25,3.26)	0.85(0.19,2.46)	-0.97(-1.06,-0.88)	30637.27(13260.93,69329.98)	66396.79(28430.20,151668.29)	23.87(10.23,54.52)	18.22(7.72,41.54)	-0.96(-1.02,-0.89)
Oceania	8.80(1.89,25.73)	19.88(4.45,59.73)	0.51(0.11,1.42)	0.41(0.09,1.17)	-0.86(-0.93,-0.79)	298.51(124.32,699.01)	661.09(279.48,1524.18)	12.78(5.38,29.87)	10.53(4.46,24.93)	-0.73(-0.79,-0.67)
South Asia	2127.59(473.42,6148.34)	5663.57(1272.37,16142.37)	0.67(0.15,1.93)	0.55(0.13,1.59)	-0.60(-0.68,-0.53)	55863.97(24134.04,134194.63)	129918.09(52973.46,310723.34)	13.94(5.84,32.94)	10.95(4.55,26.31	-0.84(-0.88,-0.81)
Southeast Asia	1509.73(343.90,4245.19)	3541.55(832.35,10246.37)	0.93(0.21,2.68)	0.75(0.17,2.19)	-0.94(-1.09,-0.80)	40981.23(18278.54,93081.41)	89722.29(38568.94,210580.36)	20.78(9.10,47.13)	16.17(6.85,37.31)	-0.99(-1.10,-0.89)
Southern Latin America	184.80(42.11,537.12)	350.80(81.93,1017.29)	0.44(0.10,1.28)	0.38(0.09,1.11)	-0.38(-0.50,-0.26)	5659.50(2542.55,12844.68)	9343.13(4054.71,20860.40)	12.59(5.64,28.39)	10.53(4.59,23.53)	-0.56(-0.67,-0.45)
Southern Sub-Saharan A	167.74(39.70,468.23)	151.08(33.56,449.98)	0.88(0.21,2.37)	0.37(0.08,1.14)	-2.90(-2.99,-2.80)	4086.68(1794.13,9195.33)	4103.91(1728.64,9565.99)	18.60(8.00,41.45)	8.43(3.50,19.63)	-2.65(-2.74,-2.56)
Tropical Latin America	1028.32(234.34,2945.45)	2219.71(532.19,6034.94)	1.61(0.36,4.56)	0.91(0.22,2.49)	-2.03(-2.22,-1.85)	26317.50(10754.81,62011.30)	52048.25(22656.24,122054.32)	34.90(14.37,82.59)	20.81(9.05,48.62)	-1.87(-2.06,-1.68)
Western Europe	7134.66(1658.91,20442.73)	9444.24(2168.98,26106.23)	1.21(0.28,3.41)	0.78(0.18,2.18)	-1.49(-1.53,-1.45)	154412.36(67714.32,351220.85)	184593.67(78898.94,420812.86)	25.78(11.25,59.30)	17.23(7.43,38.39)	-1.34(-1.38,-1.31)
Western Sub-Saharan Af	97.69(21.66,276.67)	175.02(40.18,511.36)	0.18(0.04,0.52)	0.14(0.03,0.41)	-0.82(-0.86,-0.77)	2505.52(1013.64,5965.13)	4579.83(1793.79,11406.07)	3.72(1.48,8.68)	2.95(1.15,7.14)	-0.74(-0.76,-0.72)
National level										
Afghanistan	23.75(5.15,67.16)	28.78(5.90,81.42)	0.56(0.12,1.59)	0.54(0.11,1.49)	0.17(-0.08,0.42)	591.00(227.82,1428.16)	706.00(288.59,1676.36)	11.22(4.31,26.64)	10.74(4.24,25.32)	0.12(-0.14,0.38)
Albania	19.43(4.50,56.81)	57.56(12.68,167.54)	1.27(0.30,3.73)	1.44(0.31,4.20)	0.61(0.52,0.70)	471.34(206.75,1085.47)	1312.80(561.74,2977.56)	27.61(11.58,64.74)	30.54(12.98,69.92)	0.52(0.45,0.59)
Algeria	101.52(23.21,297.02)	289.49(59.35,835.28)	1.93(0.41,6.01)	1.42(0.30,4.15)	-0.81(-1.05,-0.57)	2673.59(1144.10,6435.66)	6708.70(2714.31,15287.43)	35.04(14.41,86.04)	26.05(10.06,60.20)	-0.88(-1.05,-0.70)
American Samoa	0.08(0.02,0.22)	0.18(0.04,0.53)	0.57(0.13,1.60)	0.49(0.11,1.45)	-0.54(-0.62,-0.47)	2.51(1.04,5.65)	5.32(2.20,12.11)	13.81(5.72,30.79)	12.21(5.10,28.79)	-0.46(-0.52,-0.40)
Andorra	0.48(0.11,1.36)	1.43(0.33,3.81)	1.07(0.24,2.91)	0.80(0.19,2.09)	-1.02(-1.07,-0.98)	12.01(5.05,27.85)	28.99(12.39,61.62)	23.31(9.58,53.24)	17.53(7.56,36.70)	-0.98(-1.03,-0.94)
Angola	8.84(1.96,25.99)	27.35(5.86,78.60)	0.45(0.10,1.28)	0.43(0.09,1.21)	-0.05(-0.28,0.19)	273.06(110.72,666.22)	808.52(303.06,1894.06)	10.05(4.06,24.55)	9.28(3.45,21.84)	-0.13(-0.35,0.10)
Antigua and Barbuda	0.17(0.04,0.49)	0.24(0.05,0.69)	0.29(0.07,0.84)	0.27(0.06,0.78)	-0.33(-0.38,-0.28)	4.14(1.84,9.23)	6.74(2.85,15.40)	7.34(3.31,16.68)	6.77(2.87,15.30)	-0.32(-0.38,-0.26)
Argentina	140.91(31.72,410.01)	255.42(58.12,734.10)	0.48(0.11,1.38)	0.44(0.10,1.26)	-0.17(-0.30,-0.04)	4295.14(1925.88,9775.00)	6748.98(2908.06,15233.14)	13.63(6.09,30.70)	11.88(5.15,26.74)	-0.37(-0.49,-0.26)
Armenia	16.86(3.87,46.25)	44.09(10.28,126.92)	0.82(0.19,2.27)	1.03(0.24,2.95)	1.18(1.00,1.37)	426.65(185.65,971.18)	943.73(395.37,2153.18)	18.10(7.82,40.56)	21.37(8.97,48.36)	0.91(0.78,1.04)
Australia	134.03(30.91,374.74)	258.87(60.24,713.92)	0.72(0.16,2.00)	0.49(0.11,1.36)	-1.19(-1.32,-1.05)	3476.69(1543.77,7899.30)	5419.43(2306.43,12741.36)	17.83(7.89,40.22)	11.13(4.75,26.10)	-1.49(-1.58,-1.39)
Austria	78.36(17.85,224.84)	168.75(37.69,467.34)	0.64(0.14,1.83)	0.76(0.17,2.07)	0.70(0.38,1.03)	1873.22(803.83,4385.10)	3587.93(1534.84,7913.81)	15.32(6.64,35.91)	18.07(7.80,39.91)	0.64(0.37,0.92)
Azerbaijan	20.74(4.62,59.67)	53.78(12.33,159.11)	0.51(0.11,1.49)	0.76(0.17,2.21)	1.82(1.61,2.04)	572.41(244.53,1322.39)	1439.35(636.15,3259.94)	12.62(5.29,28.70)	17.32(7.62,39.58)	1.40(1.17,1.63)
Bahamas	0.35(0.08,1.00)	0.93(0.19,2.62)	0.28(0.06,0.83)	0.28(0.06,0.81)	0.16(0.09,0.24)	9.60(4.14,22.01)	24.85(10.21,55.37)	7.05(3.03,16.24)	6.86(2.84,14.99)	0.04(-0.02,0.10)
Bahrain	0.80(0.17,2.40)	3.23(0.73,9.18)	1.05(0.22,3.16)	0.84(0.19,2.42)	-0.89(-1.01,-0.78)	25.59(10.71,61.13)	109.19(47.65,243.28)	22.99(9.10,57.91)	18.33(7.72,41.91)	-0.86(-0.92,-0.80)
Bangladesh	267.37(59.32,773.73)	781.12(186.65,2194.04)	0.82(0.18,2.32)	0.76(0.18,2.15)	-0.22(-0.38,-0.06)	6304.87(2616.09,14711.63)	17971.41(7384.57,42014.21)	16.93(7.00,39.76)	15.29(6.15,35.46)	-0.26(-0.44,-0.09)
Barbados	0.94(0.21,2.79)	1.36(0.30,3.94)	0.30(0.07,0.88)	0.26(0.06,0.76)	-0.73(-0.99,-0.46)	21.70(9.23,49.51)	29.79(12.58,69.11)	6.84(2.92,15.42)	5.64(2.39,13.02)	-0.89(-1.10,-0.67)
Belarus	71.75(15.64,202.33)	90.89(21.02,269.24)	0.61(0.13,1.73)	0.54(0.13,1.62)	-0.23(-0.37,-0.09)	1835.02(763.17,3987.39)	2322.30(1010.42,5421.25)	14.64(6.17,31.56)	14.01(6.12,32.39)	0.02(-0.10,0.13)
Belgium	218.32(49.32,648.31)	286.47(67.41,782.23)	1.38(0.31,4.08)	0.94(0.22,2.58)	-1.25(-1.44,-1.06)	4885.16(2106.13,11361.23)	5504.23(2250.41,12448.71)	30.55(13.17,71.96)	20.51(8.70,46.98)	-1.29(-1.42,-1.15)
Belize	0.38(0.09,1.10)	0.89(0.20,2.54)	0.42(0.10,1.21)	0.36(0.08,1.02)	-0.25(-0.34,-0.16)	9.20(4.12,21.01)	22.54(9.39,50.87)	10.18(4.56,23.23)	8.56(3.53,19.25)	-0.35(-0.44,-0.25)
Benin	2.62(0.58,7.50)	3.62(0.76,10.75)	0.16(0.04,0.47)	0.09(0.02,0.29)	-1.99(-2.18,-1.80)	72.67(29.64,175.72)	106.09(42.82,260.16)	4.05(1.63,9.79)	2.37(0.95,5.85)	-1.93(-2.11,-1.75)
Bermuda	0.19(0.04,0.57)	0.62(0.14,1.66)	0.36(0.08,1.06)	0.39(0.09,1.06)	0.30(0.26,0.34)	5.31(2.34,12.11)	14.31(6.16,30.85)	9.06(3.95,20.78)	9.70(4.20,21.09)	0.23(0.20,0.26)
Bhutan	0.53(0.11,1.54)	2.27(0.51,6.30)	0.42(0.08,1.24)	0.46(0.10,1.28)	0.34(0.30,0.37)	13.75(5.24,32.59)	45.20(16.89,101.61)	8.58(3.15,20.68)	8.49(3.17,18.94)	-0.03(-0.05,-0.02)
Bolivia (Plurinational Sta	6.73(1.50,19.67)	16.91(3.53,48.62)	0.30(0.06,0.86)	0.24(0.05,0.69)	-0.42(-0.55,-0.28)	189.40(78.36,444.15)	456.27(185.66,1103.65)	7.04(2.89,16.69)	5.68(2.26,13.38)	-0.46(-0.62,-0.30)
Bosnia and Herzegovina	27.63(6.68,76.75)	62.06(13.79,172.35)	0.96(0.23,2.63)	0.99(0.22,2.75)	0.19(-0.18,0.56)	751.56(321.31,1598.95)	1436.24(643.61,3239.97)	22.05(9.29,48.06)	22.52(10.17,50.20)	0.22(-0.15,0.59)
Botswana	2.41(0.53,6.96)	5.07(1.12,14.48)	0.78(0.16,2.38)	0.55(0.12,1.62)	-1.13(-1.23,-1.03)	71.17(29.49,166.62)	139.79(54.72,332.89)	17.25(6.70,40.67)	12.24(4.76,29.44)	-1.08(-1.18,-0.98)
Brazil	991.62(225.90,2842.22)	2152.53(515.67,5881.49)	1.59(0.36,4.51)	0.90(0.22,2.47)	-2.03(-2.22,-1.84)	25559.49(10425.72,60213.33)	50696.69(22036.70,118830.45)	34.69(14.31,82.16)	20.71(8.99,48.40)	-1.87(-2.06,-1.67)
Brunei Darussalam	0.69(0.16,2.12)	1.14(0.24,3.41)	1.00(0.22,3.11)	0.57(0.12,1.69)	-1.68(-1.76,-1.60)	17.81(7.70,44.02)	33.27(14.11,77.69)	22.16(9.22,55.70)	12.54(5.21,29.74)	-1.76(-1.84,-1.68)
Bulgaria	56.43(12.56,160.73)	54.45(11.69,159.28)	0.57(0.13,1.72)	0.37(0.08,1.09)	-1.52(-1.61,-1.43)	1947.73(865.14,4162.67)	1644.16(728.27,3797.49)	16.42(7.11,35.78)	11.54(4.97,26.98)	-1.24(-1.32,-1.16)
Burkina Faso	3.54(0.81,10.44)	6.32(1.35,18.72)	0.13(0.03,0.38)	0.10(0.02,0.29)	-0.77(-1.03,-0.51)	104.65(41.24,251.81)	179.97(71.55,436.54)	2.94(1.13,7.27)	2.31(0.89,5.57)	-0.76(-1.02,-0.51)
Burundi	4.65(0.98,13.25)	5.26(1.20,15.02)	0.33(0.07,0.95)	0.18(0.04,0.54)	-1.78(-2.15,-1.42)	128.41(52.01,291.01)	153.76(60.87,360.14)	7.13(2.91,16.67)	4.03(1.61,9.59)	-1.68(-2.07,-1.28)
Cabo Verde	0.38(0.08,1.18)	0.38(0.08,1.14)	0.16(0.04,0.50)	0.09(0.02,0.27)	-2.14(-2.41,-1.87)	9.34(3.78,22.59)	10.03(3.76,24.83)	4.03(1.63,9.84)	2.29(0.87,5.62)	-2.01(-2.24,-1.78)
Cambodia	29.23(6.71,86.20)	96.46(21.73,276.64)	1.18(0.26,3.45)	1.29(0.28,3.81)	0.26(0.07,0.45)	815.33(348.30,1890.43)	2456.24(1004.86,5893.47)	25.27(10.83,60.19)	26.06(10.55,63.42)	-0.04(-0.24,0.17)
Cameroon	6.03(1.26,17.07)	11.55(2.34,34.15)	0.22(0.04,0.60)	0.16(0.03,0.47)	-1.05(-1.12,-0.99)	162.80(61.99,386.84)	315.13(121.19,766.40)	4.63(1.68,11.09)	3.26(1.15,8.07)	-1.08(-1.13,-1.02)
Canada	402.79(89.62,1145.38)	682.58(161.84,1838.47)	1.29(0.28,3.63)	0.81(0.19,2.17)	-1.63(-1.74,-1.52)	10491.88(4983.50,22254.12)	15559.39(7149.04,33726.07)	32.58(15.46,69.30)	19.87(9.19,43.67)	-1.80(-1.90,-1.70)
Central African Republic	1.76(0.39,5.23)	2.22(0.47,6.53)	0.31(0.06,0.94)	0.21(0.04,0.62)	-1.31(-1.56,-1.07)	55.29(22.25,135.22)	73.38(28.36,185.62)	6.91(2.68,17.17)	4.77(1.82,11.73)	-1.29(-1.55,-1.03)
Chad	4.54(0.98,14.01)	7.04(1.50,20.95)	0.23(0.05,0.69)	0.21(0.04,0.59)	-0.49(-0.56,-0.43)	117.06(48.21,281.56)	184.88(70.90,434.66)	4.95(2.03,12.19)	4.27(1.62,10.13)	-0.64(-0.71,-0.57)
Chile	27.40(6.44,75.93)	65.32(15.29,190.99)	0.32(0.07,0.87)	0.25(0.06,0.72)	-1.02(-1.10,-0.94)	889.74(405.11,1983.81)	1884.46(824.93,4237.20)	9.30(4.20,20.77)	7.23(3.17,16.22)	-1.00(-1.08,-0.93)
China	8126.47(1822.19,22577.84)	24897.31(5866.82,70648.41)	1.72(0.39,4.84)	1.41(0.33,3.96)	-0.74(-0.83,-0.65)	214824.12(88552.25,498168.63)	602501.08(257945.38,1379582.96)	34.50(13.61,80.31)	30.63(12.98,69.65)	-0.49(-0.58,-0.39)
Colombia	52.22(12.02,153.85)	142.72(33.72,382.08)	0.38(0.09,1.12)	0.24(0.06,0.66)	-1.58(-1.68,-1.47)	1493.03(666.56,3401.20)	3502.71(1513.85,7788.08)	9.71(4.30,21.85)	6.18(2.68,13.79)	-1.57(-1.68,-1.46)
Comoros	0.54(0.11,1.56)	1.46(0.30,4.23)	0.51(0.11,1.51)	0.47(0.10,1.32)	-0.34(-0.38,-0.31)	14.48(5.63,34.12)	32.22(11.99,79.46)	10.40(3.90,24.71)	8.63(3.14,20.85)	-0.70(-0.74,-0.66)
Congo	2.09(0.48,6.27)	6.32(1.43,18.11)	0.35(0.08,1.02)	0.43(0.10,1.21)	0.90(0.68,1.12)	59.36(22.61,144.92)	174.36(66.10,407.14)	7.46(2.89,18.43)	9.00(3.43,21.69)	0.88(0.65,1.11)
Cook Islands	0.06(0.01,0.17)	0.12(0.03,0.36)	0.62(0.14,1.83)	0.47(0.10,1.46)	-0.96(-1.00,-0.91)	1.58(0.65,3.69)	2.99(1.27,7.15)	14.58(5.97,34.96)	11.45(4.85,27.31)	-0.88(-0.93,-0.83)
Costa Rica	9.69(2.36,27.03)	23.94(5.46,67.07)	0.63(0.15,1.74)	0.42(0.09,1.17)	-1.28(-1.50,-1.06)	236.67(103.25,515.10)	552.03(238.61,1253.64)	14.68(6.38,32.10)	9.90(4.27,22.45)	-1.25(-1.43,-1.07)
Croatia	57.73(12.76,157.59)	101.99(22.70,283.67)	1.21(0.26,3.24)	1.02(0.23,2.82)	-0.13(-0.29,0.04)	1450.75(611.23,3310.36)	2162.68(934.45,4942.39)	26.65(11.23,59.96)	22.38(9.73,51.34)	-0.15(-0.30,0.01)
Cuba	66.14(14.24,191.77)	106.73(22.52,310.03)	0.73(0.15,2.14)	0.50(0.11,1.46)	-1.46(-1.57,-1.35)	1774.93(752.86,3905.97)	2621.47(1086.58,6093.71)	18.17(7.69,40.43)	12.77(5.31,29.71)	-1.40(-1.54,-1.27)
Cyprus	6.39(1.54,18.28)	17.48(4.18,46.74)	1.12(0.25,3.23)	0.86(0.20,2.34)	-1.11(-1.20,-1.02)	174.14(72.87,406.97)	419.57(185.59,924.28)	24.34(9.82,57.03)	19.94(8.82,43.96)	-0.77(-0.81,-0.73)
Czechia	105.43(24.20,292.46)	163.41(37.96,458.63)	0.83(0.18,2.24)	0.69(0.16,1.95)	-0.60(-0.64,-0.57)	2784.10(1247.15,6265.92)	3954.63(1741.93,9022.94)	20.47(8.93,45.85)	17.57(7.80,40.33)	-0.52(-0.54,-0.50)
Côte d'Ivoire	5.10(1.07,15.10)	12.35(2.61,37.49)	0.25(0.05,0.75)	0.19(0.04,0.56)	-1.46(-1.69,-1.22)	151.51(58.21,372.39)	352.31(142.10,853.27)	5.35(1.98,13.00)	4.08(1.65,10.01)	-1.36(-1.59,-1.13)
Democratic People's Rep	65.70(14.00,186.36)	139.87(31.81,422.62)	0.63(0.14,1.80)	0.50(0.12,1.49)	-0.89(-0.97,-0.82)	1904.40(788.37,4486.22)	3774.03(1497.98,8982.05)	14.53(6.03,33.33)	12.16(4.79,29.73)	-0.74(-0.79,-0.68)
Democratic Republic of	17.07(3.62,54.44)	33.92(7.43,97.79)	0.21(0.04,0.67)	0.15(0.03,0.42)	-1.07(-1.36,-0.77)	541.18(224.42,1289.46)	1043.72(388.88,2433.59)	4.67(1.88,11.69)	3.49(1.31,8.23)	-0.88(-1.16,-0.60)
Denmark	205.30(47.18,579.16)	197.43(46.26,562.31)	2.33(0.53,6.49)	1.40(0.33,3.98)	-1.83(-2.03,-1.63)	3932.08(1628.05,8935.83)	3541.38(1368.35,8406.12)	44.94(18.73,103.15)	26.39(10.06,62.59)	-1.89(-2.06,-1.72)
Djibouti	0.44(0.10,1.30)	2.01(0.41,5.83)	0.74(0.15,2.12)	0.68(0.14,1.87)	-0.11(-0.17,-0.05)	12.69(5.19,29.66)	55.70(22.00,130.48)	15.03(5.90,35.62)	13.36(5.09,30.54)	-0.24(-0.31,-0.18)
Dominica	0.14(0.03,0.39)	0.16(0.03,0.48)	0.25(0.05,0.71)	0.21(0.05,0.62)	-0.50(-0.67,-0.33)	3.62(1.57,8.04)	4.39(1.80,10.45)	6.17(2.65,13.76)	5.37(2.24,12.85)	-0.42(-0.61,-0.23)
Dominican Republic	26.16(5.97,79.27)	73.47(16.08,211.63)	1.03(0.23,3.03)	0.78(0.17,2.25)	-0.85(-1.10,-0.60)	640.22(276.67,1480.84)	1601.42(669.67,3655.44)	21.70(9.20,50.74)	16.84(7.02,38.61)	-0.86(-1.09,-0.63)
Ecuador	17.86(3.96,52.26)	31.35(6.55,94.03)	0.43(0.09,1.24)	0.21(0.04,0.62)	-2.27(-2.35,-2.19)	453.51(191.26,1042.44)	782.56(328.75,1838.49)	9.77(4.09,22.86)	4.99(2.08,11.69)	-2.18(-2.27,-2.09)
Egypt	167.76(38.73,486.79)	395.40(85.50,1137.77)	1.36(0.30,4.01)	1.44(0.33,4.13)	0.28(0.18,0.39)	4436.43(1810.40,10396.14)	10259.33(4294.74,23340.60)	26.42(10.55,63.56)	27.07(11.54,62.68)	0.19(0.10,0.28)
El Salvador	5.98(1.37,17.43)	14.71(3.32,41.12)	0.22(0.05,0.63)	0.22(0.05,0.62)	0.06(0.01,0.12)	155.78(67.30,350.75)	363.46(154.81,846.04)	5.54(2.39,12.44)	5.69(2.43,13.31)	0.20(0.12,0.28)
Equatorial Guinea	0.33(0.07,1.01)	0.80(0.16,2.39)	0.30(0.06,0.92)	0.27(0.06,0.78)	-0.26(-0.33,-0.20)	9.67(3.74,24.30)	22.03(8.65,51.59)	6.66(2.52,17.07)	5.76(2.21,13.82)	-0.38(-0.44,-0.31)
Eritrea	0.68(0.14,2.00)	1.78(0.36,5.46)	0.13(0.03,0.38)	0.12(0.02,0.36)	-0.37(-0.51,-0.23)	25.78(9.98,60.18)	56.82(21.24,138.40)	3.10(1.18,7.35)	2.66(1.00,6.72)	-0.61(-0.76,-0.47)
Estonia	8.27(1.77,23.50)	15.79(3.64,43.38)	0.42(0.09,1.21)	0.49(0.11,1.35)	0.64(0.38,0.90)	245.06(107.12,553.11)	384.78(167.78,837.63)	11.94(5.18,26.77)	13.52(5.91,29.81)	0.55(0.31,0.80)
Eswatini	0.85(0.17,2.55)	0.99(0.20,2.91)	0.56(0.12,1.72)	0.38(0.07,1.13)	-1.24(-1.32,-1.17)	20.95(7.98,51.48)	25.90(9.52,61.43)	10.98(4.02,27.15)	7.32(2.65,18.09)	-1.36(-1.44,-1.28)
Ethiopia	17.65(3.53,51.77)	43.31(9.74,133.82)	0.14(0.03,0.41)	0.14(0.03,0.43)	0.65(0.39,0.92)	564.91(217.74,1368.28)	1108.92(429.51,2765.34)	3.41(1.32,8.33)	3.09(1.20,7.73)	0.10(-0.13,0.33)
Fiji	1.11(0.25,3.23)	1.88(0.39,5.42)	0.46(0.10,1.35)	0.33(0.07,0.98)	-1.10(-1.27,-0.92)	37.24(15.38,85.89)	64.28(26.35,143.83)	12.56(5.18,29.01)	9.12(3.80,21.36)	-1.06(-1.20,-0.91)
Finland	43.30(10.67,126.59)	85.38(19.26,234.95)	0.61(0.15,1.81)	0.54(0.12,1.48)	-0.13(-0.34,0.07)	1023.23(425.21,2418.61)	1750.31(712.63,3913.55)	14.21(5.95,33.73)	12.50(5.19,28.00)	-0.19(-0.37,-0.00)
France	773.07(174.13,2162.80)	1100.95(263.91,3050.88)	0.85(0.19,2.39)	0.59(0.14,1.63)	-1.35(-1.44,-1.26)	16293.53(6949.80,37436.52)	21153.30(8755.92,48498.06)	18.34(7.78,42.23)	13.32(5.69,30.35)	-1.11(-1.14,-1.07)
Gabon	1.01(0.23,2.94)	1.73(0.36,5.04)	0.24(0.06,0.65)	0.25(0.05,0.69)	0.01(-0.10,0.12)	25.73(10.05,64.38)	48.13(18.31,119.24)	5.19(2.03,12.67)	5.49(2.10,13.38)	0.17(0.11,0.23)
Gambia	0.63(0.14,1.79)	1.21(0.27,3.48)	0.28(0.06,0.80)	0.17(0.04,0.48)	-1.78(-1.89,-1.68)	19.21(7.77,44.87)	32.84(12.42,80.02)	6.72(2.65,15.62)	3.96(1.49,9.63)	-1.96(-2.08,-1.84)
Georgia	26.21(6.18,79.71)	47.02(10.56,139.25)	0.47(0.11,1.44)	0.69(0.15,2.05)	1.74(1.60,1.89)	728.91(332.83,1675.51)	1062.03(458.58,2541.60)	12.03(5.42,28.03)	16.47(7.15,39.30)	1.49(1.35,1.63)
Germany	1539.99(365.46,4155.95)	2199.40(555.21,6097.99)	1.17(0.28,3.16)	0.87(0.22,2.42)	-1.04(-1.22,-0.86)	34212.87(15508.78,76344.75)	44416.01(19909.80,99500.15)	25.88(11.66,57.88)	19.76(8.82,43.80)	-0.88(-1.02,-0.74)
Ghana	6.93(1.51,20.40)	20.94(4.71,62.90)	0.23(0.05,0.67)	0.23(0.05,0.70)	0.54(0.27,0.81)	171.75(68.22,404.16)	500.20(191.37,1215.42)	4.28(1.70,10.06)	4.36(1.64,10.62)	0.52(0.24,0.80)
Greece	220.37(48.49,611.84)	382.76(87.13,1071.76)	1.59(0.36,4.48)	1.13(0.26,3.16)	-1.13(-1.19,-1.08)	5020.41(2110.29,11403.02)	7055.37(2895.75,15944.27)	33.76(14.06,76.65)	24.25(10.19,54.61)	-1.06(-1.10,-1.02)
Greenland	0.25(0.06,0.73)	0.52(0.11,1.48)	1.33(0.31,3.87)	1.08(0.24,3.08)	-0.71(-0.75,-0.67)	8.48(3.78,18.21)	16.55(7.30,38.43)	33.07(14.82,73.33)	26.93(11.82,61.12)	-0.72(-0.74,-0.69)
Grenada	0.21(0.05,0.64)	0.16(0.04,0.47)	0.25(0.06,0.76)	0.17(0.04,0.51)	-1.48(-1.66,-1.29)	4.95(2.19,11.50)	4.98(2.26,11.79)	6.34(2.80,14.74)	4.63(2.06,10.80)	-1.24(-1.38,-1.10)
Guam	0.19(0.04,0.57)	0.80(0.18,2.20)	0.38(0.09,1.11)	0.34(0.07,0.96)	-0.23(-0.29,-0.18)	6.72(2.81,16.41)	21.60(8.81,51.32)	10.06(4.26,24.18)	9.70(3.97,23.00)	-0.06(-0.10,-0.03)
Guatemala	8.19(1.83,25.13)	27.99(6.07,84.67)	0.40(0.09,1.21)	0.31(0.07,0.92)	-0.95(-1.09,-0.82)	233.06(100.92,550.21)	691.06(297.09,1556.64)	9.06(3.87,21.17)	6.99(2.99,16.16)	-0.91(-1.03,-0.78)
Guinea	5.73(1.19,16.07)	8.37(1.82,23.88)	0.24(0.05,0.67)	0.21(0.04,0.60)	-0.62(-0.67,-0.57)	146.85(59.02,342.80)	212.44(85.98,496.47)	5.27(2.12,12.51)	4.54(1.77,10.52)	-0.56(-0.61,-0.51)
Guinea-Bissau	0.24(0.05,0.72)	0.45(0.09,1.36)	0.10(0.02,0.30)	0.11(0.02,0.33)	0.45(0.17,0.73)	7.12(2.68,17.54)	14.11(5.03,35.42)	2.29(0.85,5.73)	2.47(0.92,6.51)	0.55(0.26,0.84)
Guyana	0.77(0.16,2.23)	1.10(0.23,3.39)	0.28(0.06,0.80)	0.23(0.05,0.70)	-0.53(-0.63,-0.43)	21.77(9.67,49.22)	31.95(13.50,75.58)	6.83(2.98,15.53)	5.75(2.48,13.40)	-0.54(-0.62,-0.45)
Haiti	4.04(0.90,12.27)	7.34(1.69,21.29)	0.20(0.04,0.58)	0.17(0.04,0.47)	-0.46(-0.56,-0.36)	131.09(54.06,306.37)	216.67(94.62,497.99)	5.03(2.04,11.78)	3.94(1.72,8.73)	-0.77(-0.90,-0.65)
Honduras	8.27(1.96,24.82)	24.83(5.45,72.99)	0.53(0.12,1.57)	0.54(0.12,1.56)	0.13(0.05,0.20)	226.41(96.59,511.99)	656.49(255.48,1595.82)	12.85(5.42,29.15)	12.14(4.76,29.12)	-0.16(-0.21,-0.11)
Hungary	86.16(20.77,249.76)	95.66(21.14,268.84)	0.64(0.15,1.86)	0.45(0.10,1.25)	-1.20(-1.23,-1.16)	2527.26(1103.16,5755.67)	2527.40(1083.12,5482.49)	17.39(7.51,39.69)	12.62(5.44,27.44)	-1.06(-1.09,-1.03)
Iceland	4.78(1.10,13.16)	6.11(1.42,16.27)	1.52(0.35,4.20)	0.87(0.21,2.33)	-2.04(-2.13,-1.94)	100.21(42.62,215.02)	122.78(51.98,267.17)	33.01(14.25,70.74)	19.08(8.13,40.90)	-1.97(-2.05,-1.89)
India	1468.86(318.82,4366.27)	4320.84(956.61,12556.70)	0.61(0.13,1.81)	0.52(0.12,1.53)	-0.46(-0.59,-0.34)	39665.09(17118.78,95221.44)	98343.86(40167.81,236215.30)	12.70(5.39,30.15)	10.25(4.28,24.93)	-0.76(-0.83,-0.69)
Indonesia	387.38(83.56,1147.78)	1155.87(276.47,3446.99)	0.68(0.15,1.98)	0.87(0.20,2.55)	0.72(0.45,0.99)	11536.94(5301.60,26170.21)	32681.90(13974.34,79988.05)	16.01(7.14,36.82)	18.42(7.78,44.41)	0.40(0.20,0.60)
Iran (Islamic Republic o	102.31(23.91,288.55)	399.85(87.79,1167.70)	0.64(0.15,1.79)	0.64(0.14,1.85)	0.21(0.13,0.29)	3052.86(1306.78,7012.10)	9751.68(4043.63,22313.10)	14.74(6.31,34.53)	14.22(5.85,32.91)	0.04(-0.03,0.10)
Iraq	125.35(28.10,340.92)	176.39(37.91,516.27)	1.84(0.41,4.99)	1.26(0.28,3.59)	-1.34(-1.39,-1.29)	2613.88(1098.24,5830.07)	4309.34(1744.80,10587.62)	37.47(15.80,83.30)	25.36(10.28,59.46)	-1.35(-1.39,-1.31)
Ireland	75.19(16.47,208.20)	83.94(19.84,230.71)	2.12(0.46,5.88)	0.97(0.23,2.65)	-2.82(-2.92,-2.71)	1609.04(706.73,3649.78)	1646.87(708.83,3754.80)	41.25(17.96,96.14)	19.48(8.36,44.55)	-2.70(-2.80,-2.60)
Israel	44.72(9.73,132.18)	96.59(22.47,261.52)	1.06(0.23,3.15)	0.68(0.16,1.83)	-1.50(-1.62,-1.37)	1054.06(425.45,2421.11)	1939.09(772.42,4244.39)	22.79(9.18,52.68)	14.58(5.88,32.10)	-1.48(-1.57,-1.39)
Italy	1138.42(262.81,3154.53)	1499.65(334.35,3900.92)	1.33(0.30,3.65)	0.77(0.18,2.03)	-1.78(-1.83,-1.73)	24651.23(10166.97,56683.33)	29262.67(12215.73,64625.46)	27.62(11.44,63.38)	17.40(7.47,37.94)	-1.48(-1.51,-1.44)
Jamaica	8.23(1.83,25.28)	12.25(2.68,34.91)	0.44(0.10,1.36)	0.35(0.07,0.99)	-1.02(-1.12,-0.93)	213.25(96.38,492.89)	287.72(124.24,634.60)	11.66(5.23,26.99)	8.87(3.89,19.39)	-1.12(-1.21,-1.03)
Japan	1685.67(401.72,4781.23)	3727.87(903.72,9970.37)	1.14(0.26,3.19)	0.68(0.16,1.85)	-1.83(-1.92,-1.75)	39011.13(16396.31,89582.55)	67192.09(28223.44,150478.57)	24.26(10.15,55.19)	15.37(6.60,35.52)	-1.58(-1.64,-1.52)
Jordan	14.30(3.33,40.48)	56.54(13.70,153.80)	1.92(0.44,5.35)	1.37(0.32,3.66)	-1.25(-1.39,-1.11)	346.81(148.67,797.01)	1491.52(667.58,3303.62)	38.17(16.33,88.33)	28.24(12.25,63.16)	-1.08(-1.18,-0.98)
Kazakhstan	33.41(7.25,94.54)	39.27(8.69,116.73)	0.32(0.07,0.88)	0.28(0.06,0.81)	-0.28(-0.42,-0.15)	1012.76(442.18,2302.68)	1311.66(558.14,2973.39)	8.59(3.72,19.82)	7.79(3.34,17.96)	-0.20(-0.35,-0.06)
Kenya	26.10(5.91,77.30)	39.21(8.70,108.99)	0.52(0.12,1.53)	0.29(0.07,0.80)	-2.34(-2.52,-2.16)	651.64(277.81,1523.14)	1056.81(426.25,2450.50)	10.50(4.43,25.21)	6.06(2.37,13.97)	-2.20(-2.37,-2.02)
Kiribati	0.23(0.05,0.66)	0.50(0.11,1.41)	1.02(0.23,3.00)	1.22(0.27,3.53)	0.39(0.07,0.70)	7.07(2.98,16.64)	15.51(6.61,35.38)	24.63(10.06,57.99)	28.21(11.93,65.35)	0.25(-0.05,0.56)
Kuwait	3.95(0.89,10.98)	20.94(4.88,59.41)	1.14(0.25,3.16)	1.07(0.26,3.03)	-0.26(-0.40,-0.13)	106.15(46.13,235.53)	475.32(217.24,1076.42)	25.51(10.89,56.31)	22.08(9.83,51.15)	-0.51(-0.59,-0.44)
Kyrgyzstan	12.98(3.05,37.25)	28.39(6.23,81.10)	0.53(0.12,1.53)	0.81(0.17,2.29)	2.29(1.97,2.62)	327.70(144.56,735.37)	752.95(315.56,1722.50)	12.09(5.24,27.60)	18.70(7.88,43.49)	2.28(1.98,2.59)
Lao People's Democratic	9.33(2.06,27.08)	26.15(6.40,73.13)	0.82(0.17,2.33)	0.93(0.23,2.54)	0.52(0.44,0.60)	294.46(128.24,657.68)	695.43(299.41,1605.34)	19.34(8.50,43.51)	20.05(8.63,45.10)	0.22(0.17,0.26)
Latvia	15.86(3.42,46.81)	16.88(3.64,49.56)	0.47(0.10,1.36)	0.37(0.08,1.10)	-0.68(-0.76,-0.59)	436.30(191.82,975.76)	432.62(188.19,988.46)	12.21(5.29,27.50)	10.45(4.57,24.04)	-0.35(-0.43,-0.27)
Lebanon	20.85(4.47,60.99)	108.26(26.89,299.17)	1.35(0.29,3.81)	1.59(0.39,4.44)	0.54(0.24,0.85)	556.64(246.31,1239.61)	2291.51(979.21,5108.80)	30.97(13.66,69.25)	35.63(15.39,80.46)	0.41(0.12,0.69)
Lesotho	4.28(0.86,12.45)	4.69(1.05,13.02)	0.76(0.15,2.24)	0.85(0.18,2.39)	0.72(0.61,0.83)	98.34(37.73,231.02)	120.55(46.98,274.04)	15.03(5.57,35.63)	16.70(6.26,37.66)	0.61(0.52,0.69)
Liberia	1.20(0.26,3.50)	1.53(0.32,4.63)	0.15(0.03,0.44)	0.11(0.02,0.31)	-1.35(-1.43,-1.27)	34.44(13.89,82.94)	46.90(18.62,113.44)	3.56(1.40,8.48)	2.62(1.03,6.38)	-1.15(-1.21,-1.09)
Libya	17.24(3.99,46.57)	32.29(7.21,96.06)	1.12(0.26,3.06)	0.87(0.19,2.58)	-0.60(-0.69,-0.51)	369.56(151.46,847.08)	740.27(311.15,1742.98)	22.67(9.23,50.81)	17.71(7.25,42.21)	-0.65(-0.73,-0.57)
Lithuania	25.56(5.69,73.40)	30.41(6.66,91.43)	0.60(0.13,1.71)	0.44(0.10,1.33)	-1.15(-1.21,-1.10)	601.96(265.18,1372.99)	690.70(293.11,1674.98)	13.48(5.92,30.84)	10.95(4.65,26.18)	-0.70(-0.72,-0.67)
Luxembourg	3.25(0.76,9.81)	6.75(1.51,19.88)	0.62(0.14,1.86)	0.56(0.13,1.63)	-0.30(-0.42,-0.18)	84.42(36.11,197.11)	146.36(60.80,334.94)	15.46(6.62,35.88)	13.01(5.38,29.38)	-0.55(-0.64,-0.46)
Madagascar	11.42(2.64,33.48)	8.64(1.85,25.54)	0.41(0.09,1.25)	0.18(0.04,0.51)	-2.85(-3.23,-2.47)	289.04(123.39,680.81)	254.88(107.99,588.77)	8.25(3.39,19.30)	3.62(1.47,8.59)	-2.82(-3.19,-2.44)
Malawi	11.83(2.54,35.97)	21.65(4.69,63.47)	0.60(0.12,1.84)	0.48(0.11,1.46)	-0.71(-0.76,-0.66)	312.58(125.62,760.71)	573.25(234.07,1414.94)	11.97(4.78,29.57)	10.30(4.03,25.42)	-0.51(-0.54,-0.47)
Malaysia	80.62(18.48,234.12)	156.39(35.35,455.48)	1.09(0.25,3.20)	0.79(0.18,2.32)	-1.33(-1.46,-1.21)	1724.70(723.37,3927.75)	3533.27(1523.02,8417.42)	22.01(9.25,50.65)	15.29(6.50,36.71)	-1.38(-1.51,-1.25)
Maldives	0.61(0.14,1.81)	2.55(0.61,7.25)	1.65(0.36,4.90)	1.04(0.25,2.96)	-1.56(-1.62,-1.50)	17.90(7.92,39.78)	58.24(25.77,132.05)	33.61(14.82,77.68)	21.71(9.41,49.02)	-1.53(-1.63,-1.43)
Mali	4.73(1.06,13.04)	17.37(3.90,52.08)	0.23(0.05,0.67)	0.39(0.08,1.14)	2.03(1.88,2.18)	120.79(46.77,282.23)	422.36(161.76,1043.93)	4.44(1.71,10.35)	7.04(2.61,17.71)	1.88(1.74,2.01)
Malta	3.20(0.72,9.04)	6.21(1.47,16.69)	0.86(0.19,2.41)	0.55(0.13,1.48)	-1.81(-1.93,-1.68)	82.19(34.81,183.75)	137.45(56.81,294.79)	20.29(8.50,45.17)	13.14(5.49,28.40)	-1.67(-1.75,-1.58)
Marshall Islands	0.05(0.01,0.14)	0.08(0.02,0.25)	0.47(0.10,1.37)	0.49(0.10,1.52)	0.17(0.15,0.19)	1.31(0.50,3.27)	2.66(1.03,6.28)	10.38(4.01,25.73)	10.70(4.16,26.64)	0.11(0.10,0.13)
Mauritania	1.10(0.24,3.05)	2.16(0.48,6.56)	0.16(0.04,0.44)	0.13(0.03,0.41)	-0.58(-0.63,-0.53)	32.10(13.06,71.83)	58.45(22.70,137.31)	3.72(1.51,8.42)	3.06(1.17,7.30)	-0.73(-0.78,-0.68)
Mauritius	3.26(0.75,9.51)	10.31(2.39,29.10)	0.65(0.15,1.92)	0.62(0.14,1.80)	-0.12(-0.54,0.30)	92.57(40.24,215.36)	253.71(112.66,554.31)	15.25(6.54,36.11)	14.29(6.26,32.35)	-0.14(-0.53,0.25)
Mexico	191.11(42.43,550.41)	287.23(61.48,799.94)	0.68(0.15,1.94)	0.26(0.06,0.73)	-3.30(-3.51,-3.08)	5022.11(2274.14,11258.94)	7064.80(3026.66,15520.42)	14.94(6.79,34.71)	6.08(2.60,13.28)	-3.14(-3.32,-2.95)
Micronesia (Federated S	0.25(0.05,0.70)	0.31(0.07,0.92)	0.70(0.15,1.98)	0.62(0.13,1.87)	-0.48(-0.51,-0.44)	7.40(3.00,17.68)	10.44(3.92,24.60)	17.42(6.97,41.14)	15.85(5.99,37.29)	-0.37(-0.40,-0.34)
Monaco	0.85(0.19,2.35)	1.11(0.26,3.09)	1.01(0.23,2.84)	0.87(0.20,2.48)	-0.51(-0.55,-0.48)	17.89(7.57,39.42)	20.82(8.43,48.90)	22.83(9.65,50.85)	18.40(7.66,42.22)	-0.74(-0.77,-0.71)
Mongolia	3.76(0.85,11.04)	8.74(1.95,25.39)	0.46(0.10,1.32)	0.58(0.13,1.66)	1.13(0.94,1.33)	113.17(48.01,261.33)	272.69(118.27,640.71)	12.05(5.09,27.61)	14.72(6.50,35.00)	0.97(0.84,1.10)
Montenegro	4.97(1.13,13.92)	6.65(1.54,19.35)	0.88(0.20,2.46)	0.75(0.17,2.16)	-0.54(-0.71,-0.36)	140.84(62.51,326.74)	199.21(86.32,462.61)	23.56(10.45,55.20)	20.31(8.63,47.49)	-0.53(-0.75,-0.31)
Morocco	63.63(13.90,179.64)	89.16(18.24,252.23)	0.58(0.13,1.68)	0.32(0.07,0.91)	-2.06(-2.14,-1.97)	1635.49(714.54,3677.37)	2499.99(1020.75,5620.98)	13.27(5.79,30.49)	7.92(3.19,17.96)	-1.79(-1.91,-1.67)
Mozambique	14.23(3.20,41.50)	21.17(4.66,61.84)	0.46(0.10,1.37)	0.36(0.08,1.03)	-0.67(-0.80,-0.55)	364.04(147.50,861.86)	551.35(209.22,1362.99)	8.85(3.55,21.27)	7.04(2.66,16.98)	-0.62(-0.74,-0.50)
Myanmar	170.06(38.40,483.65)	239.85(53.22,729.09)	1.33(0.30,3.79)	0.71(0.16,2.08)	-2.24(-2.36,-2.13)	5031.45(2304.91,11010.88)	5923.78(2466.02,14355.87)	30.42(13.63,66.63)	14.93(6.01,36.30)	-2.48(-2.60,-2.37)
Namibia	3.42(0.81,10.59)	6.32(1.41,17.36)	1.14(0.26,3.60)	0.84(0.19,2.31)	-1.21(-1.36,-1.05)	87.48(37.25,212.35)	146.72(59.97,348.80)	21.76(8.94,55.88)	15.68(6.34,36.99)	-1.29(-1.41,-1.16)
Nauru	0.02(0.00,0.06)	0.02(0.00,0.06)	0.82(0.19,2.37)	0.59(0.12,1.69)	-1.18(-1.23,-1.14)	0.60(0.24,1.43)	0.64(0.26,1.53)	18.35(7.17,45.63)	14.06(5.83,33.77)	-0.95(-1.00,-0.91)
Nepal	59.13(13.16,169.48)	149.56(33.14,445.12)	1.19(0.27,3.43)	1.00(0.22,2.95)	-0.66(-0.76,-0.57)	1591.99(709.95,3560.99)	3349.84(1386.32,7936.14)	25.34(10.79,56.26)	18.86(7.51,45.64)	-1.16(-1.26,-1.07)
Netherlands	314.09(75.84,907.02)	486.23(108.62,1297.53)	1.55(0.37,4.46)	1.19(0.27,3.19)	-0.44(-0.58,-0.31)	6356.59(2732.05,14715.94)	9399.83(3801.53,21339.43)	31.08(13.28,72.44)	24.04(9.85,55.02)	-0.45(-0.59,-0.32)
New Zealand	38.74(9.19,110.70)	78.29(18.48,214.67)	1.04(0.24,2.95)	0.83(0.20,2.27)	-0.64(-0.73,-0.54)	935.45(403.62,2146.16)	1596.24(711.40,3645.03)	23.92(10.24,55.11)	17.55(7.80,40.04)	-0.98(-1.07,-0.88)
Nicaragua	4.68(1.10,13.39)	14.10(3.05,39.98)	0.39(0.09,1.16)	0.34(0.07,0.98)	0.00(-0.19,0.19)	125.92(55.39,280.97)	366.94(159.85,803.89)	9.57(4.18,21.44)	8.33(3.61,18.21)	-0.13(-0.30,0.04)
Niger	1.84(0.40,5.38)	6.31(1.38,19.37)	0.14(0.03,0.42)	0.17(0.04,0.52)	0.49(0.29,0.69)	48.34(19.47,117.00)	145.56(57.94,356.42)	2.75(1.11,6.55)	2.92(1.10,7.37)	0.06(-0.07,0.20)
Nigeria	43.24(8.95,124.76)	58.41(13.26,173.19)	0.16(0.03,0.45)	0.10(0.02,0.29)	-1.62(-1.66,-1.58)	1016.25(407.20,2407.00)	1489.12(571.55,3818.34)	3.08(1.17,7.29)	2.04(0.77,5.13)	-1.35(-1.43,-1.27)
Niue	0.01(0.00,0.03)	0.01(0.00,0.02)	0.46(0.10,1.31)	0.42(0.10,1.18)	-0.32(-0.36,-0.28)	0.26(0.10,0.61)	0.23(0.09,0.51)	11.60(4.49,26.98)	10.53(4.22,23.53)	-0.38(-0.43,-0.34)
North Macedonia	15.27(3.34,43.34)	20.36(4.53,59.27)	0.99(0.21,2.80)	0.88(0.19,2.40)	-0.46(-0.64,-0.27)	408.71(177.22,931.49)	607.40(271.63,1369.17)	24.04(10.51,53.58)	20.96(9.11,46.61)	-0.53(-0.64,-0.42)
Northern Mariana Island	0.05(0.01,0.15)	0.17(0.04,0.49)	0.59(0.14,1.71)	0.47(0.11,1.38)	-0.82(-0.87,-0.78)	1.67(0.73,3.89)	5.38(2.22,13.05)	14.17(6.03,33.65)	11.63(4.82,28.20)	-0.74(-0.79,-0.69)
Norway	96.38(20.94,265.77)	79.16(17.89,223.06)	1.25(0.27,3.44)	0.65(0.15,1.85)	-2.59(-2.86,-2.32)	2051.41(908.40,4626.97)	1538.49(649.57,3560.10)	27.68(12.14,62.18)	13.87(6.05,31.63)	-2.70(-2.95,-2.45)
Oman	2.26(0.50,6.51)	4.24(0.99,12.03)	0.53(0.11,1.50)	0.39(0.09,1.07)	-0.83(-0.96,-0.71)	60.44(24.59,141.59)	122.74(51.32,286.36)	11.77(4.71,27.66)	8.61(3.47,19.63)	-0.92(-1.03,-0.80)
Pakistan	331.69(71.72,948.23)	409.77(94.21,1253.29)	0.86(0.19,2.44)	0.55(0.13,1.70)	-1.61(-1.77,-1.46)	8288.26(3500.53,20118.94)	10207.77(4045.91,25450.62)	18.15(7.56,43.69)	11.15(4.35,27.71)	-1.73(-1.87,-1.60)
Palau	0.03(0.01,0.10)	0.06(0.02,0.19)	0.52(0.12,1.42)	0.47(0.11,1.36)	-0.38(-0.42,-0.34)	0.98(0.40,2.31)	1.99(0.81,4.59)	12.05(4.75,27.98)	10.76(4.33,25.13)	-0.45(-0.50,-0.40)
Palestine	7.53(1.60,22.21)	14.28(3.05,41.67)	1.20(0.26,3.62)	0.89(0.20,2.66)	-1.28(-1.41,-1.15)	181.99(76.68,419.01)	395.15(166.41,903.98)	25.59(10.58,60.15)	20.24(8.31,47.28)	-1.00(-1.11,-0.90)
Panama	7.35(1.69,20.89)	16.89(3.43,46.92)	0.58(0.13,1.64)	0.36(0.07,1.00)	-1.77(-1.91,-1.63)	169.35(74.83,374.33)	365.11(155.92,812.16)	12.68(5.55,28.31)	8.06(3.46,17.95)	-1.69(-1.82,-1.56)
Papua New Guinea	4.39(0.88,12.67)	11.36(2.50,33.39)	0.41(0.09,1.12)	0.35(0.08,1.05)	-0.65(-0.72,-0.57)	160.04(65.17,381.70)	401.05(166.00,936.45)	10.94(4.43,24.42)	9.48(3.87,22.18)	-0.61(-0.68,-0.54)
Paraguay	36.71(8.44,104.55)	67.18(14.46,177.64)	1.97(0.45,5.55)	1.30(0.28,3.47)	-1.46(-1.54,-1.38)	758.02(319.29,1751.64)	1351.57(554.16,2895.07)	38.88(16.23,90.66)	25.43(10.31,54.44)	-1.51(-1.58,-1.44)
Peru	28.35(6.43,84.33)	92.95(20.43,267.23)	0.28(0.06,0.84)	0.28(0.06,0.79)	-0.32(-0.47,-0.16)	618.71(258.14,1416.08)	1948.76(826.05,4654.09)	5.83(2.45,13.39)	5.88(2.50,14.07)	-0.26(-0.41,-0.11)
Philippines	206.25(46.68,600.10)	428.93(97.99,1274.47)	1.34(0.28,3.81)	0.77(0.17,2.31)	-1.79(-1.92,-1.67)	5901.60(2677.77,12870.95)	11239.34(4918.55,26001.47)	28.31(12.03,63.50)	16.83(7.16,39.08)	-1.74(-1.87,-1.61)
Poland	332.55(78.41,966.65)	446.22(106.70,1275.60)	0.85(0.19,2.54)	0.57(0.13,1.61)	-1.43(-1.50,-1.35)	9341.22(4075.39,21008.61)	11161.03(4636.79,25585.17)	22.08(9.56,50.28)	14.93(6.23,34.23)	-1.32(-1.34,-1.29)
Portugal	64.04(13.94,194.33)	90.43(21.40,258.67)	0.49(0.11,1.47)	0.30(0.07,0.84)	-1.80(-1.91,-1.69)	1744.25(756.70,4060.40)	2052.25(884.18,4607.54)	12.47(5.35,29.31)	7.92(3.46,17.86)	-1.63(-1.71,-1.55)
Puerto Rico	14.78(3.37,41.79)	34.99(7.91,98.04)	0.45(0.10,1.24)	0.37(0.09,1.06)	-0.81(-0.87,-0.75)	379.66(160.76,858.92)	748.29(320.89,1716.96)	10.76(4.58,24.20)	9.12(3.89,20.99)	-0.66(-0.71,-0.62)
Qatar	0.30(0.06,0.90)	1.99(0.42,5.84)	0.63(0.14,1.87)	0.48(0.11,1.31)	-1.16(-1.30,-1.02)	10.33(4.19,24.00)	78.42(33.53,176.33)	14.44(5.81,34.44)	11.60(4.60,26.51)	-0.74(-0.82,-0.67)
Republic of Korea	288.66(65.82,798.44)	985.52(230.94,2769.53)	1.89(0.43,5.35)	1.09(0.25,3.05)	-1.91(-1.99,-1.83)	6643.81(2695.46,15649.20)	19643.64(8044.07,46421.78)	33.71(12.91,80.41)	20.98(8.54,49.36)	-1.68(-1.76,-1.60)
Republic of Moldova	13.52(3.08,39.40)	26.59(5.82,75.08)	0.39(0.09,1.14)	0.43(0.09,1.22)	0.54(0.45,0.63)	423.49(182.65,981.95)	728.58(307.20,1627.54)	10.42(4.44,24.20)	11.86(5.04,26.42)	0.60(0.51,0.68)
Romania	121.16(27.36,351.11)	151.04(33.94,434.82)	0.57(0.13,1.66)	0.37(0.08,1.07)	-1.66(-1.86,-1.47)	3629.08(1589.37,8581.91)	3927.33(1714.71,8700.83)	14.26(6.22,33.02)	10.27(4.43,22.81)	-1.28(-1.43,-1.14)
Russian Federation	535.28(119.40,1543.95)	1131.32(262.83,3226.71)	0.35(0.07,1.00)	0.46(0.11,1.31)	1.14(0.75,1.53)	16434.17(7239.37,37589.14)	30294.43(13293.15,68272.85)	9.47(4.16,21.80)	12.31(5.45,27.66)	0.99(0.63,1.35)
Rwanda	14.93(3.48,43.76)	47.64(10.94,134.30)	1.18(0.26,3.67)	1.49(0.33,4.06)	0.91(0.81,1.00)	377.93(158.30,892.77)	1071.43(389.83,2612.57)	21.85(8.86,53.97)	26.26(9.46,61.29)	0.73(0.64,0.82)
Saint Kitts and Nevis	0.08(0.02,0.23)	0.09(0.02,0.28)	0.23(0.05,0.69)	0.19(0.04,0.55)	-0.67(-0.74,-0.59)	2.11(0.93,4.87)	2.65(1.10,6.25)	5.58(2.38,12.81)	4.52(1.89,10.51)	-0.82(-0.91,-0.72)
Saint Lucia	0.23(0.05,0.71)	0.55(0.12,1.66)	0.32(0.07,1.00)	0.24(0.05,0.73)	-1.11(-1.23,-0.98)	6.65(2.82,15.78)	14.25(6.05,34.04)	8.07(3.39,19.45)	6.00(2.55,14.32)	-1.19(-1.35,-1.04)
Saint Vincent and the Gr	0.15(0.04,0.44)	0.31(0.07,0.92)	0.23(0.05,0.68)	0.24(0.05,0.70)	0.06(-0.00,0.13)	4.14(1.79,9.64)	8.53(3.69,19.04)	6.00(2.57,13.95)	6.13(2.63,13.81)	0.04(-0.00,0.09)
Samoa	0.53(0.11,1.51)	0.86(0.19,2.46)	0.83(0.18,2.39)	0.78(0.18,2.33)	-0.37(-0.43,-0.30)	14.62(5.75,35.71)	22.78(9.03,53.05)	19.56(7.89,47.67)	17.82(6.98,41.65)	-0.46(-0.53,-0.39)
San Marino	0.36(0.08,1.04)	0.78(0.18,1.90)	0.96(0.22,2.75)	0.71(0.16,1.81)	-0.86(-0.92,-0.80)	8.06(3.54,17.76)	14.40(5.85,29.61)	21.81(9.68,47.94)	15.88(6.73,32.83)	-1.03(-1.08,-0.98)
Sao Tome and Principe	0.05(0.01,0.14)	0.07(0.01,0.22)	0.08(0.02,0.24)	0.09(0.02,0.25)	-0.23(-0.57,0.11)	1.45(0.57,3.47)	2.18(0.84,5.45)	2.30(0.91,5.51)	2.17(0.84,5.34)	-0.53(-0.81,-0.25)
Saudi Arabia	17.08(3.65,48.47)	39.64(8.31,115.91)	0.43(0.09,1.24)	0.40(0.09,1.14)	-0.32(-0.35,-0.30)	471.50(187.78,1110.82)	1350.15(545.35,3053.31)	10.10(3.97,23.92)	9.64(3.86,22.34)	-0.15(-0.19,-0.12)
Senegal	3.86(0.85,11.73)	7.64(1.59,21.87)	0.15(0.03,0.46)	0.12(0.03,0.36)	-0.66(-0.81,-0.50)	122.60(50.00,300.38)	224.79(85.26,564.58)	4.12(1.65,10.31)	3.14(1.19,7.68)	-0.91(-1.05,-0.77)
Serbia	59.36(13.10,171.79)	99.73(22.14,282.95)	0.67(0.15,1.92)	0.57(0.13,1.62)	-0.55(-0.91,-0.19)	1812.46(779.81,4216.31)	2677.08(1196.19,5978.39)	17.40(7.45,40.68)	15.78(7.12,35.32)	-0.35(-0.67,-0.03)
Seychelles	0.46(0.10,1.30)	0.62(0.14,1.81)	0.84(0.18,2.37)	0.67(0.15,1.97)	-0.82(-0.91,-0.72)	10.32(4.25,23.59)	15.64(6.54,35.72)	18.50(7.57,42.39)	15.13(6.38,34.48)	-0.75(-0.83,-0.66)
Sierra Leone	2.78(0.61,7.73)	3.50(0.80,10.26)	0.18(0.04,0.50)	0.13(0.03,0.37)	-1.12(-1.20,-1.04)	81.13(34.16,185.25)	108.83(43.48,260.47)	4.53(1.90,10.45)	3.31(1.30,8.15)	-1.03(-1.10,-0.97)
Singapore	7.04(1.60,20.60)	23.22(5.05,61.26)	0.47(0.11,1.33)	0.28(0.06,0.73)	-1.68(-1.79,-1.56)	185.39(76.36,426.34)	564.84(246.84,1180.87)	10.26(4.16,23.66)	6.60(2.86,13.86)	-1.42(-1.52,-1.32)
Slovakia	33.22(7.39,97.31)	46.18(10.20,130.25)	0.62(0.14,1.83)	0.48(0.11,1.36)	-0.69(-0.83,-0.54)	859.64(353.07,2002.11)	1211.31(508.96,2746.88)	14.91(6.09,35.67)	12.49(5.29,28.36)	-0.41(-0.57,-0.24)
Slovenia	12.49(2.85,35.73)	24.64(5.80,69.59)	0.53(0.12,1.51)	0.48(0.11,1.38)	-0.01(-0.19,0.16)	356.06(153.69,822.06)	631.83(264.82,1400.46)	14.54(6.26,33.86)	13.73(5.75,30.04)	0.17(-0.00,0.35)
Solomon Islands	0.60(0.13,1.82)	1.51(0.32,4.55)	0.76(0.17,2.22)	0.68(0.14,2.05)	-0.18(-0.36,-0.00)	19.74(8.15,47.44)	48.56(19.62,115.59)	18.58(7.79,44.80)	17.33(7.10,41.02)	-0.04(-0.24,0.16)
Somalia	3.97(0.83,12.32)	7.43(1.43,20.37)	0.36(0.08,1.10)	0.27(0.05,0.75)	-0.92(-0.99,-0.85)	116.99(45.25,290.38)	233.41(86.09,537.00)	7.76(2.88,19.28)	5.80(2.08,12.75)	-0.91(-1.00,-0.81)
South Africa	135.16(31.63,374.12)	109.02(24.24,330.34)	0.87(0.20,2.35)	0.32(0.07,0.98)	-3.44(-3.55,-3.32)	3268.54(1435.34,7213.19)	2993.23(1287.98,7008.50)	18.84(8.30,41.85)	7.40(3.07,17.37)	-3.14(-3.28,-3.01)
South Sudan	6.50(1.41,18.41)	7.32(1.52,21.70)	0.39(0.08,1.15)	0.34(0.07,1.00)	-0.42(-0.46,-0.38)	175.93(66.41,406.17)	192.63(77.37,470.63)	8.51(3.32,19.35)	6.96(2.76,16.56)	-0.68(-0.75,-0.61)
Spain	550.90(126.74,1503.32)	695.79(166.49,1868.41)	1.04(0.24,2.84)	0.55(0.13,1.48)	-2.15(-2.19,-2.11)	12313.50(5205.74,28102.81)	13722.94(5710.33,31275.91)	22.32(9.39,51.37)	12.62(5.39,28.29)	-1.86(-1.88,-1.83)
Sri Lanka	48.48(11.13,139.88)	70.99(15.99,206.49)	0.78(0.17,2.29)	0.33(0.07,0.96)	-2.98(-3.28,-2.68)	1293.64(561.41,2830.92)	1775.17(777.47,4149.45)	16.37(6.96,36.77)	7.28(3.19,17.10)	-2.84(-3.13,-2.56)
Sudan	47.21(11.29,137.41)	94.02(20.88,285.00)	0.83(0.19,2.38)	0.75(0.16,2.30)	-0.29(-0.31,-0.26)	1248.97(530.94,2921.77)	2257.81(934.58,5503.14)	17.68(7.25,41.57)	15.43(6.25,37.60)	-0.43(-0.48,-0.39)
Suriname	1.16(0.24,3.22)	2.00(0.41,6.12)	0.52(0.11,1.46)	0.35(0.07,1.07)	-1.70(-1.88,-1.52)	31.94(14.09,73.06)	54.24(22.58,125.14)	13.58(5.99,30.71)	8.90(3.70,20.62)	-1.73(-1.90,-1.56)
Sweden	189.73(40.85,535.36)	281.01(68.39,787.72)	1.10(0.24,3.11)	1.01(0.24,2.84)	-0.20(-0.35,-0.05)	4071.27(1752.22,9013.10)	5610.53(2484.22,12538.33)	24.37(10.55,53.33)	22.24(9.97,49.67)	-0.20(-0.31,-0.09)
Switzerland	138.39(31.98,401.08)	236.95(59.52,603.50)	1.20(0.28,3.49)	0.98(0.24,2.52)	-0.47(-0.53,-0.40)	2887.20(1215.54,6622.55)	4289.45(1788.23,9502.79)	25.76(10.98,58.84)	20.03(8.38,44.81)	-0.67(-0.73,-0.61)
Syrian Arab Republic	51.24(11.14,149.92)	73.55(15.74,207.15)	1.37(0.30,4.00)	1.00(0.22,2.88)	-1.17(-1.27,-1.08)	1205.24(517.81,2832.91)	1959.55(841.26,4428.48)	28.98(12.39,67.77)	20.19(8.13,45.95)	-1.33(-1.40,-1.26)
Taiwan (Province of Chi	83.17(19.58,233.70)	296.24(65.87,862.92)	0.85(0.19,2.25)	0.62(0.14,1.81)	-1.30(-1.40,-1.19)	2193.71(931.88,5134.23)	5485.41(2112.81,13408.39)	17.64(7.46,40.60)	12.11(4.69,29.43)	-1.46(-1.54,-1.38)
Tajikistan	16.58(3.78,50.37)	16.00(3.24,47.15)	0.75(0.17,2.25)	0.45(0.09,1.29)	-1.35(-1.56,-1.14)	387.90(169.86,921.60)	426.15(168.81,1006.80)	16.02(6.98,38.31)	9.57(3.81,22.31)	-1.40(-1.54,-1.27)
Thailand	247.57(52.98,693.45)	743.06(170.63,2054.71)	1.03(0.22,2.91)	0.67(0.15,1.85)	-1.74(-1.87,-1.61)	6147.74(2531.00,13958.82)	15621.96(6409.23,34864.13)	21.81(8.86,50.68)	14.06(5.79,31.32)	-1.60(-1.69,-1.52)
Timor-Leste	1.24(0.26,3.50)	4.21(0.98,12.48)	0.85(0.18,2.45)	0.78(0.17,2.23)	-0.41(-0.52,-0.30)	35.97(15.30,76.96)	112.33(49.37,255.03)	19.54(8.37,43.38)	16.68(7.01,38.15)	-0.65(-0.71,-0.58)
Togo	3.14(0.70,8.56)	5.80(1.33,17.11)	0.44(0.10,1.24)	0.26(0.06,0.75)	-1.74(-1.88,-1.60)	85.40(33.04,198.53)	173.57(68.21,419.80)	9.33(3.52,21.67)	5.81(2.23,13.99)	-1.57(-1.72,-1.42)
Tokelau	0.01(0.00,0.02)	0.01(0.00,0.02)	0.69(0.15,2.01)	0.54(0.11,1.60)	-0.92(-0.98,-0.85)	0.20(0.08,0.49)	0.19(0.07,0.46)	15.64(5.90,38.21)	12.68(4.88,31.10)	-0.83(-0.89,-0.77)
Tonga	0.45(0.10,1.21)	0.69(0.14,1.98)	1.18(0.28,3.13)	0.95(0.20,2.69)	-0.78(-0.88,-0.68)	11.38(4.41,25.39)	15.25(6.10,35.91)	25.04(9.69,55.53)	20.18(7.98,47.32)	-0.80(-0.88,-0.72)
Trinidad and Tobago	2.64(0.58,8.18)	5.37(1.16,16.85)	0.39(0.09,1.20)	0.29(0.06,0.91)	-0.95(-1.00,-0.89)	78.42(34.67,187.27)	145.91(62.71,345.90)	10.15(4.41,24.78)	7.55(3.28,17.68)	-1.00(-1.05,-0.95)
Tunisia	51.88(12.67,141.04)	132.41(28.27,382.18)	1.65(0.39,4.35)	1.22(0.26,3.43)	-1.11(-1.20,-1.03)	1348.60(603.82,3035.52)	2973.19(1229.13,6816.69)	34.76(15.34,77.07)	25.01(10.16,57.46)	-1.13(-1.17,-1.09)
Turkey	333.66(73.48,961.67)	605.75(141.27,1757.66)	1.30(0.28,3.81)	0.75(0.18,2.21)	-2.10(-2.40,-1.79)	8767.59(3748.34,19478.64)	15189.13(6484.62,34598.75)	29.94(12.83,68.27)	17.34(7.48,39.77)	-2.06(-2.27,-1.86)
Turkmenistan	7.11(1.52,20.02)	10.31(2.13,29.50)	0.53(0.11,1.47)	0.34(0.07,0.98)	-1.38(-1.60,-1.16)	196.65(85.01,435.75)	290.06(128.68,646.16)	12.25(5.28,26.86)	8.41(3.69,19.05)	-1.21(-1.32,-1.10)
Tuvalu	0.03(0.01,0.08)	0.05(0.01,0.13)	0.60(0.13,1.77)	0.60(0.13,1.69)	-0.09(-0.13,-0.04)	0.84(0.32,2.07)	1.34(0.56,3.17)	14.64(5.50,37.04)	14.26(5.87,34.21)	-0.15(-0.19,-0.11)
Uganda	13.07(2.78,36.20)	28.90(6.24,83.91)	0.38(0.08,1.07)	0.35(0.07,0.98)	-0.82(-1.07,-0.57)	324.02(132.32,729.34)	681.77(263.27,1642.04)	7.34(2.85,16.92)	6.53(2.56,15.41)	-0.83(-1.06,-0.61)
Ukraine	322.44(73.60,957.84)	335.56(72.41,991.68)	0.53(0.12,1.56)	0.42(0.09,1.23)	-0.81(-1.05,-0.57)	8730.07(3927.56,20308.47)	8324.27(3482.02,19318.96)	12.83(5.67,29.86)	10.39(4.37,24.23)	-0.71(-0.97,-0.45)
United Arab Emirates	1.55(0.34,4.36)	9.34(1.86,26.53)	0.84(0.19,2.34)	1.10(0.24,3.04)	1.18(0.99,1.36)	44.11(17.68,99.08)	373.87(149.50,806.75)	17.11(6.72,40.04)	20.89(8.21,49.29)	0.84(0.70,0.97)
United Kingdom	1418.90(329.41,4164.27)	1425.15(334.73,3853.53)	1.51(0.35,4.34)	0.89(0.21,2.44)	-1.86(-1.98,-1.75)	29827.51(12892.10,68462.17)	27070.11(11196.92,62977.37)	31.32(13.60,72.19)	18.18(7.59,42.28)	-1.95(-2.06,-1.84)
United Republic of Tanz	45.32(11.04,128.88)	86.16(20.38,237.83)	0.78(0.18,2.16)	0.52(0.12,1.40)	-1.62(-1.75,-1.49)	1176.41(483.67,2787.83)	2071.75(809.72,4804.58)	15.39(6.19,37.21)	10.43(3.90,24.24)	-1.51(-1.63,-1.39)
United States of America	5053.59(1203.00,13945.91)	7091.07(1720.98,19159.43)	1.49(0.35,4.13)	1.08(0.26,2.93)	-1.20(-1.27,-1.13)	115023.43(50585.52,268831.74)	148705.22(65330.84,336695.05)	34.69(15.28,80.53)	23.75(10.43,53.44)	-1.36(-1.42,-1.29)
United States Virgin Isla	0.20(0.04,0.56)	0.52(0.10,1.41)	0.33(0.07,0.94)	0.28(0.06,0.78)	-0.52(-0.61,-0.43)	5.95(2.36,13.19)	13.74(6.03,29.61)	8.20(3.21,18.36)	7.12(3.08,15.58)	-0.45(-0.50,-0.39)
Uruguay	16.49(3.92,47.72)	30.04(6.96,87.67)	0.43(0.10,1.23)	0.46(0.11,1.33)	0.24(0.15,0.33)	474.35(206.94,1070.38)	709.18(305.52,1650.05)	12.02(5.23,27.13)	12.14(5.19,28.04)	-0.03(-0.13,0.07)
Uzbekistan	23.56(5.36,68.92)	52.39(12.28,161.59)	0.23(0.05,0.68)	0.28(0.06,0.87)	0.71(0.51,0.92)	593.67(251.15,1407.76)	1571.17(663.23,3665.95)	5.47(2.30,13.03)	7.03(3.01,16.56)	0.90(0.62,1.18)
Vanuatu	0.17(0.04,0.48)	0.37(0.09,1.05)	0.46(0.10,1.31)	0.35(0.08,0.96)	-1.21(-1.33,-1.09)	5.30(2.17,12.28)	11.22(4.56,26.50)	11.03(4.64,25.95)	8.15(3.30,18.88)	-1.25(-1.37,-1.12)
Venezuela (Bolivarian R	49.49(10.91,135.95)	102.77(22.37,293.42)	0.67(0.15,1.81)	0.37(0.08,1.05)	-1.94(-1.99,-1.89)	1304.17(588.66,2905.21)	2624.22(1121.81,5991.74)	15.75(7.04,34.46)	9.09(3.87,20.64)	-1.82(-1.88,-1.77)
Viet Nam	323.06(72.66,902.01)	601.22(132.85,1640.65)	0.99(0.22,2.81)	0.76(0.17,2.06)	-1.37(-1.59,-1.16)	8019.32(3341.27,18248.85)	15230.13(6108.96,36090.72)	22.23(9.31,50.31)	17.01(6.99,40.53)	-1.29(-1.46,-1.12)
Yemen	29.06(6.83,85.98)	85.51(19.27,247.80)	1.01(0.23,3.04)	1.00(0.23,2.86)	-0.05(-0.08,-0.02)	873.73(360.84,1993.92)	2292.02(967.44,5234.49)	23.61(9.81,54.83)	21.80(9.17,50.42)	-0.31(-0.34,-0.27)
Zambia	9.85(2.18,27.14)	18.03(3.90,53.49)	0.65(0.14,1.81)	0.52(0.11,1.52)	-0.80(-0.87,-0.72)	259.78(102.91,586.10)	451.87(177.62,1068.44)	13.19(5.12,30.26)	10.04(3.72,24.33)	-1.05(-1.10,-0.99)
Zimbabwe	21.62(5.02,63.68)	25.00(5.49,73.48)	0.99(0.22,2.93)	0.76(0.17,2.21)	-0.75(-0.95,-0.54)	540.20(212.68,1242.68)	677.72(256.77,1584.95)	18.99(7.24,45.22)	15.09(5.55,36.43)	-0.72(-0.87,-0.57)

Table S2 The number of Mortality and DALYs of Multiple sclerosis, along with corresponding ASR and temporal trends.

Characteristics	Mortality(95% uncertainty interval)					DALYs(95% uncertainty interval)				
	Cases,1990	Cases,2021	ASMR,1990	ASMR,2021	EAPC 1990-2021	Cases,1990	Cases,2021	ASDR,1990	ASDR,2021	EAPC 1990-2021
Global	1677.74(1449.37,1898.06)	1821.73(1559.78,2098.24)	0.04(0.03,0.04)	0.02(0.02,0.02)	-2.10(-2.21,-1.99)	105344.85(88079.16,125490.91)	111100.33(91036.28,134377.57)	2.36(1.97,2.81)	1.28(1.05,1.55)	-2.06(-2.14,-1.98)
SDI level										
High SDI	1184.34(1028.76,1343.16)	1280.86(1085.17,1488.87)	0.12(0.10,0.13)	0.07(0.06,0.08)	-1.45(-1.51,-1.38)	73095.09(60851.18,86861.79)	71197.97(57945.62,86459.79)	7.26(6.05,8.64)	4.60(3.73,5.62)	-1.50(-1.54,-1.45)
High-middle SDI	425.38(364.75,481.35)	374.99(317.24,435.81)	0.04(0.03,0.05)	0.02(0.02,0.02)	-2.71(-2.93,-2.48)	25322.96(21349.27,29953.81)	24737.12(20174.53,29871.72)	2.39(2.02,2.83)	1.39(1.13,1.69)	-2.08(-2.24,-1.93)
Low SDI	1.70(0.44,3.01)	6.88(3.06,11.05)	0.00(0.00,0.00)	0.00(0.00,0.00)	1.68(1.56,1.80)	358.80(246.47,516.43)	931.30(662.97,1254.25)	0.12(0.08,0.17)	0.13(0.09,0.18)	0.25(0.23,0.27)
Low-middle SDI	10.51(5.80,14.96)	31.67(20.87,42.44)	0.00(0.00,0.00)	0.00(0.00,0.00)	0.84(0.68,0.99)	1790.51(1295.08,2500.13)	4159.13(3101.75,5603.81)	0.23(0.16,0.31)	0.24(0.18,0.33)	0.22(0.21,0.23)
Middle SDI	50.53(37.73,61.97)	124.22(101.85,150.89)	0.00(0.00,0.01)	0.00(0.00,0.01)	-0.21(-0.44,0.02)	4511.17(3520.99,5846.01)	9908.47(7932.58,12471.43)	0.35(0.27,0.45)	0.35(0.28,0.43)	-0.09(-0.15,-0.04)
GBD Region										
Andean Latin America	0.72(0.53,0.94)	2.49(1.74,3.44)	0.00(0.00,0.00)	0.00(0.00,0.01)	1.56(1.06,2.07)	40.18(30.81,51.84)	130.13(98.00,171.02)	0.16(0.13,0.21)	0.20(0.15,0.27)	1.17(0.88,1.47)
Australasia	13.10(10.99,15.52)	17.65(14.26,21.78)	0.06(0.05,0.07)	0.04(0.03,0.05)	1.48(1.18,1.79)	777.45(627.82,968.36)	1166.38(890.60,1518.43)	3.51(2.84,4.36)	2.94(2.26,3.84)	-0.51(-0.82,-0.19)
Caribbean	5.74(4.75,6.76)	10.24(7.93,12.82)	0.02(0.02,0.02)	0.02(0.01,0.02)	0.82(0.69,0.95)	292.35(237.39,355.19)	471.46(370.89,591.07)	1.04(0.84,1.26)	0.89(0.70,1.11)	-0.56(-0.68,-0.43)
Central Asia	8.67(7.16,10.51)	9.47(6.84,13.48)	0.02(0.01,0.02)	0.01(0.01,0.02)	0.63(0.52,0.73)	745.04(589.03,948.40)	1079.24(814.31,1447.64)	1.43(1.14,1.82)	1.12(0.85,1.51)	-0.55(-0.67,-0.43)
Central Europe	282.90(244.44,322.02)	142.34(118.41,170.09)	0.19(0.17,0.22)	0.08(0.07,0.10)	0.60(0.52,0.69)	13947.43(11957.75,16022.35)	7330.45(6046.66,8760.41)	9.75(8.36,11.23)	4.52(3.71,5.42)	-2.64(-2.71,-2.58)
Central Latin America	7.68(6.47,8.86)	23.19(18.74,28.20)	0.01(0.01,0.01)	0.01(0.01,0.01)	0.57(0.51,0.62)	425.22(354.06,512.03)	1086.06(885.45,1316.28)	0.40(0.33,0.48)	0.41(0.33,0.49)	-0.13(-0.47,0.21)
Central Sub-Saharan Africa	0.10(0.03,0.17)	0.41(0.16,0.71)	0.00(0.00,0.00)	0.00(0.00,0.00)	0.35(-0.09,0.78)	15.05(9.98,22.69)	47.71(31.36,68.68)	0.05(0.03,0.07)	0.05(0.04,0.08)	0.59(0.33,0.84)
East Asia	3.79(2.05,5.72)	10.07(7.41,13.44)	0.00(0.00,0.00)	0.00(0.00,0.00)	0.34(0.15,0.54)	856.60(587.18,1227.05)	1908.08(1359.99,2654.05)	0.08(0.05,0.11)	0.09(0.07,0.13)	0.51(0.35,0.67)
Eastern Europe	187.60(161.30,217.42)	139.44(115.19,164.64)	0.07(0.06,0.08)	0.05(0.04,0.06)	0.15(-0.32,0.63)	9863.05(8454.12,11411.82)	7941.47(6594.50,9397.03)	3.87(3.32,4.47)	2.89(2.40,3.42)	-1.76(-2.19,-1.34)
Eastern Sub-Saharan Africa	0.35(0.08,0.59)	1.10(0.37,1.83)	0.00(0.00,0.00)	0.00(0.00,0.00)	-0.20(-0.32,-0.08)	56.17(37.78,80.41)	131.47(85.25,188.87)	0.05(0.04,0.08)	0.05(0.03,0.07)	-0.30(-0.43,-0.16)
High-income Asia Pacific	10.47(8.93,12.05)	9.37(7.72,11.09)	0.01(0.00,0.01)	0.00(0.00,0.00)	-0.26(-0.38,-0.15)	1160.06(863.36,1540.29)	949.22(697.54,1262.02)	0.56(0.42,0.75)	0.35(0.26,0.47)	-1.64(-1.77,-1.51)
High-income North America	433.14(370.03,496.51)	625.40(520.46,750.37)	0.14(0.12,0.16)	0.11(0.09,0.13)	-0.80(-1.15,-0.45)	32566.25(26399.59,39713.06)	34728.37(28024.85,42613.43)	10.52(8.54,12.81)	6.92(5.56,8.51)	-1.45(-1.53,-1.37)
North Africa and Middle East	27.06(13.38,40.11)	93.99(69.62,120.12)	0.01(0.01,0.02)	0.02(0.01,0.02)	-0.83(-0.92,-0.74)	3300.28(2391.17,4364.37)	9578.06(7458.40,12317.00)	1.48(1.07,1.96)	1.56(1.22,2.01)	0.26(0.22,0.29)
Oceania	0.00(0.00,0.00)	0.00(0.00,0.00)	0.00(0.00,0.00)	0.00(0.00,0.00)	-1.09(-1.31,-0.87)	1.67(1.01,2.53)	4.33(2.61,6.78)	0.04(0.02,0.05)	0.04(0.02,0.06)	-0.03(-0.11,0.06)
South Asia	5.30(1.56,9.21)	15.59(7.71,23.55)	0.00(0.00,0.00)	0.00(0.00,0.00)	-1.21(-1.42,-1.00)	1380.41(965.72,1960.64)	2598.35(1870.38,3584.27)	0.18(0.12,0.25)	0.15(0.11,0.21)	-0.61(-0.63,-0.59)
Southeast Asia	1.42(0.82,1.93)	3.95(2.95,4.89)	0.00(0.00,0.00)	0.00(0.00,0.00)	-1.83(-2.09,-1.56)	240.95(171.36,339.91)	535.79(389.35,740.41)	0.07(0.05,0.10)	0.07(0.05,0.10)	-0.06(-0.11,-0.01)
Southern Latin America	19.29(16.28,22.37)	15.32(12.47,18.29)	0.04(0.03,0.05)	0.02(0.02,0.02)	-1.96(-2.07,-1.85)	1171.42(960.71,1415.58)	1120.32(875.81,1383.54)	2.50(2.05,3.02)	1.43(1.12,1.78)	-1.88(-2.10,-1.67)
Southern Sub-Saharan Africa	2.88(1.93,3.80)	3.63(2.65,4.76)	0.01(0.01,0.01)	0.01(0.00,0.01)	-1.97(-2.48,-1.46)	175.56(132.87,227.39)	210.60(160.43,271.49)	0.51(0.38,0.66)	0.29(0.22,0.37)	-2.01(-2.11,-1.92)
Tropical Latin America	16.42(13.93,18.85)	31.32(25.69,38.03)	0.02(0.01,0.02)	0.01(0.01,0.01)	-2.35(-2.85,-1.85)	1440.99(1110.03,1835.56)	2369.51(1810.72,3072.66)	1.28(0.99,1.63)	0.89(0.68,1.15)	-1.71(-1.93,-1.50)
Western Europe	651.12(561.94,740.53)	666.76(565.12,777.43)	0.13(0.11,0.15)	0.09(0.08,0.11)	-2.52(-2.86,-2.19)	36839.05(30732.90,43450.30)	37580.89(30142.46,45203.94)	7.93(6.62,9.41)	6.04(4.82,7.30)	-0.80(-0.83,-0.76)
Western Sub-Saharan Africa	0.00(0.00,0.00)	0.01(0.00,0.01)	0.00(0.00,0.00)	0.00(0.00,0.00)	-3.00(-3.08,-2.91)	49.67(32.40,73.18)	132.43(86.62,194.76)	0.04(0.03,0.06)	0.04(0.03,0.06)	0.12(0.06,0.18)
National level										
Afghanistan	0.25(0.05,0.53)	1.38(0.50,2.73)	0.00(0.00,0.01)	0.01(0.00,0.02)	3.29(2.85,3.73)	30.35(17.95,47.67)	146.37(85.91,219.45)	0.42(0.25,0.66)	0.83(0.50,1.23)	2.60(2.30,2.89)
Albania	5.86(3.53,8.41)	6.87(3.58,12.02)	0.26(0.16,0.37)	0.18(0.09,0.30)	-1.24(-1.52,-0.95)	304.39(213.50,397.86)	353.97(245.56,508.71)	12.39(8.68,16.31)	9.71(6.75,13.92)	-0.66(-0.80,-0.51)
Algeria	1.20(0.45,2.15)	7.13(3.58,12.04)	0.01(0.00,0.02)	0.02(0.01,0.03)	1.87(1.60,2.14)	163.58(110.76,232.88)	691.62(485.35,960.95)	1.10(0.74,1.56)	1.56(1.10,2.15)	1.08(1.05,1.11)
American Samoa	0.00(0.00,0.00)	0.00(0.00,0.00)	0.00(0.00,0.00)	0.00(0.00,0.00)	0.42(0.35,0.49)	0.02(0.01,0.03)	0.03(0.02,0.05)	0.06(0.03,0.09)	0.06(0.03,0.09)	0.02(-0.03,0.06)
Andorra	0.07(0.04,0.11)	0.12(0.06,0.19)	0.11(0.06,0.18)	0.08(0.04,0.13)	-0.43(-0.62,-0.24)	4.26(2.83,6.17)	8.12(5.08,11.82)	6.82(4.56,9.91)	6.00(3.72,8.67)	-0.15(-0.25,-0.05)
Angola	0.04(0.01,0.07)	0.21(0.07,0.38)	0.00(0.00,0.00)	0.00(0.00,0.00)	2.08(1.84,2.32)	5.77(3.73,8.42)	23.02(14.38,34.97)	0.09(0.06,0.14)	0.12(0.07,0.18)	0.94(0.67,1.21)
Antigua and Barbuda	0.00(0.00,0.01)	0.01(0.01,0.02)	0.01(0.01,0.01)	0.01(0.01,0.01)	0.75(0.48,1.02)	0.24(0.17,0.33)	0.65(0.45,0.91)	0.49(0.35,0.67)	0.57(0.39,0.80)	0.71(0.56,0.85)
Argentina	14.87(12.43,17.54)	11.66(9.36,14.16)	0.05(0.04,0.05)	0.02(0.02,0.03)	-2.31(-2.69,-1.94)	889.95(723.79,1081.84)	820.41(640.10,1022.36)	2.78(2.27,3.39)	1.61(1.25,2.01)	-1.83(-2.09,-1.57)
Armenia	0.57(0.45,0.71)	0.82(0.62,1.08)	0.02(0.02,0.03)	0.02(0.02,0.03)	1.79(0.77,2.82)	44.05(32.92,57.26)	64.08(46.95,84.60)	1.45(1.09,1.88)	1.62(1.18,2.14)	1.19(0.82,1.55)
Australia	10.40(8.67,12.31)	14.58(11.52,18.51)	0.06(0.05,0.07)	0.04(0.03,0.05)	-0.97(-1.18,-0.76)	642.28(511.56,798.58)	1024.73(768.16,1344.94)	3.46(2.76,4.30)	3.11(2.32,4.09)	-0.24(-0.59,0.12)
Austria	9.27(7.84,10.91)	18.28(15.00,21.66)	0.10(0.08,0.11)	0.13(0.10,0.15)	1.67(1.43,1.91)	531.21(434.99,643.79)	1061.01(836.37,1298.22)	5.79(4.74,7.06)	8.26(6.51,10.15)	1.60(1.36,1.84)
Azerbaijan	0.57(0.36,0.83)	1.10(0.65,1.77)	0.01(0.01,0.02)	0.01(0.01,0.02)	1.20(0.72,1.68)	50.58(36.79,69.41)	119.37(84.97,163.55)	0.89(0.65,1.20)	0.99(0.72,1.33)	0.76(0.58,0.95)
Bahamas	0.04(0.03,0.05)	0.11(0.07,0.15)	0.02(0.01,0.02)	0.02(0.02,0.03)	1.34(1.06,1.62)	1.69(1.31,2.21)	4.94(3.35,7.05)	0.86(0.67,1.12)	1.07(0.72,1.52)	1.23(1.00,1.46)
Bahrain	0.00(0.00,0.00)	0.11(0.07,0.18)	0.00(0.00,0.00)	0.01(0.01,0.01)	4.88(3.42,6.36)	2.84(1.68,4.58)	19.44(12.41,29.22)	0.75(0.44,1.18)	1.10(0.70,1.66)	1.26(1.12,1.41)
Bangladesh	0.40(0.06,0.90)	1.60(0.39,3.19)	0.00(0.00,0.00)	0.00(0.00,0.00)	1.20(0.98,1.43)	109.46(74.23,161.31)	289.38(194.82,414.05)	0.18(0.12,0.26)	0.18(0.12,0.26)	0.21(-0.03,0.45)
Barbados	0.04(0.03,0.04)	0.06(0.04,0.09)	0.01(0.01,0.02)	0.01(0.01,0.02)	0.08(-0.16,0.31)	1.68(1.34,2.10)	2.66(1.87,3.81)	0.70(0.55,0.88)	0.66(0.47,0.95)	0.03(-0.21,0.28)
Belarus	8.27(6.62,10.16)	6.61(4.90,8.67)	0.07(0.06,0.09)	0.05(0.04,0.06)	-1.60(-2.00,-1.21)	395.53(316.54,484.58)	315.84(235.07,399.99)	3.42(2.71,4.19)	2.49(1.87,3.17)	-1.48(-1.83,-1.14)
Belgium	20.23(16.73,23.57)	22.43(18.49,27.34)	0.15(0.13,0.17)	0.13(0.10,0.15)	-0.50(-0.69,-0.30)	1106.21(909.31,1313.49)	1224.13(960.94,1515.80)	8.90(7.32,10.59)	7.74(6.09,9.60)	-0.40(-0.54,-0.25)
Belize	0.00(0.00,0.00)	0.02(0.01,0.02)	0.00(0.00,0.00)	0.00(0.00,0.01)	1.79(1.47,2.12)	0.23(0.18,0.30)	1.09(0.80,1.48)	0.23(0.17,0.28)	0.28(0.21,0.39)	1.12(0.96,1.28)
Benin	0.00(0.00,0.00)	0.00(0.00,0.00)	0.00(0.00,0.00)	0.00(0.00,0.00)	-1.05(-1.19,-0.91)	1.19(0.73,1.83)	2.49(1.46,3.90)	0.05(0.03,0.07)	0.03(0.02,0.05)	-1.44(-1.69,-1.19)
Bermuda	0.01(0.01,0.02)	0.02(0.01,0.02)	0.02(0.01,0.02)	0.02(0.01,0.02)	-0.23(-0.51,0.06)	0.62(0.47,0.84)	0.91(0.65,1.25)	0.92(0.69,1.24)	0.93(0.67,1.28)	0.12(-0.06,0.30)
Bhutan	0.00(0.00,0.00)	0.00(0.00,0.01)	0.00(0.00,0.00)	0.00(0.00,0.00)	1.70(1.63,1.77)	0.46(0.28,0.76)	0.96(0.59,1.47)	0.13(0.08,0.20)	0.13(0.08,0.20)	0.08(-0.03,0.18)
Bolivia (Plurinational State of)	0.14(0.06,0.26)	0.48(0.23,0.84)	0.00(0.00,0.01)	0.00(0.00,0.01)	0.91(0.77,1.05)	8.47(5.16,12.87)	26.67(16.75,39.68)	0.22(0.13,0.33)	0.26(0.16,0.38)	0.87(0.72,1.02)
Bosnia and Herzegovina	4.90(3.29,6.76)	3.32(2.09,4.97)	0.10(0.07,0.14)	0.06(0.04,0.10)	-1.50(-1.76,-1.24)	251.21(185.50,336.00)	177.39(125.47,239.22)	5.16(3.82,6.91)	3.76(2.65,5.07)	-0.93(-1.18,-0.68)
Botswana	0.01(0.00,0.01)	0.02(0.01,0.03)	0.00(0.00,0.00)	0.00(0.00,0.00)	-0.42(-0.64,-0.21)	1.38(0.92,2.02)	3.35(2.28,4.77)	0.18(0.12,0.27)	0.15(0.10,0.22)	-0.64(-0.71,-0.58)
Brazil	16.29(13.80,18.71)	30.80(25.28,37.48)	0.02(0.01,0.02)	0.01(0.01,0.01)	-2.01(-2.52,-1.50)	1427.23(1099.90,1817.01)	2334.99(1781.47,3034.24)	1.30(1.01,1.65)	0.90(0.68,1.16)	-1.73(-1.95,-1.51)
Brunei Darussalam	0.01(0.00,0.01)	0.01(0.00,0.02)	0.00(0.00,0.01)	0.00(0.00,0.01)	-1.34(-1.57,-1.10)	0.59(0.39,1.00)	1.04(0.59,1.72)	0.33(0.21,0.54)	0.20(0.11,0.33)	-1.50(-1.65,-1.36)
Bulgaria	19.80(16.19,23.67)	11.01(8.20,14.00)	0.18(0.15,0.21)	0.11(0.08,0.14)	-1.99(-2.25,-1.72)	1090.50(890.31,1303.69)	631.13(500.24,784.32)	10.06(8.24,11.99)	6.59(5.25,8.16)	-1.66(-1.86,-1.45)
Burkina Faso	0.00(0.00,0.00)	0.00(0.00,0.00)	0.00(0.00,0.00)	0.00(0.00,0.00)	0.49(0.05,0.92)	2.22(1.40,3.31)	5.38(3.33,8.29)	0.04(0.03,0.06)	0.04(0.02,0.06)	-0.15(-0.35,0.05)
Burundi	0.01(0.00,0.02)	0.02(0.00,0.04)	0.00(0.00,0.00)	0.00(0.00,0.00)	-1.18(-1.57,-0.79)	1.51(0.95,2.25)	2.40(1.42,3.88)	0.05(0.03,0.07)	0.03(0.02,0.05)	-1.42(-1.77,-1.06)
Cabo Verde	0.00(0.00,0.00)	0.00(0.00,0.00)	0.00(0.00,0.00)	0.00(0.00,0.00)	-0.03(-0.22,0.16)	0.14(0.09,0.21)	0.30(0.19,0.44)	0.07(0.05,0.11)	0.05(0.03,0.08)	-1.02(-1.13,-0.91)
Cambodia	0.01(0.00,0.02)	0.05(0.02,0.10)	0.00(0.00,0.00)	0.00(0.00,0.00)	2.28(1.88,2.68)	3.36(2.27,4.91)	11.15(7.43,16.74)	0.05(0.04,0.08)	0.07(0.05,0.11)	0.71(0.51,0.92)
Cameroon	0.00(0.00,0.00)	0.00(0.00,0.00)	0.00(0.00,0.00)	0.00(0.00,0.00)	-0.37(-0.46,-0.28)	3.69(2.23,5.55)	9.62(6.00,13.76)	0.06(0.04,0.09)	0.05(0.03,0.07)	-0.74(-0.86,-0.61)
Canada	49.20(41.47,57.18)	66.16(53.30,82.15)	0.16(0.13,0.18)	0.11(0.09,0.13)	-1.46(-1.66,-1.26)	3538.36(2843.31,4320.13)	4212.67(3223.00,5354.79)	11.42(9.21,13.93)	8.00(6.07,10.21)	-1.40(-1.50,-1.29)
Central African Republic	0.01(0.00,0.01)	0.01(0.00,0.03)	0.00(0.00,0.00)	0.00(0.00,0.00)	-0.08(-0.27,0.12)	0.89(0.58,1.34)	1.75(1.10,2.74)	0.05(0.03,0.08)	0.05(0.03,0.07)	-0.49(-0.73,-0.25)
Chad	0.00(0.00,0.00)	0.00(0.00,0.00)	0.00(0.00,0.00)	0.00(0.00,0.00)	0.44(0.30,0.57)	1.89(1.12,2.96)	4.38(2.50,7.09)	0.05(0.03,0.08)	0.05(0.03,0.08)	-0.53(-0.60,-0.46)
Chile	2.20(1.81,2.60)	1.76(1.41,2.09)	0.02(0.02,0.02)	0.01(0.01,0.01)	-3.16(-3.38,-2.93)	168.69(130.81,217.07)	191.45(139.20,246.91)	1.44(1.13,1.84)	0.85(0.62,1.10)	-1.80(-1.91,-1.68)
China	3.60(1.90,5.48)	9.82(7.24,13.16)	0.00(0.00,0.00)	0.00(0.00,0.00)	0.76(0.30,1.23)	815.79(560.91,1171.28)	1830.10(1302.52,2543.04)	0.08(0.05,0.11)	0.09(0.07,0.13)	0.59(0.43,0.76)
Colombia	1.38(1.10,1.70)	3.21(2.45,4.26)	0.01(0.01,0.01)	0.01(0.00,0.01)	-0.49(-1.00,0.02)	73.09(57.35,90.51)	154.01(117.99,203.58)	0.32(0.25,0.39)	0.28(0.22,0.37)	-0.53(-0.93,-0.12)
Comoros	0.00(0.00,0.00)	0.00(0.00,0.01)	0.00(0.00,0.00)	0.00(0.00,0.00)	0.48(0.32,0.65)	0.18(0.10,0.28)	0.40(0.23,0.63)	0.07(0.04,0.11)	0.06(0.04,0.10)	-0.38(-0.53,-0.24)
Congo	0.01(0.00,0.01)	0.03(0.02,0.05)	0.00(0.00,0.00)	0.00(0.00,0.00)	1.69(1.46,1.92)	0.68(0.46,0.97)	3.02(2.07,4.44)	0.05(0.03,0.07)	0.07(0.05,0.10)	1.39(1.16,1.63)
Cook Islands	0.00(0.00,0.00)	0.00(0.00,0.00)	0.00(0.00,0.00)	0.00(0.00,0.00)	0.00(-0.09,0.09)	0.01(0.01,0.02)	0.01(0.01,0.02)	0.07(0.04,0.11)	0.06(0.04,0.10)	-0.51(-0.62,-0.40)
Costa Rica	0.15(0.13,0.19)	0.71(0.55,0.89)	0.01(0.01,0.01)	0.01(0.01,0.02)	1.45(1.02,1.88)	8.61(6.86,10.58)	32.79(26.01,41.45)	0.41(0.33,0.51)	0.60(0.47,0.76)	1.14(0.81,1.47)
Côte d'Ivoire	0.00(0.00,0.00)	0.00(0.00,0.00)	0.00(0.00,0.00)	0.00(0.00,0.00)	-1.10(-1.45,-0.75)	5.08(3.17,7.78)	11.40(7.05,17.40)	0.07(0.05,0.11)	0.06(0.04,0.09)	-1.16(-1.42,-0.91)
Croatia	9.55(7.82,11.29)	4.94(3.79,6.21)	0.15(0.13,0.18)	0.07(0.06,0.09)	-2.01(-2.18,-1.85)	427.03(352.16,498.17)	242.93(190.64,299.34)	7.04(5.79,8.24)	4.07(3.21,5.05)	-1.39(-1.54,-1.24)
Cuba	3.99(3.24,4.74)	7.05(5.29,9.12)	0.04(0.03,0.05)	0.04(0.03,0.05)	-0.02(-0.13,0.08)	196.22(158.43,236.42)	303.39(228.25,391.65)	1.91(1.54,2.29)	1.79(1.36,2.30)	-0.34(-0.45,-0.22)
Cyprus	0.63(0.27,1.10)	1.40(0.86,2.07)	0.08(0.03,0.14)	0.07(0.05,0.11)	0.14(0.01,0.27)	33.59(20.50,52.08)	84.57(61.29,115.61)	4.16(2.55,6.47)	4.60(3.33,6.27)	0.66(0.52,0.80)
Czechia	27.78(22.73,33.69)	14.25(10.94,18.28)	0.22(0.18,0.27)	0.09(0.07,0.11)	-2.98(-3.21,-2.76)	1257.34(1046.06,1524.08)	694.56(539.54,874.80)	10.15(8.47,12.33)	4.57(3.55,5.73)	-2.61(-2.80,-2.42)
Democratic People's Republic of Korea	0.06(0.02,0.10)	0.12(0.04,0.22)	0.00(0.00,0.00)	0.00(0.00,0.00)	0.26(0.20,0.31)	19.36(13.05,28.79)	32.56(21.23,47.85)	0.10(0.06,0.14)	0.10(0.06,0.14)	-0.09(-0.15,-0.04)
Democratic Republic of the Congo	0.05(0.01,0.09)	0.14(0.04,0.27)	0.00(0.00,0.00)	0.00(0.00,0.00)	0.49(0.09,0.90)	7.26(4.54,11.48)	18.32(11.73,27.05)	0.03(0.02,0.05)	0.03(0.02,0.05)	-0.14(-0.39,0.11)
Denmark	23.79(19.69,27.73)	21.22(17.01,25.63)	0.35(0.29,0.40)	0.22(0.17,0.26)	-1.54(-1.74,-1.33)	1161.01(964.78,1367.54)	970.25(772.92,1184.75)	18.08(15.01,21.38)	11.61(9.14,14.28)	-1.39(-1.54,-1.24)
Djibouti	0.00(0.00,0.00)	0.01(0.00,0.02)	0.00(0.00,0.00)	0.00(0.00,0.00)	0.63(0.50,0.76)	0.27(0.16,0.42)	1.10(0.62,1.80)	0.11(0.07,0.17)	0.10(0.06,0.17)	-0.34(-0.39,-0.30)
Dominica	0.00(0.00,0.00)	0.00(0.00,0.01)	0.00(0.00,0.01)	0.01(0.00,0.01)	1.35(1.07,1.62)	0.13(0.10,0.18)	0.24(0.15,0.35)	0.25(0.18,0.33)	0.29(0.19,0.44)	0.86(0.63,1.08)
Dominican Republic	0.22(0.15,0.31)	0.57(0.35,0.88)	0.01(0.00,0.01)	0.01(0.00,0.01)	0.64(0.24,1.05)	17.54(12.78,24.06)	39.39(27.11,56.38)	0.38(0.27,0.51)	0.36(0.25,0.52)	0.11(-0.18,0.39)
Ecuador	0.38(0.31,0.46)	0.91(0.65,1.22)	0.01(0.01,0.01)	0.01(0.00,0.01)	0.69(-0.17,1.56)	18.44(14.66,22.91)	42.33(31.12,55.18)	0.29(0.23,0.36)	0.25(0.18,0.32)	0.33(-0.27,0.93)
Egypt	0.22(0.12,0.35)	1.18(0.67,1.82)	0.00(0.00,0.00)	0.00(0.00,0.00)	3.17(2.80,3.55)	161.97(111.48,236.06)	672.39(448.57,969.51)	0.42(0.29,0.61)	0.75(0.50,1.07)	1.96(1.91,2.02)
El Salvador	0.07(0.05,0.10)	0.33(0.22,0.47)	0.00(0.00,0.00)	0.01(0.00,0.01)	3.58(3.13,4.03)	4.74(3.50,6.42)	17.40(12.28,24.33)	0.14(0.10,0.19)	0.29(0.20,0.41)	2.89(2.56,3.23)
Equatorial Guinea	0.00(0.00,0.00)	0.01(0.00,0.01)	0.00(0.00,0.00)	0.00(0.00,0.00)	2.12(2.07,2.17)	0.12(0.07,0.20)	0.56(0.34,0.88)	0.05(0.03,0.07)	0.06(0.04,0.09)	1.04(0.98,1.11)
Eritrea	0.01(0.00,0.02)	0.02(0.01,0.05)	0.00(0.00,0.00)	0.00(0.00,0.00)	0.73(0.57,0.89)	1.17(0.63,2.11)	2.80(1.59,4.96)	0.06(0.03,0.10)	0.06(0.03,0.10)	-0.12(-0.21,-0.03)
Estonia	2.96(2.43,3.55)	0.97(0.76,1.19)	0.16(0.13,0.19)	0.05(0.04,0.06)	-4.86(-5.31,-4.41)	133.85(110.53,159.50)	45.62(36.05,55.12)	7.45(6.15,8.88)	2.68(2.12,3.23)	-4.47(-4.87,-4.06)
Eswatini	0.00(0.00,0.00)	0.00(0.00,0.01)	0.00(0.00,0.00)	0.00(0.00,0.00)	0.20(-0.01,0.42)	0.28(0.19,0.38)	0.44(0.29,0.67)	0.07(0.05,0.10)	0.06(0.04,0.08)	-0.83(-0.92,-0.74)
Ethiopia	0.05(0.01,0.10)	0.11(0.02,0.20)	0.00(0.00,0.00)	0.00(0.00,0.00)	0.37(0.07,0.68)	7.77(4.90,11.53)	15.50(9.50,22.69)	0.03(0.02,0.04)	0.02(0.01,0.03)	-0.28(-0.50,-0.05)
Fiji	0.00(0.00,0.00)	0.00(0.00,0.00)	0.00(0.00,0.00)	0.00(0.00,0.00)	-0.49(-0.57,-0.40)	0.26(0.17,0.41)	0.37(0.24,0.55)	0.04(0.03,0.07)	0.04(0.03,0.06)	-0.47(-0.65,-0.30)
Finland	6.26(5.21,7.43)	7.16(5.70,8.77)	0.10(0.08,0.12)	0.08(0.07,0.10)	-0.06(-0.32,0.20)	379.25(307.08,463.80)	387.20(304.40,479.18)	6.12(4.96,7.50)	5.18(4.09,6.39)	-0.16(-0.34,0.02)
France	58.16(47.98,69.04)	68.33(54.48,83.82)	0.08(0.07,0.10)	0.07(0.05,0.08)	-0.40(-0.51,-0.28)	3669.26(2949.03,4485.83)	4553.58(3595.97,5677.90)	5.51(4.40,6.75)	5.29(4.11,6.62)	0.05(-0.07,0.17)
Gabon	0.00(0.00,0.00)	0.01(0.01,0.02)	0.00(0.00,0.00)	0.00(0.00,0.00)	1.71(1.52,1.90)	0.32(0.21,0.46)	1.05(0.69,1.53)	0.05(0.03,0.07)	0.07(0.05,0.10)	1.17(1.05,1.28)
Gambia	0.00(0.00,0.00)	0.00(0.00,0.00)	0.00(0.00,0.00)	0.00(0.00,0.00)	-0.82(-1.00,-0.64)	0.51(0.33,0.76)	0.96(0.61,1.40)	0.10(0.06,0.14)	0.06(0.04,0.09)	-1.38(-1.43,-1.34)
Georgia	0.44(0.34,0.54)	0.52(0.41,0.65)	0.01(0.01,0.01)	0.01(0.01,0.01)	3.45(1.93,4.99)	53.17(37.43,71.97)	57.66(41.16,76.44)	0.87(0.62,1.15)	1.16(0.83,1.58)	1.91(1.53,2.28)
Germany	164.81(137.72,194.50)	160.23(129.30,194.63)	0.15(0.13,0.18)	0.11(0.09,0.13)	-0.72(-0.89,-0.54)	9171.47(7433.61,11049.60)	8447.37(6588.05,10375.79)	9.00(7.26,10.87)	6.69(5.16,8.31)	-0.82(-0.90,-0.75)
Ghana	0.00(0.00,0.00)	0.00(0.00,0.00)	0.00(0.00,0.00)	0.00(0.00,0.00)	2.43(2.03,2.84)	3.92(2.54,5.80)	18.54(11.50,28.53)	0.04(0.03,0.06)	0.08(0.05,0.11)	2.42(2.10,2.74)
Greece	12.50(10.52,14.63)	19.45(15.83,23.59)	0.09(0.08,0.11)	0.12(0.09,0.14)	0.92(0.85,0.99)	622.57(516.28,748.48)	872.45(699.08,1047.18)	4.88(4.03,5.89)	5.83(4.67,7.03)	0.64(0.59,0.70)
Greenland	0.01(0.00,0.01)	0.03(0.02,0.05)	0.02(0.01,0.02)	0.04(0.02,0.06)	6.09(4.34,7.87)	1.89(1.24,2.61)	2.65(1.83,3.63)	3.41(2.28,4.72)	3.71(2.59,5.06)	1.06(0.67,1.44)
Grenada	0.01(0.01,0.01)	0.02(0.01,0.02)	0.01(0.01,0.02)	0.01(0.01,0.02)	0.03(-0.16,0.23)	0.42(0.32,0.52)	0.75(0.57,0.98)	0.70(0.53,0.89)	0.62(0.48,0.82)	-0.19(-0.35,-0.04)
Guam	0.00(0.00,0.00)	0.00(0.00,0.00)	0.00(0.00,0.00)	0.00(0.00,0.00)	0.07(-0.50,0.64)	0.06(0.04,0.09)	0.09(0.06,0.14)	0.05(0.03,0.08)	0.05(0.03,0.08)	0.08(0.02,0.15)
Guatemala	0.12(0.10,0.16)	0.49(0.35,0.67)	0.00(0.00,0.00)	0.00(0.00,0.01)	1.00(0.42,1.58)	7.05(5.41,9.19)	25.29(17.89,34.01)	0.15(0.12,0.20)	0.20(0.14,0.27)	0.88(0.40,1.35)
Guinea	0.00(0.00,0.00)	0.00(0.00,0.00)	0.00(0.00,0.00)	0.00(0.00,0.00)	0.98(0.90,1.05)	2.14(1.35,3.15)	4.21(2.62,6.22)	0.05(0.03,0.08)	0.05(0.03,0.08)	-0.15(-0.22,-0.08)
Guinea-Bissau	0.00(0.00,0.00)	0.00(0.00,0.00)	0.00(0.00,0.00)	0.00(0.00,0.00)	1.47(1.15,1.79)	0.19(0.12,0.28)	0.57(0.36,0.90)	0.03(0.02,0.05)	0.04(0.03,0.07)	1.37(1.10,1.63)
Guyana	0.02(0.02,0.03)	0.04(0.03,0.06)	0.00(0.00,0.01)	0.01(0.00,0.01)	1.82(1.58,2.05)	1.23(0.94,1.55)	2.31(1.58,3.29)	0.24(0.19,0.30)	0.31(0.21,0.44)	1.38(1.21,1.55)
Haiti	0.13(0.05,0.24)	0.28(0.11,0.55)	0.00(0.00,0.01)	0.00(0.00,0.01)	-0.38(-0.49,-0.27)	8.91(5.05,13.92)	17.92(10.20,30.41)	0.21(0.12,0.32)	0.17(0.09,0.28)	-0.79(-0.93,-0.65)
Honduras	0.07(0.04,0.11)	0.27(0.14,0.44)	0.00(0.00,0.00)	0.00(0.00,0.01)	0.85(0.77,0.93)	4.96(3.37,6.99)	17.85(11.63,26.98)	0.19(0.13,0.27)	0.23(0.15,0.34)	0.56(0.50,0.63)
Hungary	26.21(21.73,30.69)	10.92(8.52,13.81)	0.20(0.17,0.24)	0.08(0.06,0.09)	-3.53(-3.70,-3.35)	1350.24(1125.19,1619.91)	554.20(436.39,699.92)	10.68(8.91,12.82)	4.13(3.22,5.22)	-3.41(-3.52,-3.30)
Iceland	0.43(0.36,0.53)	0.61(0.47,0.75)	0.17(0.14,0.20)	0.12(0.10,0.15)	-0.83(-1.01,-0.65)	29.30(23.59,35.73)	37.18(28.32,47.48)	11.46(9.22,13.94)	8.36(6.35,10.70)	-1.01(-1.16,-0.86)
India	4.15(1.34,7.23)	12.08(6.40,17.79)	0.00(0.00,0.00)	0.00(0.00,0.00)	0.67(0.56,0.78)	1067.44(739.43,1510.67)	1930.78(1370.45,2634.44)	0.17(0.12,0.24)	0.14(0.10,0.19)	-0.65(-0.66,-0.63)
Indonesia	0.12(0.07,0.18)	0.65(0.46,0.86)	0.00(0.00,0.00)	0.00(0.00,0.00)	2.50(2.15,2.84)	61.62(41.23,91.51)	175.74(118.50,249.34)	0.04(0.03,0.06)	0.06(0.04,0.08)	0.90(0.86,0.95)
Iran (Islamic Republic of)	7.94(3.64,12.31)	31.30(23.09,40.93)	0.02(0.01,0.04)	0.03(0.02,0.04)	1.31(1.13,1.49)	696.51(470.63,946.26)	2260.75(1736.63,2918.72)	2.03(1.36,2.75)	2.24(1.71,2.89)	0.97(0.78,1.16)
Iraq	0.68(0.25,1.24)	2.46(1.44,3.93)	0.01(0.00,0.01)	0.01(0.00,0.01)	0.07(-0.02,0.16)	134.08(90.76,185.94)	452.88(310.14,626.75)	1.32(0.90,1.83)	1.33(0.91,1.82)	0.03(-0.02,0.07)
Ireland	7.81(6.52,9.15)	6.83(5.33,8.51)	0.21(0.17,0.24)	0.10(0.08,0.12)	-2.28(-2.40,-2.17)	445.18(362.56,537.33)	439.13(336.31,555.34)	12.26(9.97,14.80)	6.75(5.15,8.56)	-1.88(-1.96,-1.79)
Israel	1.82(1.50,2.20)	3.09(2.50,3.84)	0.04(0.03,0.05)	0.03(0.02,0.04)	-0.84(-0.98,-0.69)	121.92(94.44,153.35)	199.45(154.44,253.06)	2.70(2.09,3.39)	1.97(1.52,2.51)	-0.93(-1.03,-0.84)
Italy	52.25(43.72,61.56)	58.82(47.81,71.06)	0.07(0.06,0.08)	0.06(0.05,0.07)	-0.24(-0.48,0.00)	3785.52(3013.90,4657.05)	3849.24(2979.53,4867.58)	5.38(4.29,6.61)	4.26(3.26,5.45)	-0.48(-0.59,-0.36)
Jamaica	0.13(0.10,0.15)	0.33(0.22,0.49)	0.01(0.01,0.01)	0.01(0.01,0.02)	1.14(0.67,1.60)	7.65(6.01,9.60)	16.72(11.71,23.03)	0.46(0.36,0.58)	0.55(0.38,0.75)	0.46(0.12,0.79)
Japan	8.22(6.98,9.37)	7.63(6.20,9.10)	0.00(0.00,0.01)	0.00(0.00,0.00)	-1.17(-1.47,-0.87)	917.16(681.41,1214.53)	706.45(520.51,949.52)	0.57(0.42,0.75)	0.39(0.28,0.52)	-1.29(-1.45,-1.13)
Jordan	0.66(0.42,0.98)	2.86(1.94,4.14)	0.04(0.02,0.05)	0.03(0.02,0.04)	-1.08(-1.40,-0.77)	62.88(46.22,83.51)	295.46(213.50,396.21)	3.17(2.35,4.17)	2.64(1.91,3.52)	-0.75(-0.91,-0.59)
Kazakhstan	5.08(3.95,6.57)	3.64(2.09,6.60)	0.04(0.03,0.05)	0.02(0.01,0.03)	-2.46(-2.73,-2.19)	414.90(323.64,538.97)	465.80(325.42,681.45)	2.89(2.26,3.76)	2.26(1.58,3.30)	-0.81(-0.98,-0.63)
Kenya	0.04(0.01,0.07)	0.15(0.07,0.24)	0.00(0.00,0.00)	0.00(0.00,0.00)	0.19(0.02,0.35)	7.38(4.91,10.51)	18.00(12.41,24.99)	0.06(0.04,0.09)	0.05(0.04,0.07)	-1.02(-1.20,-0.84)
Kiribati	0.00(0.00,0.00)	0.00(0.00,0.00)	0.00(0.00,0.00)	0.00(0.00,0.00)	-0.78(-0.99,-0.57)	0.03(0.02,0.05)	0.07(0.05,0.11)	0.06(0.04,0.09)	0.07(0.04,0.11)	0.51(0.23,0.80)
Kuwait	0.00(0.00,0.00)	0.30(0.22,0.39)	0.00(0.00,0.00)	0.01(0.00,0.01)	16.02(10.84,21.44)	15.47(9.55,23.41)	103.21(67.16,142.85)	1.12(0.70,1.66)	1.68(1.13,2.32)	1.44(1.20,1.68)
Kyrgyzstan	0.41(0.32,0.51)	0.66(0.49,0.90)	0.01(0.01,0.02)	0.01(0.01,0.02)	2.16(0.99,3.35)	31.00(23.50,39.75)	66.37(48.38,88.05)	0.97(0.74,1.24)	1.12(0.83,1.49)	1.62(1.10,2.14)
Lao People's Democratic Republic	0.00(0.00,0.01)	0.02(0.01,0.03)	0.00(0.00,0.00)	0.00(0.00,0.00)	2.18(1.93,2.43)	1.49(0.98,2.26)	3.92(2.56,5.70)	0.05(0.04,0.08)	0.06(0.04,0.09)	0.32(0.29,0.35)
Latvia	5.43(4.51,6.54)	1.76(1.33,2.23)	0.17(0.14,0.21)	0.07(0.05,0.09)	-4.13(-4.51,-3.74)	244.34(203.74,290.45)	80.50(61.76,100.14)	8.01(6.68,9.49)	3.35(2.57,4.15)	-3.88(-4.24,-3.52)
Lebanon	0.47(0.23,0.79)	1.57(0.97,2.30)	0.02(0.01,0.03)	0.03(0.02,0.04)	1.24(0.97,1.51)	56.18(39.03,76.36)	219.76(157.29,310.08)	2.26(1.57,3.05)	3.66(2.61,5.13)	1.58(1.31,1.84)
Lesotho	0.01(0.00,0.01)	0.02(0.01,0.03)	0.00(0.00,0.00)	0.00(0.00,0.00)	2.98(2.69,3.27)	1.30(0.88,1.87)	2.84(1.90,4.01)	0.13(0.09,0.19)	0.20(0.13,0.28)	1.63(1.56,1.71)
Liberia	0.00(0.00,0.00)	0.00(0.00,0.00)	0.00(0.00,0.00)	0.00(0.00,0.00)	-0.23(-0.33,-0.12)	0.59(0.37,0.92)	1.35(0.84,2.05)	0.04(0.03,0.06)	0.04(0.02,0.06)	-0.62(-0.72,-0.52)
Libya	0.25(0.12,0.42)	2.50(1.36,4.24)	0.01(0.01,0.02)	0.03(0.02,0.06)	3.81(3.63,4.00)	31.43(21.95,44.58)	184.19(126.90,276.38)	1.24(0.87,1.73)	2.28(1.60,3.31)	2.23(2.16,2.30)
Lithuania	6.17(5.04,7.47)	2.73(2.10,3.39)	0.15(0.12,0.18)	0.07(0.06,0.09)	-3.16(-3.51,-2.82)	279.40(228.17,335.65)	121.07(94.45,149.18)	6.98(5.68,8.36)	3.47(2.73,4.29)	-2.97(-3.29,-2.66)
Luxembourg	0.70(0.50,0.91)	0.87(0.62,1.17)	0.14(0.10,0.18)	0.09(0.06,0.12)	-1.10(-1.26,-0.95)	40.65(28.96,53.80)	55.07(38.22,75.11)	8.51(6.07,11.27)	6.07(4.22,8.29)	-1.00(-1.08,-0.92)
Madagascar	0.03(0.00,0.05)	0.05(0.01,0.10)	0.00(0.00,0.00)	0.00(0.00,0.00)	-1.71(-2.11,-1.31)	5.20(3.35,7.74)	7.52(4.47,11.11)	0.08(0.05,0.11)	0.04(0.02,0.06)	-2.42(-2.80,-2.04)
Malawi	0.02(0.00,0.04)	0.08(0.02,0.17)	0.00(0.00,0.00)	0.00(0.00,0.00)	2.05(1.87,2.23)	3.27(2.00,4.95)	9.28(5.25,14.86)	0.06(0.04,0.10)	0.08(0.05,0.13)	0.93(0.88,0.98)
Malaysia	0.02(0.01,0.04)	0.13(0.08,0.20)	0.00(0.00,0.00)	0.00(0.00,0.00)	3.58(2.80,4.36)	6.67(4.36,9.85)	16.98(11.98,23.26)	0.05(0.03,0.07)	0.05(0.04,0.07)	0.34(0.09,0.60)
Maldives	0.00(0.00,0.00)	0.00(0.00,0.00)	0.00(0.00,0.00)	0.00(0.00,0.00)	0.18(-0.23,0.59)	0.08(0.05,0.12)	0.35(0.22,0.53)	0.06(0.04,0.09)	0.05(0.04,0.08)	-0.85(-0.96,-0.75)
Mali	0.00(0.00,0.00)	0.00(0.00,0.00)	0.00(0.00,0.00)	0.00(0.00,0.00)	2.62(2.29,2.95)	1.92(1.20,2.89)	7.20(4.50,10.50)	0.04(0.02,0.05)	0.06(0.04,0.08)	1.87(1.71,2.02)
Malta	0.23(0.19,0.27)	0.32(0.23,0.41)	0.05(0.05,0.06)	0.05(0.03,0.06)	-0.30(-0.39,-0.22)	12.61(10.23,15.33)	16.44(12.55,21.40)	2.95(2.39,3.58)	2.72(2.08,3.55)	-0.15(-0.25,-0.05)
Marshall Islands	0.00(0.00,0.00)	0.00(0.00,0.00)	0.00(0.00,0.00)	0.00(0.00,0.00)	0.27(0.21,0.33)	0.01(0.01,0.02)	0.02(0.01,0.03)	0.04(0.02,0.06)	0.04(0.02,0.06)	-0.00(-0.06,0.06)
Mauritania	0.00(0.00,0.00)	0.00(0.00,0.00)	0.00(0.00,0.00)	0.00(0.00,0.00)	-0.35(-0.61,-0.09)	1.51(0.96,2.29)	2.78(1.74,4.03)	0.11(0.07,0.17)	0.09(0.06,0.13)	-0.81(-0.87,-0.75)
Mauritius	0.00(0.00,0.00)	0.10(0.08,0.12)	0.00(0.00,0.00)	0.01(0.00,0.01)	24.46(21.44,27.56)	0.52(0.33,0.77)	4.33(3.49,5.37)	0.05(0.03,0.08)	0.26(0.21,0.32)	7.01(5.97,8.07)
Mexico	4.69(3.91,5.52)	13.74(10.62,17.30)	0.01(0.01,0.01)	0.01(0.01,0.01)	-0.07(-0.57,0.44)	264.04(215.40,320.79)	641.54(501.54,793.91)	0.48(0.40,0.58)	0.46(0.36,0.57)	-0.43(-0.77,-0.08)
Micronesia (Federated States of)	0.00(0.00,0.00)	0.00(0.00,0.00)	0.00(0.00,0.00)	0.00(0.00,0.00)	0.04(-0.04,0.11)	0.03(0.02,0.05)	0.05(0.03,0.08)	0.05(0.03,0.08)	0.05(0.03,0.08)	0.11(0.07,0.15)
Monaco	0.01(0.01,0.02)	0.02(0.01,0.04)	0.03(0.02,0.05)	0.04(0.02,0.07)	0.93(0.61,1.25)	1.33(0.90,2.03)	1.87(1.16,2.84)	3.16(2.13,4.79)	3.48(2.15,5.30)	0.32(0.22,0.42)
Mongolia	0.17(0.05,0.36)	0.53(0.25,0.97)	0.02(0.00,0.03)	0.02(0.01,0.04)	1.41(1.21,1.62)	11.20(6.28,19.37)	36.41(22.81,54.82)	0.93(0.53,1.57)	1.17(0.75,1.74)	1.18(1.02,1.33)
Montenegro	1.10(0.67,1.62)	1.24(0.80,1.74)	0.17(0.10,0.25)	0.14(0.09,0.20)	-0.44(-0.66,-0.21)	59.61(40.96,82.96)	67.59(48.40,89.42)	9.05(6.21,12.80)	8.21(5.91,11.00)	-0.22(-0.44,0.00)
Morocco	0.91(0.25,1.75)	3.88(1.63,6.96)	0.01(0.00,0.01)	0.01(0.00,0.02)	1.64(1.43,1.84)	139.09(93.46,205.00)	378.77(257.03,549.08)	0.78(0.52,1.15)	0.96(0.65,1.38)	0.56(0.50,0.61)
Mozambique	0.03(0.00,0.05)	0.10(0.02,0.20)	0.00(0.00,0.00)	0.00(0.00,0.00)	2.24(2.14,2.34)	4.82(3.06,7.22)	11.45(6.45,18.01)	0.06(0.04,0.09)	0.07(0.04,0.11)	0.63(0.51,0.75)
Myanmar	0.07(0.02,0.14)	0.13(0.06,0.22)	0.00(0.00,0.00)	0.00(0.00,0.00)	-0.98(-1.22,-0.73)	20.05(12.90,29.54)	21.71(13.91,32.13)	0.07(0.04,0.10)	0.04(0.02,0.06)	-2.08(-2.27,-1.88)
Namibia	0.01(0.00,0.01)	0.01(0.01,0.02)	0.00(0.00,0.00)	0.00(0.00,0.00)	-0.10(-0.31,0.11)	0.89(0.59,1.27)	1.58(1.09,2.25)	0.11(0.07,0.16)	0.09(0.06,0.13)	-1.01(-1.14,-0.87)
Nauru	0.00(0.00,0.00)	0.00(0.00,0.00)	0.00(0.00,0.00)	0.00(0.00,0.00)	-0.24(-0.38,-0.11)	0.00(0.00,0.01)	0.00(0.00,0.01)	0.04(0.03,0.07)	0.05(0.03,0.07)	-0.04(-0.10,0.02)
Nepal	0.10(0.02,0.19)	0.25(0.06,0.48)	0.00(0.00,0.00)	0.00(0.00,0.00)	0.35(0.19,0.51)	29.76(20.78,43.13)	46.16(30.68,65.85)	0.24(0.17,0.34)	0.18(0.12,0.25)	-1.29(-1.39,-1.19)
Netherlands	28.65(23.95,33.88)	32.26(26.42,38.97)	0.16(0.13,0.19)	0.11(0.09,0.13)	-0.77(-0.88,-0.65)	1629.76(1330.12,1958.07)	1655.26(1322.11,2050.60)	9.23(7.52,11.12)	6.70(5.32,8.36)	-0.79(-0.87,-0.72)
New Zealand	2.70(2.19,3.30)	3.07(2.41,3.88)	0.07(0.06,0.09)	0.04(0.03,0.05)	-2.29(-2.62,-1.96)	135.17(109.41,167.68)	141.65(110.24,177.88)	3.72(3.02,4.64)	2.10(1.64,2.64)	-2.22(-2.47,-1.98)
Nicaragua	0.05(0.03,0.07)	0.25(0.16,0.39)	0.00(0.00,0.00)	0.00(0.00,0.01)	2.51(2.06,2.97)	3.76(2.71,5.09)	16.11(10.81,23.22)	0.18(0.13,0.24)	0.27(0.18,0.39)	1.89(1.62,2.17)
Niger	0.00(0.00,0.00)	0.00(0.00,0.00)	0.00(0.00,0.00)	0.00(0.00,0.00)	0.22(-0.00,0.45)	1.37(0.87,2.02)	3.72(2.32,5.71)	0.03(0.02,0.04)	0.03(0.02,0.04)	-0.15(-0.24,-0.06)
Nigeria	0.00(0.00,0.00)	0.00(0.00,0.00)	0.00(0.00,0.00)	0.00(0.00,0.00)	1.05(0.98,1.11)	16.05(10.18,24.19)	44.35(27.29,66.55)	0.03(0.02,0.04)	0.03(0.02,0.05)	0.37(0.19,0.56)
Niue	0.00(0.00,0.00)	0.00(0.00,0.00)	0.00(0.00,0.00)	0.00(0.00,0.00)	-0.19(-0.28,-0.10)	0.00(0.00,0.00)	0.00(0.00,0.00)	0.06(0.03,0.09)	0.05(0.03,0.09)	-0.16(-0.22,-0.11)
North Macedonia	2.63(1.74,3.65)	2.82(1.88,4.20)	0.13(0.08,0.18)	0.09(0.06,0.13)	-1.42(-1.62,-1.21)	136.63(99.75,178.79)	168.20(124.91,221.18)	6.53(4.77,8.56)	5.47(4.06,7.21)	-0.72(-0.86,-0.57)
Northern Mariana Islands	0.00(0.00,0.00)	0.00(0.00,0.00)	0.00(0.00,0.00)	0.00(0.00,0.00)	-0.21(-0.32,-0.09)	0.02(0.01,0.04)	0.03(0.02,0.05)	0.06(0.03,0.08)	0.05(0.03,0.09)	-0.17(-0.25,-0.09)
Norway	10.52(8.97,12.21)	9.21(7.45,11.14)	0.20(0.17,0.23)	0.11(0.09,0.14)	-2.06(-2.51,-1.61)	526.24(437.55,626.92)	576.41(450.73,721.56)	10.70(8.90,12.81)	7.96(6.21,9.96)	-1.24(-1.52,-0.96)
Oman	0.05(0.02,0.10)	0.37(0.17,0.67)	0.00(0.00,0.01)	0.01(0.00,0.02)	2.53(1.93,3.13)	9.14(6.11,13.21)	48.67(32.01,69.58)	0.69(0.47,0.98)	0.99(0.66,1.41)	1.35(1.14,1.57)
Pakistan	0.65(0.13,1.25)	1.65(0.65,2.92)	0.00(0.00,0.00)	0.00(0.00,0.00)	-0.20(-0.48,0.08)	173.30(117.63,248.50)	331.06(226.00,471.17)	0.25(0.17,0.36)	0.19(0.13,0.27)	-1.04(-1.17,-0.90)
Palau	0.00(0.00,0.00)	0.00(0.00,0.00)	0.00(0.00,0.00)	0.00(0.00,0.00)	-0.16(-0.25,-0.07)	0.00(0.00,0.01)	0.01(0.01,0.01)	0.03(0.02,0.05)	0.03(0.02,0.05)	-0.10(-0.20,0.01)
Palestine	0.22(0.09,0.40)	1.25(0.76,1.90)	0.02(0.01,0.04)	0.04(0.02,0.06)	2.92(2.29,3.55)	17.97(11.74,27.63)	94.97(65.40,132.27)	1.72(1.11,2.64)	2.59(1.80,3.58)	1.93(1.65,2.22)
Panama	0.05(0.04,0.06)	0.22(0.15,0.31)	0.00(0.00,0.00)	0.01(0.00,0.01)	1.31(0.89,1.73)	3.20(2.47,4.12)	10.63(7.50,14.40)	0.18(0.14,0.24)	0.24(0.17,0.33)	0.71(0.46,0.96)
Papua New Guinea	0.00(0.00,0.00)	0.00(0.00,0.00)	0.00(0.00,0.00)	0.00(0.00,0.00)	0.23(0.11,0.36)	0.85(0.48,1.32)	2.86(1.61,4.58)	0.03(0.02,0.05)	0.03(0.02,0.05)	0.24(0.14,0.35)
Paraguay	0.12(0.08,0.16)	0.52(0.33,0.77)	0.01(0.00,0.01)	0.01(0.01,0.01)	0.98(0.49,1.47)	13.76(9.94,19.29)	34.52(22.89,51.52)	0.54(0.39,0.75)	0.54(0.36,0.80)	-0.23(-0.35,-0.10)
Peru	0.20(0.13,0.28)	1.10(0.60,1.75)	0.00(0.00,0.00)	0.00(0.00,0.01)	2.92(2.59,3.25)	13.27(9.52,18.54)	61.13(40.47,89.10)	0.09(0.07,0.13)	0.17(0.11,0.25)	2.07(1.94,2.21)
Philippines	0.92(0.58,1.25)	2.02(1.52,2.63)	0.00(0.00,0.00)	0.00(0.00,0.00)	-0.76(-1.00,-0.52)	75.64(55.77,101.51)	142.53(109.47,185.26)	0.17(0.13,0.23)	0.14(0.10,0.18)	-1.03(-1.18,-0.87)
Poland	127.54(108.10,145.27)	51.89(41.76,63.36)	0.30(0.25,0.34)	0.09(0.07,0.11)	-4.09(-4.27,-3.92)	6296.31(5311.69,7228.21)	2649.55(2138.14,3232.64)	14.79(12.50,16.90)	4.89(3.94,5.97)	-3.75(-3.88,-3.61)
Portugal	5.44(4.63,6.33)	5.36(4.36,6.41)	0.04(0.04,0.05)	0.03(0.03,0.04)	-0.91(-1.02,-0.80)	315.16(252.83,383.98)	303.36(247.27,376.99)	2.70(2.16,3.30)	2.02(1.63,2.51)	-0.90(-0.97,-0.82)
Puerto Rico	0.80(0.56,1.10)	1.08(0.72,1.55)	0.02(0.02,0.03)	0.02(0.01,0.03)	0.12(-0.09,0.32)	37.85(26.67,52.04)	48.99(33.31,69.40)	1.06(0.75,1.46)	1.12(0.76,1.58)	0.26(0.09,0.42)
Qatar	0.00(0.00,0.01)	0.03(0.02,0.06)	0.00(0.00,0.00)	0.00(0.00,0.00)	-1.21(-1.48,-0.95)	3.82(2.26,5.98)	52.85(32.00,79.63)	0.91(0.55,1.40)	1.34(0.85,2.00)	0.75(0.52,0.98)
Republic of Korea	2.21(1.67,2.81)	1.69(1.17,2.32)	0.01(0.00,0.01)	0.00(0.00,0.00)	-3.54(-3.68,-3.39)	237.55(177.61,317.66)	234.13(163.19,320.20)	0.58(0.43,0.77)	0.30(0.21,0.42)	-2.28(-2.36,-2.20)
Republic of Moldova	1.47(1.12,1.89)	1.09(0.80,1.41)	0.03(0.02,0.04)	0.02(0.02,0.03)	-1.91(-2.26,-1.56)	70.50(53.15,90.67)	60.04(45.26,77.24)	1.53(1.15,1.97)	1.20(0.91,1.55)	-1.28(-1.56,-1.00)
Romania	26.16(21.70,31.43)	10.34(8.03,12.99)	0.10(0.08,0.12)	0.04(0.03,0.05)	-3.86(-4.14,-3.58)	1236.86(1012.36,1482.57)	521.12(410.49,640.56)	4.74(3.88,5.66)	2.01(1.58,2.46)	-3.40(-3.64,-3.16)
Russian Federation	105.64(89.79,125.37)	93.97(76.89,112.16)	0.06(0.05,0.07)	0.05(0.04,0.06)	-2.00(-2.56,-1.44)	5730.59(4863.44,6826.75)	5513.09(4551.13,6630.63)	3.37(2.85,4.02)	2.86(2.36,3.43)	-1.36(-1.85,-0.87)
Rwanda	0.03(0.01,0.05)	0.09(0.03,0.16)	0.00(0.00,0.00)	0.00(0.00,0.00)	0.81(0.67,0.95)	2.98(1.85,4.37)	8.19(4.67,12.69)	0.08(0.05,0.12)	0.10(0.05,0.15)	0.37(0.30,0.44)
Saint Kitts and Nevis	0.01(0.01,0.01)	0.01(0.01,0.02)	0.02(0.02,0.03)	0.02(0.01,0.02)	-0.87(-1.13,-0.62)	0.30(0.23,0.37)	0.57(0.42,0.77)	1.00(0.78,1.26)	0.71(0.51,0.96)	-1.04(-1.20,-0.88)
Saint Lucia	0.01(0.01,0.01)	0.02(0.02,0.03)	0.01(0.01,0.02)	0.01(0.01,0.01)	-0.79(-0.91,-0.67)	0.54(0.44,0.69)	1.13(0.83,1.50)	0.60(0.48,0.76)	0.48(0.35,0.64)	-0.80(-0.92,-0.67)
Saint Vincent and the Grenadines	0.00(0.00,0.00)	0.00(0.00,0.00)	0.00(0.00,0.00)	0.00(0.00,0.00)	0.61(0.38,0.85)	0.10(0.08,0.14)	0.24(0.17,0.34)	0.15(0.11,0.19)	0.18(0.13,0.25)	0.42(0.32,0.52)
Samoa	0.00(0.00,0.00)	0.00(0.00,0.00)	0.00(0.00,0.00)	0.00(0.00,0.00)	0.12(0.02,0.22)	0.07(0.05,0.11)	0.12(0.08,0.18)	0.07(0.04,0.10)	0.07(0.04,0.11)	-0.05(-0.15,0.05)
San Marino	0.00(0.00,0.00)	0.00(0.00,0.00)	0.00(0.00,0.00)	0.00(0.00,0.00)	-0.30(-0.65,0.05)	0.48(0.29,0.76)	0.69(0.40,1.07)	1.74(1.06,2.79)	1.52(0.91,2.35)	-0.55(-0.66,-0.44)
Sao Tome and Principe	0.00(0.00,0.00)	0.00(0.00,0.00)	0.00(0.00,0.00)	0.00(0.00,0.00)	0.73(0.52,0.94)	0.02(0.01,0.03)	0.06(0.03,0.08)	0.03(0.02,0.05)	0.03(0.02,0.05)	0.11(-0.09,0.31)
Saudi Arabia	0.10(0.04,0.19)	2.01(1.09,3.61)	0.00(0.00,0.00)	0.00(0.00,0.01)	6.69(5.52,7.86)	52.19(33.32,77.71)	362.37(233.09,552.90)	0.50(0.32,0.74)	0.79(0.52,1.19)	1.91(1.71,2.11)
Senegal	0.00(0.00,0.00)	0.00(0.00,0.00)	0.00(0.00,0.00)	0.00(0.00,0.00)	0.07(-0.24,0.39)	3.71(2.36,5.59)	7.35(4.62,11.04)	0.09(0.06,0.13)	0.07(0.04,0.10)	-0.86(-0.96,-0.75)
Serbia	16.63(10.31,26.04)	15.46(10.54,22.55)	0.14(0.09,0.23)	0.12(0.08,0.18)	-0.72(-1.12,-0.32)	823.01(566.15,1225.14)	789.07(582.56,1063.90)	7.25(5.06,10.71)	6.69(4.91,9.03)	-0.42(-0.81,-0.04)
Seychelles	0.00(0.00,0.00)	0.00(0.00,0.00)	0.00(0.00,0.00)	0.00(0.00,0.00)	3.32(2.29,4.36)	0.03(0.02,0.05)	0.08(0.05,0.11)	0.06(0.03,0.08)	0.06(0.04,0.09)	0.12(0.06,0.19)
Sierra Leone	0.00(0.00,0.00)	0.00(0.00,0.00)	0.00(0.00,0.00)	0.00(0.00,0.00)	-0.19(-0.30,-0.09)	1.67(1.08,2.43)	2.80(1.79,4.13)	0.06(0.04,0.09)	0.05(0.03,0.07)	-0.91(-0.97,-0.85)
Singapore	0.04(0.03,0.04)	0.04(0.03,0.05)	0.00(0.00,0.00)	0.00(0.00,0.00)	-3.31(-3.66,-2.96)	4.76(3.25,6.80)	7.60(5.26,10.85)	0.16(0.11,0.22)	0.09(0.06,0.13)	-1.78(-1.86,-1.70)
Slovakia	4.86(3.36,7.10)	4.39(2.93,6.48)	0.09(0.06,0.12)	0.05(0.04,0.08)	-1.53(-1.69,-1.36)	241.94(172.63,334.41)	222.70(161.27,307.33)	4.27(3.06,5.91)	2.94(2.12,4.08)	-1.27(-1.44,-1.11)
Slovenia	5.36(4.43,6.51)	2.82(2.13,3.65)	0.23(0.19,0.27)	0.08(0.06,0.11)	-3.03(-3.16,-2.89)	249.31(206.05,303.44)	151.33(119.03,191.88)	10.66(8.81,12.90)	4.98(3.90,6.29)	-2.25(-2.37,-2.13)
Solomon Islands	0.00(0.00,0.00)	0.00(0.00,0.00)	0.00(0.00,0.00)	0.00(0.00,0.00)	0.86(0.60,1.13)	0.11(0.07,0.17)	0.34(0.21,0.51)	0.05(0.03,0.08)	0.06(0.04,0.09)	0.69(0.54,0.85)
Somalia	0.01(0.00,0.02)	0.02(0.00,0.06)	0.00(0.00,0.00)	0.00(0.00,0.00)	-0.20(-0.28,-0.12)	2.08(1.19,3.48)	4.50(2.44,7.65)	0.05(0.03,0.08)	0.04(0.02,0.07)	-0.60(-0.76,-0.44)
South Africa	2.86(1.92,3.77)	3.58(2.61,4.69)	0.01(0.01,0.02)	0.01(0.00,0.01)	-2.15(-2.26,-2.04)	166.07(125.29,214.81)	192.54(146.90,247.41)	0.64(0.48,0.83)	0.34(0.26,0.44)	-2.25(-2.36,-2.14)
South Sudan	0.01(0.00,0.02)	0.02(0.00,0.05)	0.00(0.00,0.00)	0.00(0.00,0.00)	0.81(0.68,0.94)	1.64(0.99,2.60)	2.83(1.55,4.66)	0.05(0.03,0.08)	0.05(0.03,0.08)	-0.37(-0.51,-0.22)
Spain	27.35(23.19,31.74)	30.07(23.76,36.78)	0.06(0.05,0.07)	0.04(0.03,0.05)	-1.16(-1.29,-1.03)	2007.61(1575.95,2481.36)	2549.13(1911.44,3297.21)	4.67(3.63,5.77)	3.84(2.85,5.00)	-0.85(-1.02,-0.68)
Sri Lanka	0.07(0.04,0.11)	0.08(0.04,0.13)	0.00(0.00,0.00)	0.00(0.00,0.00)	-2.51(-2.84,-2.18)	7.90(5.42,10.99)	8.15(5.72,11.24)	0.05(0.04,0.08)	0.03(0.02,0.04)	-2.15(-2.40,-1.90)
Sudan	0.53(0.19,1.04)	2.72(1.45,4.69)	0.00(0.00,0.01)	0.01(0.01,0.02)	2.31(1.95,2.67)	77.27(48.70,114.38)	274.32(182.33,400.77)	0.64(0.40,0.95)	0.93(0.61,1.34)	1.11(0.96,1.27)
Suriname	0.04(0.02,0.05)	0.08(0.04,0.13)	0.01(0.01,0.02)	0.01(0.01,0.02)	0.02(-0.26,0.30)	1.89(1.37,2.49)	4.00(2.53,6.06)	0.63(0.45,0.83)	0.60(0.38,0.92)	-0.16(-0.42,0.09)
Sweden	13.74(11.38,16.15)	20.16(15.47,25.38)	0.12(0.10,0.14)	0.12(0.09,0.14)	0.46(0.22,0.69)	994.35(791.38,1246.39)	1371.34(1007.97,1804.83)	9.21(7.31,11.62)	9.49(7.04,12.56)	0.14(0.03,0.25)
Switzerland	17.56(14.34,21.18)	15.59(12.24,19.30)	0.20(0.16,0.24)	0.11(0.08,0.13)	-1.68(-1.83,-1.54)	948.18(765.70,1153.71)	854.56(655.22,1076.83)	11.11(8.96,13.52)	6.61(5.04,8.36)	-1.62(-1.69,-1.54)
Syrian Arab Republic	0.33(0.13,0.63)	0.81(0.41,1.38)	0.01(0.00,0.01)	0.01(0.00,0.01)	-0.21(-0.30,-0.12)	75.16(52.29,105.20)	163.91(111.30,231.96)	1.09(0.75,1.54)	1.05(0.73,1.48)	-0.23(-0.32,-0.15)
Taiwan (Province of China)	0.14(0.12,0.17)	0.14(0.10,0.17)	0.00(0.00,0.00)	0.00(0.00,0.00)	-7.29(-8.89,-5.65)	21.46(15.38,29.23)	45.41(31.37,61.64)	0.11(0.08,0.15)	0.14(0.09,0.19)	-1.29(-1.99,-0.58)
Tajikistan	0.20(0.11,0.29)	0.21(0.07,0.44)	0.01(0.00,0.01)	0.00(0.00,0.01)	-2.10(-2.76,-1.45)	24.01(16.69,33.73)	33.86(20.96,51.21)	0.78(0.56,1.10)	0.43(0.27,0.64)	-1.91(-2.07,-1.75)
Thailand	0.12(0.05,0.21)	0.37(0.19,0.60)	0.00(0.00,0.00)	0.00(0.00,0.00)	1.07(0.87,1.26)	32.36(22.09,45.06)	55.07(38.65,79.25)	0.07(0.05,0.09)	0.06(0.04,0.09)	-0.38(-0.50,-0.27)
Timor-Leste	0.00(0.00,0.00)	0.00(0.00,0.00)	0.00(0.00,0.00)	0.00(0.00,0.00)	1.91(1.69,2.13)	0.21(0.13,0.32)	0.42(0.27,0.66)	0.04(0.03,0.06)	0.04(0.03,0.07)	0.23(0.16,0.29)
Togo	0.00(0.00,0.00)	0.00(0.00,0.00)	0.00(0.00,0.00)	0.00(0.00,0.00)	-0.16(-0.30,-0.02)	1.86(1.16,2.69)	5.00(2.90,7.69)	0.10(0.07,0.15)	0.09(0.05,0.13)	-0.46(-0.56,-0.36)
Tokelau	0.00(0.00,0.00)	0.00(0.00,0.00)	0.00(0.00,0.00)	0.00(0.00,0.00)	-0.17(-0.26,-0.09)	0.00(0.00,0.00)	0.00(0.00,0.00)	0.04(0.02,0.07)	0.04(0.03,0.07)	-0.04(-0.11,0.03)
Tonga	0.00(0.00,0.00)	0.00(0.00,0.00)	0.00(0.00,0.00)	0.00(0.00,0.00)	0.16(0.07,0.26)	0.04(0.02,0.06)	0.05(0.03,0.08)	0.06(0.04,0.09)	0.06(0.04,0.09)	-0.21(-0.27,-0.16)
Trinidad and Tobago	0.08(0.07,0.10)	0.18(0.12,0.26)	0.01(0.01,0.01)	0.01(0.01,0.01)	0.04(-0.10,0.17)	4.60(3.64,5.71)	8.99(6.45,12.56)	0.48(0.39,0.60)	0.50(0.36,0.70)	0.04(-0.07,0.14)
Tunisia	0.63(0.20,1.15)	3.21(1.61,5.62)	0.01(0.00,0.02)	0.02(0.01,0.04)	1.94(1.53,2.34)	86.32(58.24,121.10)	292.94(201.67,411.78)	1.46(0.98,2.04)	2.07(1.43,2.89)	1.10(0.98,1.22)
Türkiye	12.14(5.95,19.16)	25.70(17.48,36.36)	0.03(0.01,0.05)	0.03(0.02,0.04)	-0.51(-0.62,-0.40)	1401.59(988.05,1865.85)	2438.67(1807.25,3232.94)	3.17(2.23,4.21)	2.47(1.83,3.28)	-0.96(-1.01,-0.90)
Turkmenistan	0.72(0.56,0.91)	1.02(0.53,1.59)	0.04(0.03,0.05)	0.02(0.01,0.04)	-1.17(-1.46,-0.89)	48.37(37.17,60.62)	80.62(52.87,112.21)	2.17(1.68,2.73)	1.66(1.08,2.31)	-0.81(-0.95,-0.68)
Tuvalu	0.00(0.00,0.00)	0.00(0.00,0.00)	0.00(0.00,0.00)	0.00(0.00,0.00)	0.30(0.17,0.43)	0.00(0.00,0.01)	0.01(0.00,0.01)	0.04(0.02,0.07)	0.04(0.03,0.07)	0.16(0.11,0.22)
Uganda	0.01(0.00,0.03)	0.07(0.02,0.14)	0.00(0.00,0.00)	0.00(0.00,0.00)	1.55(1.22,1.88)	2.64(1.70,3.88)	8.15(4.84,12.67)	0.03(0.02,0.05)	0.04(0.02,0.06)	0.16(-0.04,0.36)
Ukraine	57.65(48.11,67.76)	32.31(21.72,45.69)	0.09(0.08,0.11)	0.05(0.04,0.08)	-2.93(-3.56,-2.30)	3008.85(2462.03,3579.74)	1805.31(1347.77,2370.52)	5.02(4.10,5.97)	3.14(2.35,4.15)	-2.46(-2.98,-1.94)
United Arab Emirates	0.06(0.02,0.12)	0.47(0.25,0.79)	0.01(0.00,0.01)	0.00(0.00,0.01)	-0.18(-0.69,0.33)	13.48(8.65,19.76)	123.86(80.48,184.73)	0.83(0.54,1.19)	0.79(0.53,1.15)	0.06(-0.04,0.16)
United Kingdom	188.35(162.97,216.12)	164.39(135.96,197.30)	0.25(0.22,0.29)	0.16(0.13,0.19)	-1.33(-1.48,-1.19)	9271.65(7806.42,10932.69)	8029.97(6390.58,9844.13)	13.41(11.28,15.86)	8.55(6.79,10.51)	-1.48(-1.55,-1.40)
United Republic of Tanzania	0.08(0.02,0.16)	0.28(0.08,0.56)	0.00(0.00,0.00)	0.00(0.00,0.00)	0.38(0.23,0.54)	12.78(8.23,18.70)	32.84(19.92,49.88)	0.09(0.06,0.13)	0.09(0.05,0.13)	-0.34(-0.51,-0.18)
United States of America	383.92(327.82,441.02)	559.20(463.13,667.31)	0.14(0.12,0.16)	0.11(0.09,0.13)	-1.05(-1.28,-0.83)	29025.25(23424.58,35662.58)	30512.52(24527.31,37553.61)	10.42(8.42,12.77)	6.79(5.43,8.43)	-1.46(-1.54,-1.38)
United States Virgin Islands	0.01(0.01,0.02)	0.01(0.01,0.02)	0.01(0.01,0.02)	0.01(0.00,0.01)	-0.21(-0.36,-0.05)	0.61(0.33,1.02)	0.61(0.38,0.99)	0.55(0.31,0.92)	0.51(0.31,0.82)	-0.08(-0.17,0.02)
Uruguay	2.22(1.85,2.59)	1.90(1.52,2.32)	0.06(0.05,0.07)	0.04(0.04,0.05)	-1.48(-1.79,-1.16)	112.73(91.04,134.87)	108.39(85.41,134.45)	3.41(2.74,4.07)	2.66(2.12,3.31)	-1.01(-1.27,-0.76)
Uzbekistan	0.52(0.35,0.72)	0.96(0.67,1.30)	0.00(0.00,0.01)	0.00(0.00,0.00)	0.86(0.05,1.68)	67.75(47.41,95.89)	155.07(110.69,217.99)	0.53(0.37,0.74)	0.46(0.33,0.65)	-0.20(-0.51,0.10)
Vanuatu	0.00(0.00,0.00)	0.00(0.00,0.00)	0.00(0.00,0.00)	0.00(0.00,0.00)	-0.61(-0.77,-0.45)	0.03(0.02,0.04)	0.06(0.03,0.09)	0.03(0.02,0.04)	0.02(0.01,0.03)	-0.61(-0.71,-0.51)
Venezuela (Bolivarian Republic of)	1.09(0.87,1.32)	3.96(2.67,5.47)	0.01(0.01,0.01)	0.01(0.01,0.02)	0.67(0.24,1.10)	55.77(43.63,68.69)	170.43(121.54,228.20)	0.45(0.35,0.55)	0.54(0.38,0.72)	0.44(0.09,0.79)
Viet Nam	0.09(0.02,0.17)	0.40(0.21,0.70)	0.00(0.00,0.00)	0.00(0.00,0.00)	1.43(1.03,1.83)	30.67(20.43,45.67)	94.62(63.96,140.86)	0.07(0.05,0.10)	0.08(0.06,0.12)	0.47(0.33,0.60)
Yemen	0.39(0.11,0.79)	2.66(1.36,4.99)	0.01(0.00,0.01)	0.01(0.01,0.03)	2.38(1.94,2.83)	67.15(44.27,95.86)	291.75(198.29,422.02)	0.95(0.63,1.37)	1.31(0.89,1.92)	0.98(0.84,1.13)
Zambia	0.02(0.00,0.04)	0.07(0.02,0.12)	0.00(0.00,0.00)	0.00(0.00,0.00)	0.17(0.02,0.33)	2.46(1.57,3.54)	6.40(4.03,9.53)	0.07(0.04,0.10)	0.06(0.04,0.09)	-0.56(-0.67,-0.45)
Zimbabwe	0.00(0.00,0.00)	0.00(0.00,0.00)	0.00(0.00,0.00)	0.00(0.00,0.00)	0.69(0.30,1.08)	5.65(3.55,8.62)	9.85(5.77,15.71)	0.10(0.07,0.16)	0.09(0.06,0.15)	-0.40(-0.46,-0.34)

Table S3 APC of Neurodegenerative diseases due to smoking from 1990 to 2021 for the World and SDI.

Characteristics		Death(95% CI)		DALYs(95% CI)	
		APCs	P	APCs	P
Dementia					
	slope1	-0.463(-0.542,-0.383)	*	-0.495(-0.622,-0.366)	*
	slope2	-1.241(-1.269,-1.214)	*	-1.202(-1.240,-1.164)	*
	slope3	-0.745(-0.772,-0.717)	*	-0.670(-0.696,-0.643)	*
	slope4	0.258(-0.212,0.730)		0.352(-0.224,0.931)	
Multiple sclerosis					
	slope1	-1.263(-1.377,-1.150)	*	-1.094(-1.402,-0.784)	*
	slope2	-2.829(-2.972,-2.685)	*	-1.602(-1.727,-1.476)	*
	slope3	-1.115(-2.194,-0.024)	*	-2.612(-2.736,-2.488)	*
	slope4	-3.067(-3.541,-2.590)	*	-2.017(-2.142,-1.891)	*

APC, Average percentage change; DALYs, Disability-adjusted life years;SDI, Socio-demographic Index.

Table S4 SDI-related health inequality slope index and relative concentration index for the global burden of neurodegenerative diseases due to smoking, 1990 and 2021.

Neurodegenerative diseases due to smoking	Dementias		Multiple sclerosis	
Characteristics	ASMR	ASDR	ASMR	ASDR
Inequality Slope Index				
1990	0.47(0.31, 0.64)	11.78(8.34, 15.22)	0.05(0.04, 0.06)	3.46(2.73, 4.19)
2021	0.34(0.22, 0.46)	8.60(6.10, 11.11)	0.05(0.04, 0.05)	3.16(2.60, 3.72)
Relative Concentration Index				
1990	0.13(0.08, 0.18)	0.14(0.10, 0.18)	0.62(0.56, 0.69)	0.60(0.54, 0.65)
2021	0.11(0.06, 0.16)	0.13(0.09, 0.17)	0.58(0.51, 0.64)	0.56(0.50, 0.61)

© **2026 Yang Z. et al.**
